# Fundamental limitations on distillation of quantum channel resources

**DOI:** 10.1038/s41467-021-24699-0

**Published:** 2021-07-20

**Authors:** Bartosz Regula, Ryuji Takagi

**Affiliations:** 1 School of Physical and Mathematical Sciences, Nanyang Technological University, Singapore, Singapore; 2 Center for Theoretical Physics and Department of Physics, Massachusetts Institute of Technology, Cambridge, MA USA

**Keywords:** Information theory and computation, Quantum physics, Quantum information

## Abstract

Quantum channels underlie the dynamics of quantum systems, but in many practical settings it is the channels themselves that require processing. We establish universal limitations on the processing of both quantum states and channels, expressed in the form of no-go theorems and quantitative bounds for the manipulation of general quantum channel resources under the most general transformation protocols. Focusing on the class of distillation tasks — which can be understood either as the purification of noisy channels into unitary ones, or the extraction of state-based resources from channels — we develop fundamental restrictions on the error incurred in such transformations, and comprehensive lower bounds for the overhead of any distillation protocol. In the asymptotic setting, our results yield broadly applicable bounds for rates of distillation. We demonstrate our results through applications to fault-tolerant quantum computation, where we obtain state-of-the-art lower bounds for the overhead cost of magic state distillation, as well as to quantum communication, where we recover a number of strong converse bounds for quantum channel capacity.

## Abstract (web summary)

Several key tasks in quantum information processing can be regarded as channel manipulation. Here, focusing on the class of distillation protocols, the authors derive general bounds on resource overhead and incurred errors, showing application to magic state distillation and quantum channel capacities.

## Introduction

One of the central aims of quantum information science is to precisely understand the limitations governing the use of quantum systems and develop the most efficient ways to take advantage of the laws of quantum physics. At the heart of such questions lies the study of quantum channels, which enable the manipulation of quantum states. However, in order to most effectively exploit quantum resources, it is important to be able to manipulate quantum channels themselves^1–3^. Channel transformations form the basis of some of the most pressing problems in quantum science, including for instance devising efficient schemes for quantum communication and key distribution for use in quantum networks^4–7^, or processing quantum circuits to aid in the mitigation and correction of errors in computation^8–10^.

Among such tasks, a particularly important class of problems known as channel distillation can be distinguished. Depending on the resource in consideration, distillation can be understood either as channel purification, i.e. the conversion of noisy channel resources into pure (unitary) ones, or as the extraction of state-based resources from quantum channels. The motivation for such transformations comes from the fact that, just as in the case of maximally entangled singlets in entanglement theory^4,11^, pure resources can be necessary for the efficient utilisation of a given resource. This is the case in quantum computation, where one aims to synthesise unitary quantum gates which can be employed in a quantum circuit^9^, or in quantum communication, where transfer of quantum information can be understood as the distillation of noiseless channels^7^. However, the practical realisation of such distillation protocols can incur large costs in terms of the required resource overhead. Due to the importance of distillation schemes in mitigating the effects of noise, the study of their limitations is therefore vital in many fundamental quantum information processing tasks. A major obstacle to understanding the capabilities of channel manipulation protocols is that general strategies for transforming channels can be highly complex, using ancillary systems and the outputs from successive channel uses in order to adaptively improve the transformations^2^, or even processing channels in ways that do not enforce a definite causal order^12,13^. Additionally, the limits of channel manipulation can be understood in different ways: in settings such as quantum computation, it is crucial to precisely understand and minimise the error incurred in manipulating gates and circuits, while in the study of quantum communication, it is often of interest to characterise asymptotic transformations and bound their achievable rates. We set to describe all such limitations in a common framework.

In this work, we establish a comprehensive approach to bounding the efficacy of manipulating the resources of quantum channels under general free transformations. We introduce universal lower bounds on the error of channel distillation, establishing precise quantitative limitations on the achievable performance of any distillation protocol. We reveal broad no-go results in multi-copy channel transformations under the most general manipulation protocols — adaptive schemes whose causal order structure is not necessarily fixed — allowing us to establish fundamental bounds on the overhead of any physical protocol for channel distillation and simulation. We furthermore use our results to provide strong converse bounds for asymptotic transformations, establishing sharp thresholds on the achievable distillation rates and characterising the ultimate limits of channel manipulation. All of our bounds rely on trade-off relations between the transformation accuracy and two important resource quantifiers: the resource robustness and resource weight. By adopting such a general resource-theoretic approach, our methods are readily applicable in a wide variety of practical settings. This allows us not only to unify, consolidate, and extend results that have appeared in specialised settings, but also to develop methods and bounds that have not previously found use in characterising resource transformations.

Furthermore, since quantum states can be regarded as a special case of quantum channels, our results apply also to state manipulation tasks. Our framework significantly improves on and extends the applicability of previous methods which characterised state transformations, including a recent general approach to no-go theorems and bounds for quantum state purification introduced in ref. ^14^.

Our results can be applied in the characterisation of general quantum resources, encompassing both intrinsic properties of quantum channels as well as dynamical resources based on the underlying properties of quantum states. We showcase this broad applicability with two different applications to the most pertinent settings: fault-tolerant quantum computation with magic states, as well as quantum communication. First, we connect the tasks of magic state distillation and gate synthesis through the underlying resource theory of magic, and study the similarities and differences between the two tasks. We show that our results yield substantially improved bounds in this setting, providing in particular state-of-the-art general lower bounds on the overhead of magic state distillation. We then develop further the resource-theoretic approach to quantum communication assisted by no-signalling correlations, where we show how our bounds can be used to understand both one-shot and asymptotic transformations as well as to recover the strong converse property of no-signalling coding^15,16^. Adapting our methods to the study of communication assisted by separable and positive partial transpose (PPT) operations, we recover a number of leading single-letter strong converse bounds on quantum capacity^17–19^, providing a simplification of proof methods employed in specialised approaches. Furthermore, we formalise the trade-off relations between the success probability and transformation accuracy in probabilistic distillation protocols where post-selection is allowed. Here, our results indicate a qualitative difference in achievable accuracy between deterministic and probabilistic settings, and suggest potential advantages of employing probabilistic distillation protocols.

## Results

### Setting

Quantum information processing can often be understood as the interplay of various physical resources^20,21^. In order to describe different quantum phenomena in a unified manner and establish methods that can apply to a broad variety of physical settings, we will employ the formalism of quantum resource theories^21^. The recent years have seen an active development of general resource-theoretic approaches to state manipulation and distillation problems, but the study of quantum channel manipulation in this setting is still in its infancy^3,22–25^. In particular, not much is known about constraining one-shot transformations of channels beyond specific settings, and questions such as transformation rates have previously only been addressed under specific assumptions on the structure of the involved resources and protocols. Our approach will be to instead employ broad resource-theoretic methods which avoid presupposing any particular properties of the considered setting.

A resource theory is a general framework concerned with the manipulation of quantum states or channels under some physical restrictions^21^. The restrictions determine which states or channels are ‘free’, in that they carry no resource and can be regarded as freely accessible under the physical constraints. The primary object of study of our work will be channel resources, so we assume that in the given physical setting, a particular subset of quantum channels ${\mathbb{O}}$ has been singled out as the free channels. A large number of very different settings and resources can be described with a suitable choice of ${\mathbb{O}}$, motivating us to establish methods that apply to any such choice. Therefore, to remain as general as possible, we will only make two natural assumptions about the set ${\mathbb{O}}$: that it is closed, meaning that no resource can be generated by taking a sequence of resourceless channels, and that it is convex, which means that simply probabilistically mixing free channels cannot generate any resource.

**Fig. 1:**
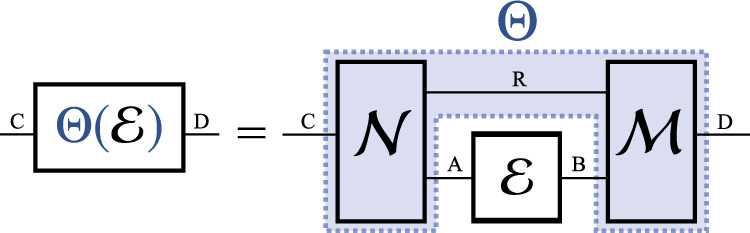
The general structure of a superchannel. Given the Hilbert spaces of two quantum systems *A* and *B*, we write CPTP(*A* → *B*) to denote the set of quantum channels, i.e. completely positive and trace-preserving (CPTP) maps between operators acting on spaces *A* and *B*. We associate with each channel ${\mathcal{E}}:A\to B$ its Choi matrix ${J}_{{\mathcal{E}}}:={\rm{id}}\otimes {\mathcal{E}}({{{\Phi }}}^{+})$, where ${{{\Phi }}}^{+}={\sum }_{i,j}\left|ii\right\rangle \left\langle jj\right|$ is the unnormalised maximally entangled state and id is the identity channel. Transformations of quantum channels are then maps from CPTP(*A* → *B*) to CPTP(*C* → *D*), or (*A* → *B*) → (*C* → *D*) in short. Under the necessary physical requirement that any such mapping should always take a quantum channel to a valid quantum channel, the most general form of a channel transformation is given by a quantum superchannel^1^. Such a transformation can be written as ${{\Theta }}({\mathcal{E}})={{\mathcal{M}}}_{RB\to D}\circ ({{\rm{id}}}_{R}\otimes {\mathcal{E}})\circ {{\mathcal{N}}}_{C\to RA}$ where ${\mathcal{N}},{\mathcal{M}}$ are some pre- and post-processing quantum channels and *R* denotes an ancillary system. For simplicity of notation, we often do not state explicitly which spaces the channels are acting on.

The most general way to manipulate a quantum channel is represented by a quantum superchannel^1^, which we introduce in Fig. 1. We are then interested in manipulating quantum channels with transformations which can be regarded as free within the constraints of the given theory. In order to apply our results to all possible settings, we will make no specific assumptions about the considered set of free superchannels, save for the weakest possible constraint: that a free transformation Θ does not generate any resource by itself; that is, for any free channel ${\mathcal{M}}\in {\mathbb{O}}$, it holds that ${{\Theta }}({\mathcal{M}})\in {\mathbb{O}}$. We use ${\mathbb{S}}$ to denote the set of all such resource-preserving superchannels. By studying these transformations, we will therefore obtain the most general bounds on the achievable performance of any free channel manipulation protocol, since any physically motivated choice of free transformations will necessarily be a subset of ${\mathbb{S}}$.

We stress that, as a special case, all of our results apply also to the manipulation of the static resources of quantum states: they can be viewed as quantum channels that act on a trivial input space. For clarity, we will use ${\mathbb{F}}$ instead of ${\mathbb{O}}$ to denote the set of free states when discussing state-specific applications.

### No-go theorems for resource distillation

The task of distillation can be understood as the transformation of a noisy resource channel ${\mathcal{E}}$ into ‘pure’ or ‘perfect’ resources, which are represented by some target channel ${\mathcal{T}}$. Importantly, two distinct types of channel resource theories can be distinguished. The first type is concerned with the investigation of intrinsic channel resources; this includes various resource theories of quantum communication and the related setting of quantum memories. In such cases, it is often natural to regard some unitary channel ${\mathcal{U}}(\cdot )=U\cdot {U}^{\dagger }$ as the target of distillation protocols, representing noiseless dynamical resources. The other type is concerned with an underlying state-based resource and the manipulation of channels in order to extract or utilise the state resource more effectively; this includes, for instance, quantum entanglement, coherence, or thermodynamics. The target can then be a replacement (or preparation) channel ${{\mathcal{R}}}_{\phi }(\cdot )={\rm{Tr}}(\cdot )\phi$ which prepares a given resourceful pure state. All of our results below apply to either of these settings, with ${\mathcal{T}}$ denoting a unitary or a replacement channel accordingly. Our task then is to understand when one can achieve transformations such that $F({{\Theta }}({\mathcal{E}}),{\mathcal{T}})\ge 1-\varepsilon$, where we use the worst-case fidelity^26,27^

1 $$F({\mathcal{E}},{\mathcal{F}})=\mathop{{\rm{min}}}\limits_{\rho }F({\rm{id}}\otimes {\mathcal{E}}(\rho ),{\rm{id}}\otimes {\mathcal{F}}(\rho ))$$

to effectively benchmark the error of the transformation. The choice of the worst-case fidelity as our figure of merit guarantees that the fidelity between the outputs of the channels will be large for any input state *ρ*, even when the channels are applied to a part of the system.

We endeavour to characterise the ultimate restrictions on the achievable performance of distillation by studying the trade-offs between three different quantities: the transformation error *ε*, the resources contained in the input channel ${\mathcal{E}}$, and the resources of the target channel. To this end, we will employ two different resource measures. The resource robustness ${R}_{{\mathbb{O}}}$^22,28–30^ and the resource weight ${W}_{{\mathbb{O}}}$^31,32^ are defined as

2 $${R}_{{\mathbb{O}}}({\mathcal{E}}):={\rm{min}}\left\{\lambda | \ {J}_{{\mathcal{E}}}\le \lambda {J}_{{\mathcal{M}}},\ {\mathcal{M}}\in {\mathbb{O}}\right\},$$

3 $${W}_{{\mathbb{O}}}({\mathcal{E}}):={\rm{max}}\left\{\lambda | \ {J}_{{\mathcal{E}}}\ge \lambda {J}_{{\mathcal{M}}},\ {\mathcal{M}}\in {\mathbb{O}}\right\},$$

where ${J}_{{\mathcal{E}}}$ is the Choi matrix of the given channel, and the inequality is understood in terms of positive semidefiniteness. The simple structure of the two quantities allows for a number of useful properties to be shown, such as their monotonicity under all free superchannels and submultiplicativity (see Supplementary Notes 1 and 3). The measures correspond to convex optimisation problems, in many relevant cases even reducing to efficiently computable semidefinite programs. ${R}_{{\mathbb{O}}}$ and ${W}_{{\mathbb{O}}}$ are natural generalisations of quantities defined at the level of quantum states, e.g. ${R}_{{\mathbb{F}}}(\rho )={\rm{min}}\left\{\lambda | \ \rho \ \le \ \lambda \sigma ,\ \sigma \in {\mathbb{F}}\right\}$, where we recall that ${\mathbb{F}}$ denotes free states in a considered resource theory. The robustness previously appeared in various ways in the characterisation of state transformations^29,33–35^, but the weight measure — although a known geometric resource quantifier — has not been connected with resource manipulation before.

To quantify the resources of the target channel, we will use the fidelity-based measure of the overlap with free channels:

4 $${F}_{{\mathbb{O}}}({\mathcal{T}}):=\mathop{{\rm{max}}}\limits_{{\mathcal{M}}\in {\mathbb{O}}}F({\mathcal{T}},{\mathcal{M}}).$$

This can be thought of as a parameter that determines how difficult a given target is to distil. Although we will show that this parameter can be straightforwardly computed in most cases of practical interest, in some contexts (such as quantum communication) an alternative figure of merit is often encountered: the Choi-state fidelity^36,37^

5 $${\tilde{F}}_{{\mathbb{O}}}({\mathcal{T}}):=\mathop{{\rm{max}}}\limits_{{\mathcal{M}}\in {\mathbb{O}}}\ F\left({\tilde{J}}_{{\mathcal{T}}},{\tilde{J}}_{{\mathcal{M}}}\right),$$

where we denoted by ${\tilde{J}}_{{\mathcal{E}}}$ the Choi matrix of a channel normalised so that ${\rm{Tr}}{\tilde{J}}_{{\mathcal{E}}}=1$. In our discussion below, we will state our results using the parameter ${F}_{{\mathbb{O}}}({\mathcal{T}})$ as this leads to the tightest bounds, but the bounds remain valid also if one replaces ${F}_{{\mathbb{O}}}({\mathcal{T}})$ with ${\tilde{F}}_{{\mathbb{O}}}({\mathcal{T}})$ everywhere.

We now give universally applicable, fundamental limitations on the performance of any resource distillation protocol.

#### Theorem 1

If there exists a free superchannel ${{\Theta }}\in {\mathbb{S}}$ such that $F({{\Theta }}({\mathcal{E}}),{\mathcal{T}})\ge 1-\varepsilon$ for a target channel ${\mathcal{T}}$, then

6 $$\varepsilon \ge 1-{F}_{{\mathbb{O}}}({\mathcal{T}})\ {R}_{{\mathbb{O}}}({\mathcal{E}})$$

and

7 $$\varepsilon \ge [1-{F}_{{\mathbb{O}}}({\mathcal{T}})]\ {W}_{{\mathbb{O}}}({\mathcal{E}}).$$

The bounds can be understood in two different ways: either as a general no-go result establishing the minimal error allowed within the constraints of the given resource theory, or, when *ε* is fixed, as a bound for the resources of ${\mathcal{E}}$ necessary for the distillation to be possible. The two bounds in Eqs. (6) and (7) are very different from each other, in both a quantitative and qualitative sense, and can complement each other in various settings. We will aim to elucidate this with explicit examples and discussions in the following sections and in the Supplementary Notes.

As an immediate consequence of the Theorem, we see that the exact transformation with *ε* = 0 is impossible whenever ${W}_{{\mathbb{O}}}({\mathcal{E}})\,> \, 0$, which is true e.g. for generic noisy channels with a full-rank Choi matrix. Importantly, channels with ${W}_{{\mathbb{O}}}({\mathcal{E}})\,> \, 0$ cannot be distilled to a pure target ${\mathcal{T}}$ even when the target is less resourceful. This indicates strong constraints on distillation characterised by the resource weight ${W}_{{\mathbb{O}}}$ and establishes a general no-go result in channel manipulation, extending earlier partial results^14^.

One important difference between the two bounds is that, when ${\mathcal{E}}$ is a pure (unitary or replacement) channel itself, then ${W}_{{\mathbb{O}}}({\mathcal{E}})=0$ and we gain no information from the weight bound. However, ${R}_{{\mathbb{O}}}({\mathcal{E}})$ can provide a non-trivial error threshold even in this case, making it useful also in unitary-to-unitary or pure-to-pure transformations.

The result of Theorem 1 directly applies also to the manipulation of quantum states, where now the free transformations ${\mathbb{S}}$ are in the form of quantum channels. Specifically, the bounds

8 $$\varepsilon \ \ge \ 1-{F}_{{\mathbb{F}}}(\phi )\ {R}_{{\mathbb{F}}}(\rho )$$

9 $$\varepsilon \ \ge \ [1-{F}_{{\mathbb{F}}}(\phi )]\ {W}_{{\mathbb{F}}}(\rho )$$

hold for any state *ρ* undergoing a distillation protocol with a pure state *ϕ* as a target. This gives general error bounds on transformations of state-based resources. While the state-based robustness bound has previously appeared in ref. ^34^, the weight bound constitutes an improvement over previously known results, and in particular over a different approach to no-go theorems for resource purification which was recently introduced in ref. ^14^. In contrast to the framework of ref. ^14^, our results can characterise the manipulation of all quantum states (not only full-rank input states) and our quantitative bounds are strictly better than the previously known ones. This allows us to reveal substantially refined limitations on state-to-state transformations, as we will shortly demonstrate in explicit comparisons.

We will find that the bounds can tightly characterise one-shot transformations for specific cases of channels. However, a major strength of the bounds lies not simply in estimating the errors in single-shot channel manipulation, but also in their applicability to multi-copy and asymptotic manipulation protocols: we now show that the bounds of Theorem 1 can reveal powerful restrictions on distillation when multiple uses of a quantum channel are considered.

### Many-copy manipulation

**Fig. 2:**
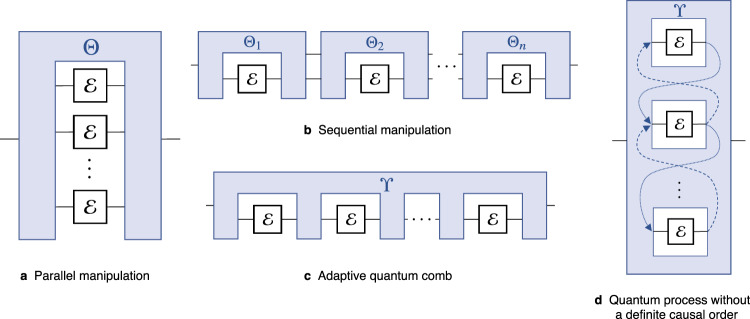
The different ways to manipulate many copies of a quantum channel with free transformations. For quantum states, having access to multiple copies of a state *ρ* is equivalent to acting on the tensor product *ρ*^⊗*n*^. A naive way to employ *n* copies of a given channel is to consider them in parallel as ${{\mathcal{E}}}^{\otimes n}$(**a**). However, a more general way to manipulate *n* copies of a channel is to employ a sequential (iterative) protocol (**b**), which can be understood as the channel ${\mathcal{E}}$ being fed into a sequence of *n* free superchannels one after another, allowing one to use the output of the previous channel uses to improve the transformation. Indeed, such protocols are known to provide advantages over parallel ones in some settings^23,76–79^, and this approach is commonly employed to transform channels in the setting of various resource theories such as quantum communication^17,80–82^, entanglement^83,84^, or magic^48^. However, even this does not represent the most general way to manipulate multiple channels within the setting of the given resource theory. When the causal order of the channels is fixed, any *n*-channel transformation scheme by means of a quantum circuit can be expressed as a so-called quantum comb^2^(**c**). Even more complex manipulation strategies are possible if one does not assume a definite causal order between the channel transformations, that is, when one is not able to say in what order the channels will be used throughout the protocol (**d**). Such an approach allows one to treat the transformation trajectories themselves as quantum objects, leading to concepts such as superpositions of different causal orders^12,13^ which can indeed provide advantages over standard, causally ordered transformation methods^12,79,85^. These transformations are dubbed quantum processes, and we will use them to characterise the most general physically realisable manipulation protocols involving multiple quantum channels.

In contrast to transformations of quantum states, it does not suffice to consider channel manipulation as acting on the tensor product ${{\mathcal{E}}}^{\otimes n}$, but more complex protocols need to be considered. The most general form of such manipulation schemes are referred to as quantum processes (see Fig. 2). We then use ${{\mathbb{S}}}_{(n)}$ to denote all free quantum processes, that is, all transformations ϒ of *n* channels such that the output channel ${{\Upsilon }}({{\mathcal{M}}}_{1},\ldots ,{{\mathcal{M}}}_{n})$ is a free channel whenever ${{\mathcal{M}}}_{1},\ldots ,{{\mathcal{M}}}_{n}\in {\mathbb{O}}$. This approach will allow us to characterise the performance of the most general protocols for manipulating channels or states within the physical constraints of the given resource.

#### Theorem 2

Given any distillation protocol ${{\Upsilon }}\in {{\mathbb{S}}}_{(n)}$ — parallel, sequential, or adaptive, with or without a definite causal order — which transforms *n* uses of a channel ${\mathcal{E}}$ to some target channel ${\mathcal{T}}$ up to accuracy *ε* > 0, it necessarily holds that

10 $$n\ \ge \ {{\rm{log}}}_{1/{W}_{{\mathbb{O}}}({\mathcal{E}})}\ \frac{1-{F}_{{\mathbb{O}}}({\mathcal{T}})}{\varepsilon }$$

and

11 $$n\ \ge \ {{\rm{log}}}_{{R}_{{\mathbb{O}}}({\mathcal{E}})}\ \frac{1-\varepsilon }{{F}_{{\mathbb{O}}}({\mathcal{T}})}.$$

This gives general lower bounds on the overhead of distillation that must be obeyed by any physical transformation protocol. Once again, the bounds exhibit different behaviour: intuitively, the regime of *ε* very close to 0 will be characterised more precisely by the bound based on resource weight ${W}_{{\mathbb{O}}}$, while the robustness ${R}_{{\mathbb{O}}}$ will perform better for larger error and for input channels ${\mathcal{E}}$ which are close to pure (unitary or replacement) channels.

An important aspect of the bound in Theorem 2 is that it holds regardless of the structure of the involved channel manipulation process ϒ. This allows us to go beyond methods previously employed in settings such as quantum communication, which applied only to sequential protocols with a restricted structure.

As an immediate consequence of this result, the weight-based bound in Theorem 2 shows that the number of uses of the channel ${\mathcal{E}}$ needed to perform distillation must scale as ${\mathrm{log}}\,(1/\varepsilon )$ as *ε* → 0, establishing a universal limit on the overhead of distillation protocols such as quantum gate synthesis or noisy quantum communication.

### Asymptotic manipulation

The ultimate limitations on transforming a given state or channel are given by the maximal rate at which the conversion ${\mathcal{E}}\to {\mathcal{T}}$ can be performed with an asymptotic number of channel uses, allowing for error that vanishes asymptotically. Specifically, we will be interested in protocols which transform *n* uses of a quantum channel ${\mathcal{E}}$ to *rn* copies of the target channel ${{\mathcal{T}}}^{\otimes rn}$ up to error *ε*_*n*_. Imposing that the transformation is achieved exactly in the asymptotic limit, that is, *ε*_*n*_ → 0 as *n* → *∞*, and maximising over all such *r* gives us the optimal asymptotic rate of converting ${\mathcal{E}}$ to ${\mathcal{T}}$ with free protocols. We will distinguish two different rates: an adaptive rate *r*_adap_ which allows the most general, adaptive processes acting on the input channels, and the parallel rate *r*_par_ which considers parallel transformations of the form ${{\mathcal{E}}}^{\otimes n}\to {{\mathcal{T}}}^{\otimes rn}$ (recall the comparison in Fig. 2).

The rates of distillation of quantum channel resources are an important aspect of understanding the limitations on resource manipulation^23,30,38^, but little is known about them due to the difficulty in characterising the asymptotic properties of channel-based quantities^25,39^. Our methods allow us to establish two general bounds on the transformation rates. We can use the robustness ${R}_{{\mathbb{O}}}$ to provide a general bound for the rate of any manipulation protocol, as well as obtain an improved bound for parallel protocols by suitably ‘smoothing’ the definition of the robustness over channels within a small distance of the original input ${\mathcal{E}}$^15,24,25,30^.

#### Theorem 3

If the target channel ${\mathcal{T}}$ satisfies ${F}_{{\mathbb{O}}}({{\mathcal{T}}}^{\otimes n})={F}_{{\mathbb{O}}}{({\mathcal{T}})}^{n}$, then

12 $${r}_{{\rm{adap}}}({\mathcal{E}}\to {\mathcal{T}})\ \le \ \frac{{\rm{log}}{R}_{{\mathbb{O}}}({\mathcal{E}})}{{\rm{log}}{F}_{{\mathbb{O}}}{({\mathcal{T}})}^{-1}},$$

13 $${r}_{{\rm{par}}}({\mathcal{E}}\to {\mathcal{T}})\ \le \ \frac{{D}_{{\mathbb{O}}}^{\infty }({\mathcal{E}})}{{\rm{log}}{F}_{{\mathbb{O}}}{({\mathcal{T}})}^{-1}},$$

where $${D}_{{\mathbb{O}}}^{\infty }({\mathcal{E}}):={{\rm{lim}}}_{\delta \to 0}{\rm{lim}}\ {{\rm{sup}}}_{n\to \infty }\frac{1}{n}{\rm{log}}{R}_{{\mathbb{O}}}^{\delta }({{\mathcal{E}}}^{\otimes n})$$ with $${R}_{{\mathbb{O}}}^{\delta }({\mathcal{E}}):={{\rm{min}}}_{F(\tilde{{\mathcal{E}}},{\mathcal{E}})\ge 1-\delta }{R}_{{\mathbb{O}}}(\tilde{{\mathcal{E}}})$$.

The result establishes universal bounds on the achievable rate under any physical transformation protocol. Importantly, both of our bounds are strong converse bounds, that is, they sharply characterise the threshold in achievable performance — when a rate exceeds either of our bounds, the transformation fidelity necessarily goes to 0, meaning that the error will grow very large and distillation cannot be reliably performed. The Theorem immediately applies in many settings of practical significance, as long as the condition ${F}_{{\mathbb{O}}}({{\mathcal{T}}}^{\otimes n})={F}_{{\mathbb{O}}}{({\mathcal{T}})}^{n}$ is satisfied for the given target channel. This is a natural property that holds true both in dynamical resources such as communication, as well as in state-based channel resources such as entanglement, magic, coherence, or thermodynamics (see the forthcoming Table 1).

In the majority of practically relevant settings, the robustness ${R}_{{\mathbb{O}}}$ is submultiplicative under tensor product, meaning that ${D}_{{\mathbb{O}}}^{\infty }({\mathcal{E}})\le {\mathrm{log}}\,{R}_{{\mathbb{O}}}({\mathcal{E}})$. Hence, the bound on *r*_par_ using ${D}_{{\mathbb{O}}}^{\infty }({\mathcal{E}})$ might provide an improvement over the robustness-based bound, prompting the question of whether one can actually evaluate the tighter bound. Notably, the regularisation ${D}_{{\mathbb{O}}}^{\infty }({\mathcal{E}})$ has been computed exactly for a set of channels relevant in the study of quantum communication^15^, which we will discuss in more detail shortly. The recent work of ref. ^25^ began a systematic investigation of different regularisations in channel-based resource theories, but their general computability remains an open question. For quantum states, the regularisation ${D}_{{\mathbb{F}}}^{\infty }(\rho )={{\rm{lim}}}_{\delta \to 0}{{\rm{lim}}}_{n\to \infty }\frac{1}{n}{\mathrm{log}}\,{R}_{{\mathbb{F}}}^{\delta }({\rho }^{\otimes n})$ can be computed exactly under very mild assumptions on the set ${\mathbb{F}}$^33^, and it reduces to the regularised relative entropy of a resource. In such cases, our result recovers the fact that rates of distillation in resource theories of states are limited by the regularised relative entropy^33,40^. We note also that related asymptotic bounds were considered in ref. ^23^ for the case of state-based channel resource theories.

### Applying the bounds in practice

We stress again that our main results discussed in Theorems 1–3 apply to general convex resource theories of quantum channels and states, encompassing a wide variety of use cases. Since our discussions so far have presented them in a rather abstract manner, we will now discuss how the bounds can be evaluated in specific theories of interest.

With the exception of the regularised asymptotic bound in Theorem 3, all of our results depend only on three quantities: the overlap ${F}_{{\mathbb{O}}}({\mathcal{T}})$ of the target channel, and either the robustness ${R}_{{\mathbb{O}}}({\mathcal{E}})$ or the weight ${W}_{{\mathbb{O}}}({\mathcal{E}})$ of the input. In practical settings of interest, the choice of the target ${\mathcal{T}}$ is motivated by physical considerations — representing, for instance, a maximally resourceful channel or state, or a particularly costly resource — and the value of the parameter ${F}_{{\mathbb{O}}}({\mathcal{T}})$ is typically known, so we can directly plug these quantities into the bounds established in Theorems 1–3. We collect some of the most important examples of such resources, together with the values of ${F}_{{\mathbb{O}}}({\mathcal{T}})$, in Table 1. All that remains now is to evaluate ${R}_{{\mathbb{O}}}$ or ${W}_{{\mathbb{O}}}$ for desired input channels. Fortunately, in many theories of interest, these two quantifiers can be computed as semidefinite programs, and often even evaluated or bounded analytically by utilising their convex duality and constructing suitable feasible solutions.

**Table 1:** Applicability of our bounds to common quantum resources.

Channel resource	Target channel ${\mathcal{U}}$	${F}_{{\mathbb{O}}}({\mathcal{U}})$	${F}_{{\mathbb{O}}}({{\mathcal{U}}}^{\otimes m})\ \mathop{=}\limits^{?}\ {F}_{{\mathbb{O}}}{({\mathcal{U}})}^{m}$	Computability of ${R}_{{\mathbb{O}}}$ and ${W}_{{\mathbb{O}}}$
Quantum communication assisted by:				
no-signalling transformations^53^	Identity channel id_*d*_	$\frac{1}{{d}^{2}}$	Yes	SDP
separability-preserving transformations	Identity channel id_*d*_	$\frac{1}{d}$^72^	Yes	Convex program (NP-hard)
PPT-preserving transformations	Identity channel id_*d*_	$\frac{1}{d}$^58^	Yes	SDP
Magic of many-qubit quantum channels^46^	Qubit T gate *T* = diag(1, *e*^*iπ*/4^)	$\frac{1}{4}(2+\sqrt{2})$^49^	Yes^49^	SDP
	Controlled-controlled-Z gate	$\frac{9}{16}$^49^	Yes^49^	SDP
Magic of many-qudit quantum channels^48^	Qutrit T gate *T* = diag(*e*^2*πi*/9^, 1, *e*^−2*πi*/9^)	${(1+2\sin (\pi /18))}^{-1}$^48^	Yes^47^ *	SDP

State resource	Target state $\left|\phi \right\rangle$	${F}_{{\mathbb{F}}}(\phi )$	${F}_{{\mathbb{F}}}({\phi }^{\otimes m})\ \mathop{=}\limits^{?}\ {F}_{{\mathbb{F}}}{(\phi )}^{m}$	Computability of ${R}_{{\mathbb{F}}}$ and ${W}_{{\mathbb{F}}}$
Quantum entanglement^73^	Maximally entangled state $\frac{1}{\sqrt{d}}\mathop{\sum }\nolimits_{i = 0}^{d-1}\left|ii\right\rangle$	$\frac{1}{d}$^72^	Yes	Convex program (NP-hard)
Non-positive partial transpose^73^	Maximally entangled state $\frac{1}{\sqrt{d}}\mathop{\sum }\nolimits_{i = 0}^{d-1}\left|ii\right\rangle$	$\frac{1}{d}$^58^	Yes	SDP
Quantum coherence^74^	Maximally coherent state $\frac{1}{\sqrt{d}}\mathop{\sum }\nolimits_{i = 0}^{d-1}\left|i\right\rangle$	$\frac{1}{d}$	Yes	SDP
Magic of many-qubit states^44,45^	T state $\frac{1}{\sqrt{2}}(\left|0\right\rangle +{e}^{i\pi /4}\left|1\right\rangle )$	$\frac{1}{4}(2+\sqrt{2})$^49^	Yes^49^	SDP
	CCZ state $\frac{1}{8}{(1,\ldots ,1,-1)}^{T}$	$\frac{9}{16}$^49^	Yes^49^	SDP
Magic of many-qudit states^44^	Hadamard ‘+’ state $\propto (1+\sqrt{3})\left|0\right\rangle +\left|1\right\rangle +\left|2\right\rangle$	$\frac{1}{6}(3+\sqrt{3})$^47^	Yes^47^	SDP
	Norrell state $\frac{1}{\sqrt{6}}(\left|0\right\rangle -2\left|1\right\rangle +\left|2\right\rangle )$	$\frac{2}{3}$^47^	Yes^47^	SDP
Quantum thermodynamics with Hamiltonian $H={\sum }_{i}{E}_{i}\left|i\right\rangle \left\langle i\right|$^75^	Energy eigenstate $\left|i\right\rangle$	$\frac{{e}^{-\beta {E}_{i}}}{Z}$ (*β*: inverse temp., *Z*: partition function)	Yes	Analytical

We give an overview of quantum resources together with natural choices of target channels or states which are often used in distillation tasks. The list is by no means complete but is meant to facilitate the application of our bounds in a selection of important settings. We see in particular that the parameter ${F}_{{\mathbb{O}}}({\mathcal{T}})$ admits an exact analytical expression for all the target states on the list, and in addition is multiplicative under tensor product, which means that all of the bounds of this work (including the asymptotic bounds of Theorem 3) apply immediately. Furthermore, we see that the majority of cases discussed here allow for the robustness and weight measures to be computed as semidefinite programs (SDP). In the main text and in Supplementary Note 4, we provide more details about the theories of quantum communication and the magic of channels and states, showing exactly how the bounds can be applied and how they perform.

^*^ In the case of the qutrit *T* gate, instead of the quantity ${F}_{{\mathbb{O}}}$ as defined in Eq. (4), a closely related fidelity-type measure called the ‘min-thauma’^47^ is used, which allows for an easier computation while otherwise acting in the same way. This makes no difference in the statement or properties of our bounds, so we make no distinction here and instead refer to Supplementary Note 4 for details.

It will be instructive to discuss in more detail the applications to two fundamental examples. Full technical details and additional results are provided in Supplementary Note 4.

### Application: gate synthesis and magic state distillation

Universal fault-tolerant quantum computation requires, in addition to the easily implementable Clifford gates, the use of costly non-Clifford unitaries such as the T gate^41^. Such gates are often implemented through the process of magic state injection^42^, which employs magic (non-stabiliser) states — states that cannot be obtained with stabiliser operations alone — to realise general quantum gates. Magic states can provide feasible ways to synthesise general quantum circuits, but the main bottleneck in their efficient use is the resource cost associated with the required magic state distillation protocols^9^. Understanding the limitations of such protocols and characterising the precise relations between magic state distillation and gate synthesis is thus highly important in paving the way to fault-tolerant quantum computation^9,43^.

In this setting, our results can be employed in two different ways: either directly at the level of channel manipulation (gate synthesis), or through an application to the task of magic state distillation. They therefore advance the resource-theoretic approach to magic^44–48^ by explicitly shedding light on the precise quantitative connections between the channel-based theory and the underlying state-based resource. Here, the set of free channels ${\mathbb{O}}$ can be understood as all stabiliser operations, or the larger set of completely stabiliser-preserving operations^46^. We can then directly apply Theorems 1**–**3 to immediately establish a number of bounds which can characterise the ultimate limitations in both exact and approximate transformations between channels and states in these resource theories. The relevant quantities ${R}_{{\mathbb{O}}}$, ${W}_{{\mathbb{O}}}$, and ${F}_{{\mathbb{O}}}$ are computable as semidefinite programs in this setting, and for many channels of interest, such as quantum gates from the third level of the Clifford hierarchy^42^, the measures simplify to known quantities like the state-based stabiliser fidelity ${F}_{{\mathbb{F}}}$^49^ (see Supplementary Note 4). Applied at the level of states, our approach — and in particular the weight-based bound — constitutes a substantial improvement over the recent findings of refs. ^14,50^ where lower bounds on the resource cost of magic state distillation were established.

**Fig. 3:**
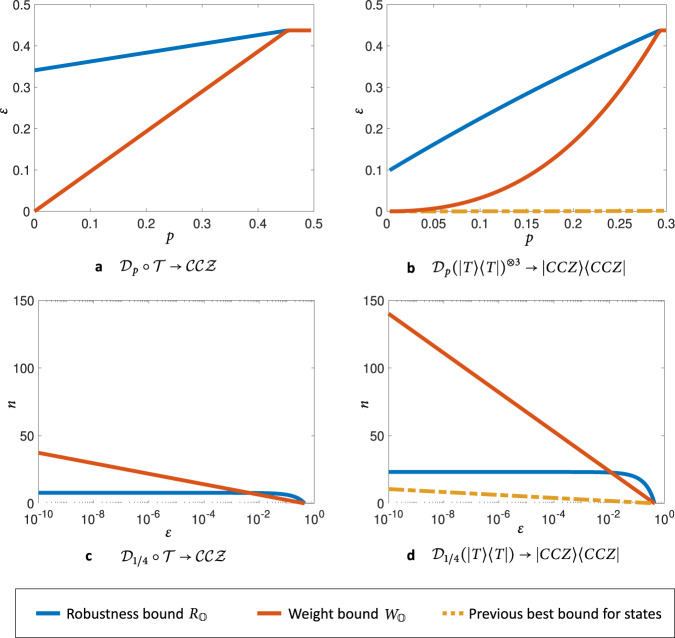
Bounding the performance of gate synthesis and magic state distillation. We plot lower bounds on: **a**, **b** the error *ε* necessarily incurred (as per Theorem 1); **c**, **d** the number of copies necessary (as per Theorem 2) in the given transformations between the depolarised T gate/state and the depolarised CCZ gate/state. The bounds are compared with the previous best general bound for magic state distillation introduced in ref. ^14^. Here, *p* is the noise parameter of the depolarising channel ${{\mathcal{D}}}_{p}(\rho )=(1-p)\rho +p\frac{{\mathbb{1}}}{2}$. In (**a**) we explicitly see that the robustness bound indicates a significant error also in the noiseless case (*p* = 0), whereas the weight bound becomes trivial for noiseless inputs. In (**b**) we allow three copies of the noisy *T* state to be used in the transformation here, both of our bounds significantly improve on the results of ref. ^14^, and in particular the robustness bound reveals that an error of ≈ 0.1 is the best that one can hope for when converting ${\left|T\right\rangle }^{\otimes 3}\to \left|CCZ\right\rangle$ with any free transformation protocol. In (**c**) and (**d**) we demonstrate the substantial advantages of the weight bound in bounding distillation overhead. Comparing the bounds for gate synthesis from the noisy *T* gate in (**c**) and for magic state distillation from the noisy *T* state in (**d**) we can see that the bounds impose much higher requirements on the number of noisy states required to succeed.

Our results are demonstrated in Fig. 3, where we plot the performance of our bounds in the transformation of the T gate *T* = diag(1, *e*^*iπ*/4^)^41^ or the associated $\left|T\right\rangle$ state, affected by depolarising noise, to the controlled-controlled-Z gate CCZ. We see that our results give non-trivial bounds on the error in all parameter regimes, revealing large errors even in cases where previous bounds could not do so. Notably, this yields state-of-the-art lower bounds on the overhead of magic-state distillation, as well as general bounds directly for the task of quantum gate synthesis.

### Application: quantum communication

The quantum capacity $Q({\mathcal{E}})$ characterises the rate at which quantum information can be communicated through a channel, and bounding this quantity is a fundamental problem in quantum communication^40,51,52^. It is often useful to allow the communicating parties to use some assistance — in the form of shared correlations, or the ability to perform some limited set of joint operations — in order to aid the communication. This traditional setting of quantum communication can be encompassed in our resource-theoretic framework of channel manipulation: the goal can be understood as using free superchannels (encoding and decoding operations) in order to purify a noisy quantum channel to the qubit identity channel id_2_, with the latter representing perfect noiseless communication. Here, we will see that our general results can be readily applied to assess several fundamental limitations in this task.

**Fig. 4:**
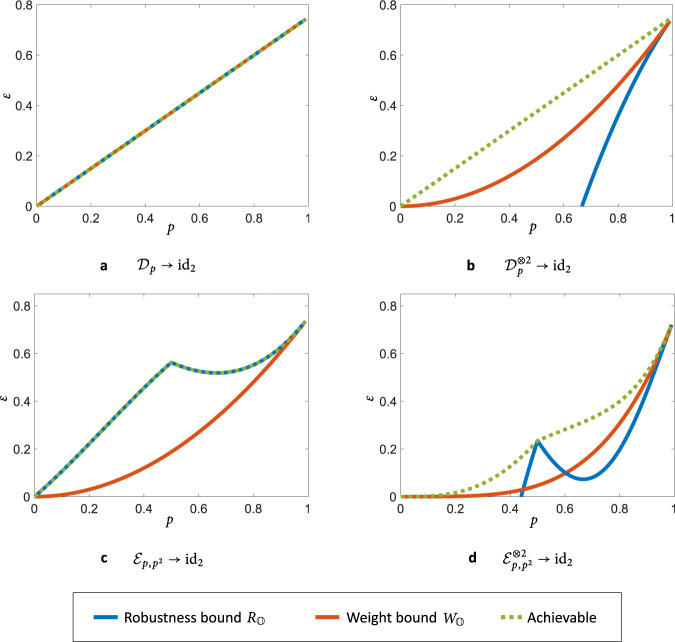
Errors in one-shot quantum communication. Lower bounds on the error *ε* in the transformation of channels to the qubit identity channel id_2_ under no-signalling codes $${{\mathbb{S}}}_{{\rm{NS}}}$$. We plot the bounds obtained from Theorem 1 for: **a**, **b** the qubit depolarising channel $${{\mathcal{D}}}_{p}(\rho )=(1-p)\rho +p\frac{{\mathbb{1}}}{d}$$; (**c**, **d**) the dephrasure channel $${{\mathcal{E}}}_{p,q}(\rho )=(1-q)[(1-p)\rho +pZ\rho Z]$$$$+\ q\left|2\right\rangle \ \left\langle 2\right|$$^86^, where we set *q* = *p*^2^. In (**a**) the depolarising channel satisfies $${W}_{{\mathbb{O}}}({{\mathcal{D}}}_{p})=p$$, and the robustness and weight-based bounds are actually equal: we have *ε* ≥ *p*(*d*^2^ − 1)/*d*^2^. In fact, here the bounds match the achievable fidelity, meaning that Theorem 1 quantifies the error in the one-shot transformation $${{\mathcal{D}}}_{p}\to {{\rm{id}}}_{2}$$ under $${{\mathbb{S}}}_{{\rm{NS}}}$$ exactly. The robustness bound is also seen to be tight in (**c**). The weight bound can become more effective than the robustness bound when we consider more copies of the input channel, as seen in (**b**) and (**d**). The importance of the weight bound is highlighted here, as it can certify that zero-error (*ε* = 0) communication is impossible for all *p* > 0.

For instance, the setting of no-signalling (NS) assisted communication^15,16,53–55^ allows Alice and Bob to perform joint coding protocols which obey the no-signalling condition from Alice to Bob and vice versa. First, it is insightful to see what the bounds of Theorem 1 tell us about one-shot transformations ${\mathcal{E}}\to {{\rm{id}}}_{2}$ in this setting. Here, the maximal fidelity achievable under no-signalling codes can be computed with an SDP^53^, which allows us to gauge the performance of our bounds exactly. We demonstrate this with a numerical investigation in Fig. 4, showing that our results can become exact in some cases, and the two bounds can complement each other in different situations. Beyond such single-shot transformations, when multiple uses of channels are considered, our bounds can lead to tight asymptotic results. In particular, Theorem 3 gives a strong converse bound on the NS-assisted quantum capacity as ${Q}_{{\rm{NS}}}({\mathcal{E}})\le {D}_{{\mathbb{O}}}^{\infty }({\mathcal{E}})$. Importantly, the quantity ${D}_{{\mathbb{O}}}^{\infty }({\mathcal{E}})$ can be computed exactly in this case^15^, and it equals the mutual information of the channel^56^. Moreover, this is actually an achievable rate of communication^53,56^, which means that ${Q}_{{\rm{NS}}}({\mathcal{E}})$ is given exactly by the mutual information of ${\mathcal{E}}$. In addition to recovering this tight bound, our results also show the strong converse property of NS-assisted communication^15,16^, which says that the capacity ${Q}_{{\rm{NS}}}({\mathcal{E}})$ is a strong converse rate of communication.

Another fundamental paradigm is quantum communication assisted by local operations and classical communication (LOCC)^4^. Due to the complexity of describing LOCC, various approximations of this class of channels are often employed^40,53,57–59^, the most common ones being the set of separable channels ${{\mathbb{O}}}_{{\rm{SEP}}}$^57^ (maps whose Choi matrix is separable) and positive partial transpose (PPT) channels ${{\mathbb{O}}}_{{\rm{PPT}}}$^58^ (maps whose Choi matrix is PPT). We can readily apply our results in two different ways, by considering either the capacity *Q*_SEP_ of communication assisted by protocols which preserve ${{\mathbb{O}}}_{{\rm{SEP}}}$, or the capacity *Q*_PPT_ where Alice and Bob can perform joint manipulation protocols which preserve ${{\mathbb{O}}}_{{\rm{PPT}}}$. Theorems 1–3 then immediately provide a number of bounds on quantum capacity assisted by the most general adaptive protocols, in both the one-shot and asymptotic settings. Notably, we obtain that the robustness ${R}_{{{\mathbb{O}}}_{{\rm{SEP}}}}$ gives a strong converse bound to *Q*_SEP_. We show, in fact, that the robustness in this case equals a quantity known as the max-relative entropy of entanglement^17^, therefore recovering and extending a bound of ref. ^17^, while providing an arguably simpler proof technique. In the PPT setting, we get an analogous result, which is closely related to bounds based on the so-called max-Rains information^18,19^ — these constitute, in many cases, the best known efficiently computable bounds on LOCC-assisted capacity.

The above shows the direct applicability of our formalism to upper bounding channel capacities in a number of different settings. Our methods thus not only provide useful benchmarks of practical significance, but also unify different specialised approaches and show them all to be part of a broader, resource-theoretic framework for the manipulation of quantum channels, which extends beyond entanglement and communication theory.

### Extension to probabilistic protocols

Our results can also be extended to the setting where the channel transformations are not realised deterministically, but can fail with a certain probability. Here, we establish general trade-offs between the success probability and the error in the transformation, extending the insights and establishing bounds that take into account the non-deterministic character of the transformations. The results suggest that potential advantages of probabilistic protocols over deterministic ones can be found in some cases. Because of the technicality of such extensions caused by the more complicated nature of probabilistic channel transformations^60^, we defer the details to the Methods and Supplementary Note 5.

## Discussion

We introduced universal quantitative bounds on the achievable performance of any deterministic distillation protocol in general quantum resource theories. We showed that our bounds can be used to establish fundamental no-go relations in the manipulation of quantum channels, introduce powerful restrictions on the overhead of any physical distillation protocol using the most general, adaptive manipulation schemes with indefinite causal order, and lead to several strong converse bounds for the asymptotic transformations of channels. We demonstrated the versatility of our bounds through applications to quantum communication and stabiliser-state quantum computation, using our bounds to characterise these important resource theories. Finally, we extended the insights provided by our bounds to distillation protocols which allow probabilistic implementations.

A key feature of our bounds is their generality: using a general resource-theoretic approach, we were able to establish limitations on the manipulation of quantum resources under only the most basic assumptions enforced by the structure of quantum mechanics. This reveals common aspects shared by all types of quantum resources, establishing our bounds as fundamental quantitative limitations on channel manipulation. Importantly, however, such breadth of this approach does not diminish its usefulness in concrete settings of interest — we have shown that all of our bounds can be directly applied in a multitude of relevant resources, and we expect them to find use also in many settings that we have not considered here explicitly. On the practical side, our results shed light in particular on the important problem of purifying noisy resources. Because of the unavoidably noisy character of near-term quantum technologies^10^, such distillation schemes are often necessary, and we therefore anticipate our bounds to find use in the practical investigation of the limitations of quantum information processing in a broad range of settings such as channel discrimination^61,62^, noise estimation^63^, programming of quantum channels^64,65^, and covariant error correction^66–68^.

An interesting direction to consider in further research would be to understand precisely when and how the bounds can be achieved in practical setups, and how the different types of transformations — parallel, adaptive, or ones with an indefinite causal order — perform in various types of manipulation tasks. It would also be intriguing to apply our methods to the transformation of other types of dynamical resources, such as Bell nonlocality or quantum contextuality, which may provide further operational insights into the fundamental advantages enabled by quantum theory in different settings.

### Note

During the completion of this paper, we became aware of a related work by Fang and Liu^69^ where the authors independently considered the resource weight and obtained results related to the weight-based bounds in our Theorems 1 and 2.

## Methods

We give an overview of the main techniques used to establish our results in Theorems 1–3. The complete technical details, along with additional discussion and extensions, can be found in the Supplementary Information.

### One-shot bounds (Theorem 1)

We will use the fact that both the robustness and weight measures can be expressed in terms of the max-relative entropy ${D}_{{\rm{max}}}(\rho \parallel \sigma ):={\mathrm{log}}\,\inf \left\{\lambda | \ \rho \ \le \ \lambda \sigma \right\}$^70^. Defining ${R}_{{\rm{max}}}(\rho \parallel \sigma ):={2}^{{D}_{{\rm{max}}}(\rho \parallel \sigma )}$, for any channels ${\mathcal{E}},{\mathcal{F}}:A\to B$ one can define the optimised channel divergence^71^

14 $${R}_{{\rm{max}}}({\mathcal{E}}\parallel {\mathcal{F}}):=\mathop{{\rm{max}}}\limits_{\psi }{R}_{{\rm{max}}}({\rm{id}}\otimes {\mathcal{E}}(\psi )\parallel {\rm{id}}\otimes {\mathcal{F}}(\psi )).$$

This generalisation of the max-relative entropy to channels obeys some useful properties, and in particular it holds that^71^

15 $${R}_{{\rm{max}}}({\mathcal{E}}\parallel {\mathcal{F}})={R}_{{\rm{max}}}({J}_{{\mathcal{E}}}\parallel {J}_{{\mathcal{F}}}),$$

that is, it suffices to consider the Choi matrices of the channels to evaluate the max-relative entropy. Exploiting the properties of *R*_max_ and the convex structure of the involved optimisation, we can then express the robustness measure ${R}_{{\mathbb{O}}}$ as

16 $${R}_{{\mathbb{O}}}({\mathcal{E}})=\mathop{{\rm{min}}}\limits_{{\mathcal{M}}\in {\mathbb{O}}}{R}_{{\rm{max}}}({\mathcal{E}}\parallel {\mathcal{M}})\qquad\qquad\qquad\qquad\quad\qquad\;\,$$

17 $$=\mathop{{\rm{min}}}\limits_{{\mathcal{M}}\in {\mathbb{O}}}\mathop{{\rm{max}}}\limits_{\psi }{R}_{{\rm{max}}}({\rm{id}}\otimes {\mathcal{E}}(\psi )\parallel {\rm{id}}\otimes {\mathcal{M}}(\psi ))$$

18 $$=\mathop{{\rm{max}}}\limits_{\psi }\mathop{{\rm{min}}}\limits_{{\mathcal{M}}\in {\mathbb{O}}}{R}_{{\rm{max}}}({\rm{id}}\otimes {\mathcal{E}}(\psi )\parallel {\rm{id}}\otimes {\mathcal{M}}(\psi )).$$

In a very similar way, the weight ${W}_{{\mathbb{O}}}$ can be written

19 $${W}_{{\mathbb{O}}}{({\mathcal{E}})}^{-1}=\mathop{{\rm{min}}}\limits_{{\mathcal{M}}\in {\mathbb{O}}}{R}_{{\rm{max}}}({J}_{{\mathcal{M}}}\parallel {J}_{{\mathcal{E}}})\qquad\qquad\qquad\qquad\qquad\qquad\;\,$$

20 $$=\mathop{{\rm{min}}}\limits_{{\mathcal{M}}\in {\mathbb{O}}}\mathop{{\rm{max}}}\limits_{\psi }{R}_{{\rm{max}}}({\rm{id}}\otimes {\mathcal{M}}(\psi )\parallel {\rm{id}}\otimes {\mathcal{E}}(\psi ))$$

21 $$=\mathop{{\rm{max}}}\limits_{\psi }\mathop{{\rm{min}}}\limits_{{\mathcal{M}}\in {\mathbb{O}}}{R}_{{\rm{max}}}({\rm{id}}\otimes {\mathcal{M}}(\psi )\parallel {\rm{id}}\otimes {\mathcal{E}}(\psi )).$$

The next step is to use convex duality to express the robustness and weight as

22 $${R}_{{\mathbb{O}}}({\mathcal{E}})= \, 	\mathop{{\rm{max}}}\limits_{\psi }\big\{{\rm{Tr}} \left(X\ {\rm{id}}\otimes {\mathcal{E}}(\psi )\right)\ \left|\ X \ge 0,\right.\\ 	{\rm{Tr}} \left(X\ {\rm{id}}\otimes {\mathcal{M}}(\psi )\right)\le 1\ \forall {\mathcal{M}}\in {\mathbb{O}}\big\},$$

23 $${W}_{{\mathbb{O}}}({\mathcal{E}})= \, 	\mathop{{\rm{min}}}\limits_{\psi }\big\{{\rm{Tr}} \left(X\ {\rm{id}}\otimes {\mathcal{E}}(\psi )\right)\ \left|\ X\ge 0,\right.\\ 	{\rm{Tr}} \left(X\ {\rm{id}}\otimes {\mathcal{M}}(\psi )\right)\ge 1\ \forall {\mathcal{M}}\in {\mathbb{O}}\big\}.$$

The core of the idea behind the proof of Theorem 1 is then as follows. Due to the purity of the target channel ${\mathcal{T}}$ (whether it is a unitary channel ${\mathcal{U}}$ or a replacement channel ${{\mathcal{R}}}_{\phi }$), the expression for the fidelity ${F}_{{\mathbb{O}}}$ simplifies: we either have

24 $${F}_{{\mathbb{O}}}({\mathcal{U}})=\mathop{{\rm{max}}}\limits_{{\mathcal{M}}\in {\mathbb{O}}}{\rm{Tr}}\left({\rm{id}}\otimes {\mathcal{U}}({\psi }^{\star })\ {\rm{id}}\otimes {\mathcal{M}}({\psi }^{\star })\right)$$

for some optimal pure state *ψ*^⋆^, or, in the state case, we can write

25 $${F}_{{\mathbb{F}}}(\phi )=\mathop{{\rm{max}}}\limits_{\sigma \in {\mathbb{F}}}{\rm{Tr}}(\phi \sigma ).$$

This allows us to use either the target channel ${\mathcal{U}}$ or the target state *ϕ* to construct feasible solutions for the dual form of ${R}_{{\mathbb{O}}}$ and ${W}_{{\mathbb{O}}}$. Specifically, the operator $\frac{1}{{F}_{{\mathbb{O}}}({\mathcal{U}})}\left({\rm{id}}\otimes {\mathcal{U}}({\psi }^{\star })\right)$ or $\frac{1}{{F}_{{\mathbb{F}}}(\phi )}\phi$ can be used to lower bound ${R}_{{\mathbb{O}}}$, while the operator $\frac{1}{1-{F}_{{\mathbb{O}}}({\mathcal{U}})}\left({\mathbb{1}}-{\rm{id}}\otimes {\mathcal{U}}({\psi }^{\star })\right)$ or $\frac{1}{1-{F}_{{\mathbb{F}}}(\phi )}\left({\mathbb{1}}-\phi \right)$ gives an upper bound on ${W}_{{\mathbb{O}}}$. These bounds immediately lead to the restrictions stated in Theorem 1.

### Many-copy bounds (Theorem 2)

Mathematically, an *n*-channel quantum process ϒ — the most general physically realisable manipulation protocol involving multiple quantum channels — is an *n*-linear map which takes *n* channels as input and outputs a single channel. Although the property of complete positivity is sometimes expected of such transformations^2,13^, we do not require it, and all of our results are valid as long as the maps in consideration satisfy ${{\Upsilon }}({{\mathcal{N}}}_{1},\ldots ,{{\mathcal{N}}}_{n})\in {\rm{CPTP}}$ for any ${{\mathcal{N}}}_{1},\ldots ,{{\mathcal{N}}}_{n}\in {\rm{CPTP}}$. We can then define the set of free quantum processes as those which always result in a free channel, provided that all inputs are free:

26 $${{\mathbb{S}}}_{(n)}:=\left\{{{\Upsilon }}| \ {{\Upsilon }}({{\mathcal{M}}}_{1},\ldots ,{{\mathcal{M}}}_{n})\in {\mathbb{O}}\ \ \forall {{\mathcal{M}}}_{1},\ldots ,{{\mathcal{M}}}_{n}\in {\mathbb{O}}\right\}.$$

In this sense, superchannels can be understood as (completely positive) processes acting on a single input.

Our main technical contribution is to show a very general type of sub- or super-multiplicativity that the robustness and weight measures obey. In particular, we show that, given any collection of *n* channels $({{\mathcal{E}}}_{1},\ldots ,{{\mathcal{E}}}_{n})$, it holds that

27 $${W}_{{\mathbb{O}}}\left({{\Upsilon }}({{\mathcal{E}}}_{1},\ldots ,{{\mathcal{E}}}_{n})\right)\ge \mathop{\prod}\limits_{i}{W}_{{\mathbb{O}}}({{\mathcal{E}}}_{i})$$

and

28 $${R}_{{\mathbb{O}}}\left({{\Upsilon }}({{\mathcal{E}}}_{1},\ldots ,{{\mathcal{E}}}_{n})\right)\le \mathop{\prod}\limits_{i}{R}_{{\mathbb{O}}}({{\mathcal{E}}}_{i})$$

for any free process ${{\Upsilon }}\in {{\mathbb{S}}}_{(n)}$. The basic idea behind the proof is to take an optimal channels ${{\mathcal{M}}}_{i}$ such that each ${{\mathcal{E}}}_{i}$ satisfies ${J}_{{{\mathcal{E}}}_{i}}\ge {\mu }_{i}{J}_{{{\mathcal{M}}}_{i}}$ in the case of ${W}_{{\mathbb{O}}}$ or ${J}_{{{\mathcal{E}}}_{i}}\le {\mu }_{i}{J}_{{{\mathcal{M}}}_{i}}$ in the case of ${R}_{{\mathbb{O}}}$. By showing that ${{\Upsilon }}({\mu }_{1}{{\mathcal{M}}}_{1},{\mu }_{2}{{\mathcal{M}}}_{2},\ldots ,{\mu }_{n}{{\mathcal{M}}}_{n})$ forms a valid feasible solution for ${W}_{{\mathbb{O}}}\left({{\Upsilon }}({{\mathcal{E}}}_{1},\ldots ,{{\mathcal{E}}}_{n})\right)$ or ${R}_{{\mathbb{O}}}\left({{\Upsilon }}({{\mathcal{E}}}_{1},\ldots ,{{\mathcal{E}}}_{n})\right)$, we obtain our desired result. Notably, the proof uses only the positivity and *n*-linearity of the free process ϒ, requiring no additional assumptions about the structure of the transformation.

Combined with Theorem 1, our result then immediately leads to the statement of Theorem 2. However, we stress that the property of sub- or super-multiplicativity that we have shown is much more general: the target in the transformation need not be a pure (unitary or replacement) channel, meaning that the inequalities in Eqs. (27)–(28) are valid for any channel manipulation protocol. Although in the main text we have focused on the application to the task of channel distillation, this general feature of the robustness and weight measures can find use in broader channel processing tasks that involve multiple channels.

For instance, the task of channel synthesis is concerned with simulating the action of the given channel ${\mathcal{E}}$ by employing multiple uses of another channel, ${\mathcal{F}}$, and processing them with a free transformation protocol ϒ. We then immediately obtain lower bounds on the required number of uses of ${\mathcal{F}}$ under any physical transformation protocol:

29 $$n\ge \frac{{\rm{log}}{R}_{{\mathbb{O}}}({\mathcal{E}})}{{\rm{log}}{R}_{{\mathbb{O}}}({\mathcal{F}})},\quad n\ge \frac{{\rm{log}}{W}_{{\mathbb{O}}}({\mathcal{E}})}{{\rm{log}}{W}_{{\mathbb{O}}}({\mathcal{F}})},$$

where in the second inequality we have assumed that ${W}_{{\mathbb{O}}}({\mathcal{E}})$ and ${W}_{{\mathbb{O}}}({\mathcal{F}})$ are not both 0. When ${\mathcal{F}}$ is chosen to be a pure resource channel such as the target ${\mathcal{T}}$, this can be understood as the opposite task to distillation — resource dilution.

### Asymptotic bounds (Theorem 3)

Both of our asymptotic bound in Theorem 3 are consequences of the results of Theorem 1 and 2 coupled with the assumption that ${F}_{{\mathbb{O}}}({{\mathcal{T}}}^{\otimes m})={F}_{{\mathbb{O}}}{({\mathcal{T}})}^{m}$. This means in particular that they apply to general manipulation protocols ϒ without making assumptions about their structure, in contrast to most previous asymptotic bounds in the literature which explicitly considered sequential manipulation protocols with a fixed causal order.

We note that the second, regularised bound for parallel channel transformations (Eq. (12)) requires a more careful approach, relying also on some technical bounds on the fidelity distance between channels. In particular, the ‘smoothing’ parameter *δ* encountered here is the reason why the result applies to parallel manipulation protocols only — an extension to more general transformations would entail an optimisation in the space of quantum combs (or quantum processes), and a straightforward application of our methods to this case does not appear to be possible. Whether this can be circumvented with a different approach remains an open question.

### Extension to probabilistic protocols

We have so far focused our discussion on deterministic channel transformations where superchannels (and quantum processes) transform channels to channels. To investigate a probabilistic version of such protocols, we need to consider ‘sub-superchannels’: the linear maps which transform quantum channels to probabilistic implementations of channels in the form of completely positive, trace–non-increasing maps (subchannels), even when acting only on a part of a larger system^60^. The operational meaning of these maps becomes clear by considering them as constituents of superinstruments, i.e., collections of sub-superchannels $\{{\tilde{{{\Theta }}}}_{i}\}$ each representing a single outcome of a probabilistic transformation such that the overall transformation ${\sum }_{i}{\tilde{{{\Theta }}}}_{i}$ is a superchannel. Just as the usual quantum instrument, a superinstrument can be assumed to come with a classical register recording which sub-superchannel was applied. Then, probabilistic protocols are declared successful when we learn that ${\tilde{{{\Theta }}}}_{0}$ was realised and are judged to have failed otherwise. To introduce the notion of free transformation in this context, let us first define the set of free subchannels. If we think of free subchannels as a probabilistic version of free channels, it is natural to impose that every free subchannel probabilistically realises a transformation implemented by some free channel. This observation motivates us to define the set of free subchannels $\tilde{{\mathbb{O}}}$ with respect to the given set of free channels ${\mathbb{O}}$ as

30 $$\tilde{{\mathbb{O}}}:=	\big\{\tilde{{\mathcal{M}}} | \forall \rho \in {\mathbb{D}},\ \exists \ {\mathcal{M}}\in {\mathbb{O}},\ t\in [0,1]\\ 	{\rm{s.t.}}\ \ {\rm{id}} \otimes \tilde{{\mathcal{M}}}(\rho )=t\cdot {\rm{id}}\otimes {\mathcal{M}}(\rho )\big\},$$

and we correspondingly define the set of free sub-superchannels as $\tilde{{\mathbb{S}}}:=\left\{\tilde{{{\Theta }}}| \forall {\mathcal{M}}\in {\mathbb{O}},\ \tilde{{{\Theta }}}({\mathcal{M}})\in \tilde{{\mathbb{O}}}\right\}$.

We also need to establish a figure of merit for the probabilistic purification protocol. A subtlety is that the probability of the occurrence of a sub-superchannel $\tilde{{{\Theta }}}$ depends not only on the input channel ${\mathcal{E}}$, but also on the input state *ψ* as ${\rm{Tr}}[{\rm{id}}\otimes \tilde{{{\Theta }}}({\mathcal{E}})(\psi )]$. Integrating this observation with the definition of the fidelity for channels $F({\mathcal{E}},{\mathcal{T}})$, we define the fidelity between the target channel and an output subchannel conditioned on its occurrence as

31 $${F}_{{\rm{cond}}}(\tilde{{{\Theta }}}({\mathcal{E}}),{\mathcal{T}}):=\mathop{{\rm{min}}}\limits_{\psi }F\left(\frac{{\rm{id}}\otimes \tilde{{{\Theta }}}({\mathcal{E}})(\psi )}{p(\psi )},{\rm{id}}\otimes {\mathcal{T}}(\psi )\right)$$

where $$p(\psi )={\rm{Tr}}[{\rm{id}}\otimes \tilde{{{\Theta }}}({\mathcal{E}})(\psi )]$$.

We can then establish an analogue of Theorem 1 for probabilistic channel manipulation. Specifically, we show that if there exists a free sub-superchannel $\tilde{{{\Theta }}}\in \tilde{{\mathbb{S}}}$ which achieves the transformation ${\mathcal{E}}\to {\mathcal{T}}$ with fidelity ${F}_{{\rm{cond}}}(\tilde{{{\Theta }}}({\mathcal{E}}),{\mathcal{T}})\ge 1-\varepsilon$ and probability $p={\rm{Tr}}[{\rm{id}}\otimes \tilde{{{\Theta }}}({\mathcal{E}})(\psi )]$, then

32 $$\varepsilon \ge 1-\frac{{R}_{{\mathbb{O}}}({\mathcal{E}})\ {F}_{{\mathbb{O}}}^{\psi }({\mathcal{U}})}{p}$$

and

33 $$\varepsilon \ge 1-\frac{1-(1-{F}_{{\mathbb{O}}}^{\psi }({\mathcal{U}})){W}_{{\mathbb{O}}}({\mathcal{E}})}{p}$$

where ${F}_{{\mathbb{O}}}^{\psi }({\mathcal{U}}):={{\rm{max}}}_{{\mathcal{M}}\in {\mathbb{O}}}F({\rm{id}}\otimes {\mathcal{U}}(\psi ),{\rm{id}}\otimes {\mathcal{M}}(\psi ))$. This resembles our previous bounds, but now explicitly incorporates the dependence on a probability *p*.

Another type of bound for probabilistic transformations can be obtained by taking ${\mathcal{M}}\in {\mathbb{O}}$ to be a free channel such that ${J}_{{\mathcal{E}}}\ge {W}_{{\mathbb{O}}}({\mathcal{E}}){J}_{{\mathcal{M}}}$. We then obtain

34 $$\varepsilon \ \ge \ (1-{F}_{{\mathbb{O}}}^{\psi }({\mathcal{U}}))\ \frac{{W}_{{\mathbb{O}}}({\mathcal{E}}){\rm{Tr}}[\left({\rm{id}}\right.\otimes \tilde{{{\Theta }}}({\mathcal{M}})(\psi )]}{p}.$$

This bound addresses the question of whether the no-go statement implied by Theorem 1, which says that perfect purification with *ε* = 0 is impossible for any channel with ${W}_{{\mathbb{O}}}({\mathcal{E}})\, > \, 0$, remains valid in probabilistic cases. Eq. (34) implies that if ${\rm{Tr}}[{\rm{id}}\otimes \tilde{{{\Theta }}}({\mathcal{M}})(\psi )]\, > \, 0$, the no-go theorem still holds. On the other hand, if ${\rm{Tr}}[{\rm{id}}\otimes \tilde{{{\Theta }}}({\mathcal{M}})(\psi )]=0$, meaning that the free part of ${\mathcal{E}}$ is completely cut off by the selective operation $\tilde{{{\Theta }}}$, then this does not give us any insight into *ε*. This is actually a natural consequence because such a perfect purification is indeed possible, as we discuss in Supplementary Note 5 in detail.

## Supplementary information

Supplementary Information

## Data Availability

No data sets were generated during this study.

## Code availability

Source codes used to generate the plots are available from the corresponding author upon request.
