# Supplementary material for: Fundamental limitations on distillation of quantum channel resources
Source: Nat Commun. 2021 Jul 20;12:4411. doi: 10.1038/s41467-021-24699-0 (PMC8292459; doi:10.1038/s41467-021-24699-0)
Supplement: Supplementary file 1 — Supplementary Information [file 41467_2021_24699_MOESM1_ESM.pdf]

Fundamental limitations on distillation of quantum channel resources
— Supplementary Information —

Bartosz Regula[1,*] and Ryuji Takagi[1,2,†]

[1]School of Physical and Mathematical Sciences, Nanyang Technological University, 637371, Singapore
[2]Center for Theoretical Physics and Department of Physics,
Massachusetts Institute of Technology, Cambridge, Massachusetts 02139, USA

The Supplementary Information is structured as follows. In Supplementary Note 1, we introduce in full technical detail our notation and the general setting of channel manipulation and quantum resources, as well as establish some auxiliary results which will help us in characterising the robustness and weight monotones. In Supplementary Note 2, we prove our general no-go theorems on the performance of channel distillation protocols (Theorem 1 in main text). We extend these insights in Supplementary Note 3, where we study many-copy channel manipulation protocols, use our results to provide bounds on the distillation overhead (Theorem 2 in main text), establish bounds applicable beyond distillation protocols, and apply our main findings to the asymptotic setting (Theorem 3 in main text). We discuss explicit applications in Supplementary Note 4 with a detailed discussion of how our bounds can shed light on important tasks in quantum communication and fault-tolerant quantum computation. We conclude in Supplementary Note 5 with a discussion of probabilistic distillation protocols and an extension of our approach to this setting. The Supplementary Information may, in some places, overlap with the content of the main text in order to be as self-contained as possible and to provide additional details and motivations.

Contents

1. Setting 1
   1.1. Resource theories 2
   1.2. Benchmarking channel transformations and resources 2
   1.3. Properties of the robustness and resource weight 3
2. No-go theorems for quantum channel distillation 5
   2.1. Unitary channel distillation 5
   2.2. State-based resources 7
3. Many-copy transformations and protocols beyond distillation 10
   3.1. Bounds for many-copy manipulation 10
   3.2. Asymptotic rates of distillation 12
4. Applications 15
   4.1. Quantum communication 15
   4.2. Quantum gate synthesis and magic states 19
   4.3. Proof of Lem. 18 and extension to other resources 21
5. Extension to probabilistic protocols 22

Supplementary Note 1: Setting

All of our discussions will take place in finite-dimensional Hilbert spaces. Given the Hilbert space of a system A, we will write $\mathbb{L}(A)$ for the linear operators, and $\mathbb{D}(A)$ for the density operators acting on this space. We use $\mathrm{CPTP}(A \to B)$ to denote the set of quantum channels, i.e. completely positive and trace-preserving (CPTP) maps from $\mathbb{L}(A)$ to $\mathbb{L}(B)$. We associate with each channel $\mathcal{E} : A \to B$ its Choi matrix $J_{\mathcal{E}} := \mathrm{id} \otimes \mathcal{E}(\Phi^+) \in \mathbb{L}(RB)$, where $\Phi^+ = \sum_{i,j} |ii\rangle\langle jj|$ is the unnormalised maximally entangled state in $\mathbb{L}(RA)$ and $R \cong A$. The normalised Choi state is then $\widetilde{J}_{\mathcal{E}} := J_{\mathcal{E}}/d_A$. We use $\langle A, B\rangle = \mathrm{Tr}(A^\dagger B)$ for the Hilbert-Schmidt inner product between operators. All logarithms will be taken to the base 2, unless specified otherwise.

We will be concerned with schemes for transformations of quantum channels, that is, maps $\mathrm{CPTP}(A \to B) \to \mathrm{CPTP}(C \to D)$. Recall that such superchannels [1] can be written as $\Theta(\mathcal{E}) = \mathcal{M}_{RB\to D} \circ (\mathrm{id}_R \otimes \mathcal{E}) \circ \mathcal{N}_{C\to RA}$ where $\mathcal{N}, \mathcal{M}$ are some pre- and post-processing quantum channels, id is the identity channel, and R denotes an ancillary system (see Fig. 1 in the main text).

* bartosz.regula@gmail.com
† ryuji.takagi@ntu.edu.sg

1.1. Resource theories

As discussed in the main text, a given set of channels $\mathbb{O}$ is designated as *free* — these are the channels which are freely available to use within the constraints of the given physical setting, and all channels outside of this set are the resourceful ones. To ensure broad applicability, we only make the natural assumptions that the set $\mathbb{O}$ is closed and convex. When discussing channels acting on different spaces, we will assume that each space in consideration has its own associated set of free channels $\mathbb{O}$, and for simplicity of notation we often do not explicitly indicate the relevant spaces.

The transformations of channels, and the issue of what exactly constitutes a free channel transformation, are approached in various ways [2–5]. Often, one is interested in the manipulation of channels under superchannels of the form $\Theta(\mathcal{E}) = \mathcal{M} \circ (\mathrm{id} \otimes \mathcal{E}) \circ \mathcal{N}$ where the pre- and post-processing channels $\mathcal{N}$ and $\mathcal{M}$ are both free. In order to apply our results to more general settings, we will make no such assumption, and instead take the weakest possible constraint on the considered set of free superchannels: that for any $\mathcal{M} \in \mathbb{O}$, we necessarily have $\Theta(\mathcal{M}) \in \mathbb{O}$. We use $\mathbb{S}$ to denote the set of all such resource-preserving superchannels. By studying these transformations, we will therefore obtain the most general bounds on the achievable performance of *any* free channel manipulation protocol, since any valid choice of free transformations will necessarily be a subset of $\mathbb{S}$.

The first type of resources that we consider is concerned with the investigation of intrinsic channel resources; this includes the various resource theories of quantum communication [4, 6–9], the related setting of quantum memories [10, 11], and quantum error mitigation [12, 13]. The other type is concerned with an underlying state-based resource and the manipulation of channels in order to extract or utilise the state resource more effectively; this includes, for instance, quantum entanglement [14–19], coherence [20–24], thermodynamics [25], or non-stabiliser (magic) states [26, 27]. In the case of such state-based theories, we will use $\mathbb{F}$ to denote the corresponding (convex and closed) set of free states. We will then study three levels of resources: the free states $\mathbb{F}$, for example separable states in entanglement theory; the free channels $\mathbb{O}$, for example the closure of the set of local operations and classical communication (LOCC) or other chosen classes of operations in entanglement manipulation; and $\mathbb{S}$, which we will always take to be the set of all superchannels that preserve the chosen set $\mathbb{O}$, and which can be understood as the most general way to manipulate free channels while preserving their freeness.

We remark that although we focus here on the framework accounting for all channels as valid objects under study, it is sometimes useful to impose certain constraints on the considered channels (both free and non-free), restricting the attention to CPTP maps possessing a certain structure. Such an approach can be used to investigate settings such as Bell nonlocality [28–33], post-quantum correlations [34–37], and quantum contextuality [38–41]. Our results can indeed be extended to those cases, which we discuss in more detail elsewhere [42].

1.2. Benchmarking channel transformations and resources

In its general formulation, the task of distillation can be understood as the transformation of a noisy resource channel $\mathcal{E} : A \to B$ into 'pure' or 'perfect' resources, which are represented by some target channel $\mathcal{T} : C \to D$. Our task then is to understand when one can achieve transformations of the type $\Theta(\mathcal{E}) = \mathcal{T}$, with $\Theta \in \mathbb{S}$ being a free transformation.

However, in any practical setting, it is important to allow for the possibility of error in the transformation, reflecting the physical imperfections in the manipulation of channels. To account for this, we will employ the worst-case fidelity between two channels $\mathcal{E}, \mathcal{F} : A \to B$ [43, 44][1]

$$\begin{aligned} F(\mathcal{E},\mathcal{F}) &:= \min_{\rho_{RA}} F(\mathrm{id}\otimes\mathcal{E}(\rho), \mathrm{id}\otimes\mathcal{F}(\rho)) \\ &= \min_{\psi_{RA}} F(\mathrm{id}\otimes\mathcal{E}(\psi), \mathrm{id}\otimes\mathcal{F}(\psi)), \end{aligned} \tag{1}$$

where $F(\rho,\sigma) = \left\|\sqrt{\rho}\sqrt{\sigma}\right\|_1^2$ is the state fidelity, and in the second line the optimisation is constrained to pure input states. We will thus aim to achieve a transformation such that $F(\Theta(\mathcal{E}),\mathcal{T}) \geq 1-\varepsilon$ for some small error ε. This fidelity metric is very closely related to the channel diamond norm $\|\cdot\|_\diamond$ [45] as $1-\sqrt{F(\mathcal{E},\mathcal{F})} \leq \frac{1}{2}\|\mathcal{E}-\mathcal{F}\|_\diamond \leq \sqrt{1-F(\mathcal{E},\mathcal{F})}$ [43, 46], meaning that our results can be straightforwardly adapted also to cases when diamond norm error is considered.

In order to quantify the resources of general quantum channels, we will employ two different resource measures which were first defined in resource theories of states. These quantities are also directly related to more general geometric concepts based on Hilbert's projective metric [47, 48], and specifically the Funk metric [49]. Let us begin by recalling their definitions in the state-based setting[2]. First, the (generalised) *robustness* [50, 51] is given by

$$R_{\mathbb{F}}(\rho) := \min\left\{\lambda \;\middle|\; \rho \leq \lambda\sigma,\ \sigma\in\mathbb{F}\right\}, \tag{2}$$

[1] The optimisation in the definition of $F(\mathcal{E},\mathcal{F})$ can be understood as being over arbitrary ancillary spaces R, but in fact it suffices to take $R \cong A$ [43, 44].
[2] We note that our notational conventions are slightly different from ones typically used in the literature; in particular, the robustness is often defined as $R_{\mathbb{F}}(\rho) - 1$ in our notation, while the resource weight is often defined $1 - W_{\mathbb{F}}(\rho)$

where the inequality is with respect to the cone of positive semidefinite operators. This can be understood as the least amount λ such that, when mixed with another state ω, the mixture $\rho + (\lambda - 1)\omega = \lambda\sigma$ has $\sigma \in \mathbb{F}$. The measure previously found a number of applications in different operational tasks [51–61]. In order to avoid pathological cases, throughout this work we assume that the robustness $R_{\mathbb{F}}(\rho)$ is finite (that is, the minimum in Eq. (2) exists) — this can be guaranteed for any state ρ by requiring that the set of free states $\mathbb{F}$ contains at least one state of full rank, which is a natural requirement in physical theories of interest.

A closely related quantity is the *resource weight*:

$$W_{\mathbb{F}}(\rho) := \max \left\{ \lambda \;\middle|\; \rho \geq \lambda\sigma,\; \sigma \in \mathbb{F} \right\}. \tag{3}$$

The resource weight generalises a measure of entanglement known as the best separable approximation [62], and was recently shown to be a meaningful quantifier of general resources [63–65]. The optimal λ here can be understood the largest weight that a free state σ can take in a convex decomposition $\rho = \lambda\sigma + (1 - \lambda)\omega$ for an arbitrary state ω. This monotone is a peculiar quantity: although it gives a faithful and strongly monotonic measure in any convex resource theory [66], it exhibits unusual behaviour. Specifically, if there exists a free state $\sigma \in \operatorname{supp}(\rho)$, we necessarily have $W_{\mathbb{F}}(\rho) > 0$; conversely, if a given ρ does not have any free states in its support, the weight achieves the minimum value $W_{\mathbb{F}}(\rho) = 0$. In particular, any resourceful pure state $\psi \notin \mathbb{F}$ has $W_{\mathbb{F}}(\psi) = 0$.

The measures can be extended to quantum channels [4, 21, 57, 63, 67] by considering the corresponding Choi matrices:

$$\begin{aligned} R_{\mathbb{O}}(\mathcal{E}) &:= \min \left\{ \lambda \;\middle|\; J_{\mathcal{E}} \leq \lambda J_{\mathcal{M}},\; \mathcal{M} \in \mathbb{O} \right\}, \\ W_{\mathbb{O}}(\mathcal{E}) &:= \max \left\{ \lambda \;\middle|\; J_{\mathcal{E}} \geq \lambda J_{\mathcal{M}},\; \mathcal{M} \in \mathbb{O} \right\}. \end{aligned} \tag{4}$$

This can be equivalently understood as the optimisation over free channels $\mathcal{M}$ such that $\lambda\mathcal{M} - \mathcal{E}$ (in the case of the robustness) or $\mathcal{E} - \lambda\mathcal{M}$ (for the resource weight) is a completely positive map. Both of the quantities are valid resource monotones in that they satisfy monotonicity under any free superchannel $\Theta \in \mathbb{S}$, i.e. $R_{\mathbb{O}}(\Theta(\mathcal{E})) \leq R_{\mathbb{O}}(\mathcal{E})$, with the resource weight obeying a reverse monotonicity: $W_{\mathbb{O}}(\Theta(\mathcal{E})) \geq W_{\mathbb{O}}(\mathcal{E})$. Other useful properties of the measures, such as their submultiplicativity, can also be established (see Supplementary Notes 1.3 and 3). The quantities have a simple structure which allows for an efficient computation as a semidefinite program (SDP) whenever the set of channels $\mathbb{O}$ can be characterised using semidefinite constraints, which we will see to be the case in many relevant settings.

We will use one additional resource monotone in order to quantify how close a target channel $\mathcal{T}$ is to a free channel, and thus characterise how difficult it is to purify noisy channels into the target $\mathcal{T}$. We thus define the fidelity-based overlap measure

$$F_{\mathbb{O}}(\mathcal{T}) := \max_{\mathcal{M} \in \mathbb{O}} F(\mathcal{T}, \mathcal{M}). \tag{5}$$

In state-based theories, one analogously has

$$F_{\mathbb{F}}(\rho) := \max_{\sigma \in \mathbb{F}} F(\rho, \sigma). \tag{6}$$

1.3. Properties of the robustness and resource weight

We will use the fact that both the robustness and weight measures can be expressed in terms of the max-relative entropy $D_{\max}(\rho\|\sigma) := \log\inf \left\{ \lambda \;\middle|\; \rho \leq \lambda\sigma \right\}$ [68], whose generalisation to channels obeys some useful properties [67]. In particular, defining $R_{\max}(\rho\|\sigma) := 2^{D_{\max}(\rho\|\sigma)}$, for any channels $\mathcal{E}, \mathcal{F} : A \to B$ one can define the optimised channel divergence [67]

$$R_{\max}(\mathcal{E}\|\mathcal{F}) := \max_{\psi_{RA}} R_{\max}(\mathrm{id} \otimes \mathcal{E}(\psi)\|\mathrm{id} \otimes \mathcal{F}(\psi)), \tag{7}$$

where $R \cong A$ and the maximisation can be regarded as being over the convex and compact set of density operators on R, with ψ_{RA} thought of as a purification of a given ρ_R. Crucially, it holds that [67, Lemma 12]

$$R_{\max}(\mathcal{E}\|\mathcal{F}) = R_{\max}(J_{\mathcal{E}}\|J_{\mathcal{F}}), \tag{8}$$

that is, it suffices to consider the Choi matrices of the channels to evaluate the max-relative entropy.

We can then write the robustness measure as

$$\begin{aligned} R_{\mathbb{O}}(\mathcal{E}) &= \min_{\mathcal{M} \in \mathbb{O}} R_{\max}(J_{\mathcal{E}}\|J_{\mathcal{M}}) \\ &= \min_{\mathcal{M} \in \mathbb{O}} \max_{\psi} R_{\max}(\mathrm{id} \otimes \mathcal{E}(\psi)\|\mathrm{id} \otimes \mathcal{M}(\psi)). \end{aligned} \tag{9}$$

In a very similar way, we notice that

$$W_{\mathbb{O}}(\mathcal{E})^{-1} = \min_{\mathcal{M}\in\mathbb{O}} R_{\max}(J_{\mathcal{M}} \| J_{\mathcal{E}}) = \min_{\mathcal{M}\in\mathbb{O}} \max_{\psi} R_{\max}(\mathrm{id}\otimes\mathcal{M}(\psi)\|\mathrm{id}\otimes\mathcal{E}(\psi)). \tag{10}$$

We will use the facts that the minimisation and maximisation problems in the above can be interchanged, which can be shown through the application of Sion's minimax theorem [69] similarly to how it was done in Refs. [5, 27].

Lemma 4. *We have that*

$$\begin{aligned} R_{\mathbb{O}}(\mathcal{E}) &= \max_{\psi} \min_{\mathcal{M}\in\mathbb{O}} R_{\max}(\mathrm{id}\otimes\mathcal{E}(\psi)\|\mathrm{id}\otimes\mathcal{M}(\psi)),\\ W_{\mathbb{O}}(\mathcal{E}) &= \min_{\psi} \max_{\mathcal{M}\in\mathbb{O}} R_{\max}(\mathrm{id}\otimes\mathcal{M}(\psi)\|\mathrm{id}\otimes\mathcal{E}(\psi))^{-1},\\ F_{\mathbb{O}}(\mathcal{E}) &= \min_{\psi} \max_{\mathcal{M}\in\mathbb{O}} F(\mathrm{id}\otimes\mathcal{E}(\psi), \mathrm{id}\otimes\mathcal{M}(\psi)). \end{aligned} \tag{11}$$

Proof. The minimax optimisation can be regarded as being over two convex, compact sets: the given set of free channels $\mathbb{O}$ and the set of density matrices on ρ_R, with the state ψ_{RA} taken to be a purification of ρ_R. To apply Sion's minimax theorem to $R_{\mathbb{O}}$, we then need that the objective function is quasi-concave in ρ_R and quasi-convex in $\mathcal{M}$ [69]. Since $R_{\max}$ is the composition of $D_{\max}$ with the non-decreasing function 2^x, quasi-convexity in $\mathcal{M}$ follows from the quasi-convexity of $D_{\max}$ [68], and quasi-concavity in ρ_R follows from the concavity of $D_{\max}$ (see [27, Prop. 13] and [5, Thm. 2]). The case of $W_{\mathbb{O}}$ follows similarly. The fidelity $F(\cdot,\cdot)$ is known to be concave in $\mathcal{M}$ [43] and can be shown to be convex in ρ_R following Refs. [5, 27]. ■

We proceed to establish other useful properties of the two measures.

Lemma 5. *The robustness and weight measures can be expressed in their dual forms as*

$$\begin{aligned} R_{\mathbb{O}}(\mathcal{E}) &= \max\left\{ \langle X, J_{\mathcal{E}}\rangle \;\middle|\; X \geq 0,\ \langle X, J_{\mathcal{M}}\rangle \leq 1\ \forall \mathcal{M}\in\mathbb{O} \right\}\\ &= \max_{\psi}\left\{ \langle X, \mathrm{id}\otimes\mathcal{E}(\psi)\rangle \;\middle|\; X \geq 0,\ \langle X, \mathrm{id}\otimes\mathcal{M}(\psi)\rangle \leq 1\ \forall \mathcal{M}\in\mathbb{O} \right\}, \end{aligned} \tag{12}$$

$$\begin{aligned} W_{\mathbb{O}}(\mathcal{E}) &= \min\left\{ \langle X, J_{\mathcal{E}}\rangle \;\middle|\; X \geq 0,\ \langle X, J_{\mathcal{M}}\rangle \geq 1\ \forall \mathcal{M}\in\mathbb{O} \right\}\\ &= \min_{\psi}\left\{ \langle X, \mathrm{id}\otimes\mathcal{E}(\psi)\rangle \;\middle|\; X \geq 0,\ \langle X, \mathrm{id}\otimes\mathcal{M}(\psi)\rangle \geq 1\ \forall \mathcal{M}\in\mathbb{O} \right\}, \end{aligned} \tag{13}$$

$$R_{\mathbb{F}}(\rho) = \max\left\{ \langle X, \rho\rangle \;\middle|\; X \geq 0,\ \langle X, \sigma\rangle \leq 1\ \forall \sigma\in\mathbb{F} \right\}, \tag{14}$$

$$W_{\mathbb{F}}(\rho) = \min\left\{ \langle X, \rho\rangle \;\middle|\; X \geq 0,\ \langle X, \sigma\rangle \geq 1\ \forall \sigma\in\mathbb{F} \right\}. \tag{15}$$

Proof. Follows from standard convex duality results (see e.g. [57, 70]) and Lem. 4. ■

Lemma 6. *For any superchannel $\Theta \in \mathbb{S}$, it holds that*

$$\begin{aligned} R_{\mathbb{O}}(\Theta(\mathcal{E})) &\leq R_{\mathbb{O}}(\mathcal{E}),\\ W_{\mathbb{O}}(\Theta(\mathcal{E})) &\geq W_{\mathbb{O}}(\mathcal{E}). \end{aligned} \tag{16}$$

Proof. Let $\mathcal{M} \in \mathbb{O}$ be any channel such that $J_{\mathcal{E}} \leq \lambda J_{\mathcal{M}}$. By definition of the set $\mathbb{S}$, we have that $\Theta(\mathcal{M}) = \mathcal{M}' \in \mathbb{O}$. Since Θ preserves positivity, it holds that $J_{\Theta(\mathcal{E})} \leq \lambda J_{\Theta(\mathcal{M})} = \lambda J_{\mathcal{M}'}$, so $\mathcal{M}'$ is a valid feasible solution for $R_{\mathbb{O}}(\Theta(\mathcal{E}))$, which concludes the proof. The case of $W_{\mathbb{O}}$ follows analogously. ■

The result below applies to state-based resource theories, and shows an exact relation between the channel-based and state-based measures.

Lemma 7. *For any replacement channel $\mathcal{R}_\omega : A \to B$ defined as $\mathcal{R}_\omega(\cdot) = \mathrm{Tr}(\cdot)\omega$ with some fixed ω, it holds that*

$$\begin{aligned} R_{\mathbb{O}}(\mathcal{R}_\omega) &\geq R_{\mathbb{F}}(\omega),\\ W_{\mathbb{O}}(\mathcal{R}_\omega) &\leq W_{\mathbb{F}}(\omega),\\ F_{\mathbb{O}}(\mathcal{R}_\omega) &\leq F_{\mathbb{F}}(\omega). \end{aligned} \tag{17}$$

If the class of operations $\mathbb{O}$ contains all replacement channels $\mathcal{R}_\sigma : A \to B$ with $\sigma \in \mathbb{F}$, then equality holds in all of the above.

Proof. Taking any feasible dual solution X for $R_{\mathbb{F}}(\omega)$ in Eq. (12) and any $\tau \in \mathbb{F}$, we can see that the operator $\tau^T \otimes X$ is feasible for $R_{\mathbb{O}}$ since $\left\langle \tau^T \otimes X, J_{\mathcal{M}} \right\rangle = \langle X, \mathcal{M}(\tau) \rangle \leq 1$ for any $\mathcal{M} \in \mathbb{O}$ using the Choi-Jamiołkowski isomorphism. This immediately gives that

$$\begin{aligned} &R_{\mathbb{O}}(\mathcal{R}_\omega)\\ &\quad \geq \max \left\{ \left\langle \tau^T \otimes X, J_{\mathcal{R}_\omega} \right\rangle \;\middle|\; \langle X, \sigma \rangle \leq 1 \; \forall \sigma \in \mathbb{F},\; X \geq 0 \right\}\\ &\quad = \max \left\{ \langle X, \omega \rangle \;\middle|\; \langle X, \sigma \rangle \leq 1 \; \forall \sigma \in \mathbb{F},\; X \geq 0 \right\}\\ &\quad = R_{\mathbb{F}}(\omega). \end{aligned} \tag{18}$$

Now, assume that $\mathcal{R}_\sigma \in \mathbb{O} \; \forall \sigma \in \mathbb{F}$. From the definition of the robustness, we know that there exists a $\tau \in \mathbb{F}$ such that $\omega \leq R_{\mathbb{F}}(\omega)\,\tau$. But then $\mathbb{1} \otimes \omega \leq \mathbb{1} \otimes [R_{\mathbb{F}}(\omega)\,\tau] = R_{\mathbb{F}}(\omega)\, J_{\mathcal{R}_\tau}$. Since $\mathcal{R}_\tau \in \mathbb{O}$, this gives that $R_{\mathbb{O}}(\mathcal{R}_\omega) \leq R_{\mathbb{F}}(\omega)$.

The case of $W_{\mathbb{O}}$ proceeds analogously.

For the fidelity, consider that

$$F_{\mathbb{O}}(\mathcal{R}_\omega) = \min_{\rho_{RA}} \max_{\mathcal{M} \in \mathbb{O}} F(\mathrm{id} \otimes \mathcal{M}(\rho), \mathrm{id} \otimes \mathcal{R}_\omega(\rho)). \tag{19}$$

Taking the ansatz $\rho \otimes \tau$ for the input state, where $\rho \in \mathbb{D}(R)$ is an arbitrary state and $\tau \in \mathbb{F}$, we get

$$\begin{aligned} F_{\mathbb{O}}(\mathcal{R}_\omega) &\leq \max_{\mathcal{M} \in \mathbb{O}} F(\rho \otimes \mathcal{M}(\tau), \rho \otimes \omega)\\ &= \max_{\mathcal{M} \in \mathbb{O}} F(\mathcal{M}(\tau), \omega)\\ &\leq \max_{\sigma \in \mathbb{F}} F(\sigma, \omega)\\ &= F_{\mathbb{F}}(\omega), \end{aligned} \tag{20}$$

where the second line follows from the data processing inequality for the fidelity, and the third line since $\mathcal{M}(\tau) \in \mathbb{F}$ for any $\tau \in \mathbb{F}$. For the converse inequality, assuming that any $\mathcal{R}_\sigma$ is in $\mathbb{O}$ gives

$$\begin{aligned} F_{\mathbb{O}}(\mathcal{R}_\omega) &\geq \max_{\sigma \in \mathbb{F}} \min_{\rho_{RA}} F(\mathrm{id} \otimes \mathcal{R}_\sigma(\rho), \mathrm{id} \otimes \mathcal{R}_\omega(\rho))\\ &= \max_{\sigma \in \mathbb{F}} \min_{\rho_{RA}} F([\mathrm{Tr}_A\, \rho] \otimes \sigma, [\mathrm{Tr}_A\, \rho] \otimes \omega)\\ &= \max_{\sigma \in \mathbb{F}} F(\sigma, \omega)\\ &= F_{\mathbb{F}}(\omega). \end{aligned} \tag{21}$$

■

Supplementary Note 2: No-go theorems for quantum channel distillation

The results of this section establish Theorem 1 of the main text, explicitly divided into two cases depending on whether the target channel is a unitary or replacement channel.

2.1. Unitary channel distillation

Within the context of channel-based resource theories, it is natural to regard some unitary channel $\mathcal{U}(\cdot) = U \cdot U^\dagger$ as the target of distillation protocols. Notice that in this case the measure $F_{\mathbb{O}}$ simplifies to

$$F_{\mathbb{O}}(\mathcal{U}) = \max_{\mathcal{M} \in \mathbb{O}} \min_{\psi_{RC}} \langle \mathrm{id} \otimes \mathcal{U}(\psi), \mathrm{id} \otimes \mathcal{M}(\psi) \rangle \tag{22}$$

since $\mathrm{id} \otimes \mathcal{U}(\psi)$ is a pure state. We also recall that, in cases where this distance might not be easy to compute, one can instead choose to employ the more straightforward fidelity measure which uses the corresponding (normalised) Choi matrices:

$$\widetilde{F}_{\mathbb{O}}(\mathcal{U}) := \max_{\mathcal{M}\in\mathbb{O}} F\left(\widetilde{J}_{\mathcal{U}}, \widetilde{J}_{\mathcal{M}}\right) = \max_{\mathcal{M}\in\mathbb{O}} \left\langle \widetilde{J}_{\mathcal{U}}, \widetilde{J}_{\mathcal{M}} \right\rangle. \tag{23}$$

Indeed, this quantity is the figure of merit in many communication scenarios [71–73], and can be alternatively expressed as the fidelity averaged over all input states [44, 74].

As our first main result, we then establish a general bound on the error necessarily incurred in any transformation of a channel under free superchannels.

Theorem 1(a). *If there exists a free superchannel $\Theta \in \mathbb{S}$ such that $F(\Theta(\mathcal{E}), \mathcal{U}) \geq 1 - \varepsilon$ for some resourceful unitary channel $\mathcal{U}$, then*

$$\varepsilon \geq 1 - F_{\mathbb{O}}(\mathcal{U})\, R_{\mathbb{O}}(\mathcal{E}) \tag{24}$$

and

$$\varepsilon \geq [1 - F_{\mathbb{O}}(\mathcal{U})]\, W_{\mathbb{O}}(\mathcal{E}). \tag{25}$$

Notice from the weight-based bound in Eq. (25) that, when $\varepsilon = 0$, the transformation $\mathcal{E} \to \mathcal{U}$ is impossible for any channel with $W_{\mathbb{O}}(\mathcal{E}) > 0$. Recalling that $W_{\mathbb{O}}(\mathcal{E})$ is 0 if and only if $J_{\mathcal{E}}$ has no free Choi matrices $J_{\mathcal{M}}$ with $\mathcal{M} \in \mathbb{O}$ in its support, we conclude that zero-error distillation is impossible whenever the given channel has any free channels in $\mathrm{supp}(J_{\mathcal{E}})$. This recovers a no-go result of Ref. [75] and extends insights from quantum state distillation [59, 75].

For clarity, we will divide the proof of Theorem 1(a) into two parts and consider each bound separately. We begin with the robustness $R_{\mathbb{O}}$.

Proposition 8. *If there exists a free supermap $\Theta \in \mathbb{S}$ such that $\Theta(\mathcal{E}) = \mathcal{N}$ for some channel $\mathcal{N}$ with $F(\mathcal{N}, \mathcal{U}) \geq 1 - \varepsilon$, then*

$$\varepsilon \geq 1 - R_{\mathbb{O}}(\mathcal{E})\, F_{\mathbb{O}}(\mathcal{U}). \tag{26}$$

Proof. Recall from Lem. 5 that for any channel we have

$$R_{\mathbb{O}}(\mathcal{E}) = \max_{\psi} R_{\mathbb{O}}^{\psi}(\mathcal{E}), \tag{27}$$

where

$$R_{\mathbb{O}}^{\psi}(\mathcal{E}) = \max\left\{ \langle X, \mathrm{id} \otimes \mathcal{E}(\psi)\rangle \;\middle|\; X \geq 0,\ \langle X, \mathrm{id} \otimes \mathcal{M}(\psi)\rangle \leq 1\ \forall \mathcal{M} \in \mathbb{O}\right\}. \tag{28}$$

Now, for the given target channel $\mathcal{U} : C \to C$, let $\psi^\star \in \mathbb{D}(RC)$ with $R \cong C$ denote a state such that

$$F_{\mathbb{O}}(\mathcal{U}) = \max_{\mathcal{M}\in\mathbb{O}} \left\langle \mathrm{id} \otimes \mathcal{U}(\psi^\star), \mathrm{id} \otimes \mathcal{M}(\psi^\star) \right\rangle. \tag{29}$$

Notice now that $\frac{1}{F_{\mathbb{O}}(\mathcal{U})} \mathrm{id} \otimes \mathcal{U}(\psi^\star)$ is a feasible witness to the dual formulation of the robustness $R_{\mathbb{O}}^{\psi^\star}$; specifically, we have that $\mathrm{id} \otimes \mathcal{U}(\psi^\star) \geq 0$ and

$$\max_{\mathcal{M}\in\mathbb{O}} \left\langle \frac{\mathrm{id} \otimes \mathcal{U}(\psi^\star)}{F_{\mathbb{O}}(\mathcal{U})}, \mathrm{id} \otimes \mathcal{M}(\psi^\star) \right\rangle = 1 \tag{30}$$

by definition of $\psi^\star$. Using the monotonicity of the robustness under free superchannels, we then get

$$\begin{aligned} R_{\mathbb{O}}(\mathcal{E}) &\geq R_{\mathbb{O}}(\Theta(\mathcal{E})) \\ &= R_{\mathbb{O}}(\mathcal{N}) \\ &= \max_{\psi} R_{\mathbb{O}}^{\psi}(\mathcal{N}) \\ &\geq R_{\mathbb{O}}^{\psi^\star}(\mathcal{N}) \\ &\geq \left\langle \mathrm{id} \otimes \mathcal{N}(\psi^\star), \frac{\mathrm{id} \otimes \mathcal{U}(\psi^\star)}{F_{\mathbb{O}}(\mathcal{U})} \right\rangle \\ &\geq \frac{1 - \varepsilon}{F_{\mathbb{O}}(\mathcal{U})}, \end{aligned} \tag{31}$$

where in the last inequality we used the fact that

$$\begin{aligned} 1-\varepsilon &\le F(\mathcal{N},\mathcal{U}) \\ &\le F(\mathrm{id}\otimes\mathcal{N}(\psi^\star),\mathrm{id}\otimes\mathcal{U}(\psi^\star)) \\ &= \left\langle \mathrm{id}\otimes\mathcal{N}(\psi^\star),\mathrm{id}\otimes\mathcal{U}(\psi^\star)\right\rangle \end{aligned} \tag{32}$$

where the first line is by assumption, second by definition of $F(\mathcal{N},\mathcal{U})$, and third since $\mathrm{id}\otimes\mathcal{U}(\psi^\star)$ is rank one. ■

Proposition 9. *If there exists a free supermap $\Theta\in\mathbb{S}$ such that $\Theta(\mathcal{E})=\mathcal{N}$ for some channel $\mathcal{N}$ with $F(\mathcal{N},\mathcal{U})\ge 1-\varepsilon$, then*

$$\varepsilon \ge (1-F_{\mathbb{O}}(\mathcal{U}))\,W_{\mathbb{O}}(\mathcal{E}). \tag{33}$$

Proof. Using Lem. 5 we have once again that

$$W_{\mathbb{O}}(\mathcal{E}) = \min_{\psi} W_{\mathbb{O}}^{\psi}(\mathcal{E}), \tag{34}$$

where

$$W_{\mathbb{O}}^{\psi}(\mathcal{E}) = \min\left\{ \langle X,\mathrm{id}\otimes\mathcal{E}(\psi)\rangle \;\middle|\; X\ge 0,\ \langle X,\mathrm{id}\otimes\mathcal{M}(\psi)\rangle \ge 1\ \forall\mathcal{M}\in\mathbb{O}\right\}. \tag{35}$$

In a way similar to the proof of Prop. 8, we let $\psi^\star\in\mathbb{D}(RC)$ be a state achieving the minimum for $F_{\mathbb{O}}(\mathcal{U})$. We then notice that $\mathbb{1}_{RC}-\mathrm{id}\otimes\mathcal{U}(\psi^\star)\ge 0$ and that for each $\mathcal{M}\in\mathbb{O}$ we have

$$\begin{aligned} &\left\langle \mathrm{id}\otimes\mathcal{M}(\psi^\star),\mathbb{1}-\mathrm{id}\otimes\mathcal{U}(\psi^\star)\right\rangle \\ &= 1-\left\langle \mathrm{id}\otimes\mathcal{M}(\psi^\star),\mathrm{id}\otimes\mathcal{U}(\psi^\star)\right\rangle \\ &\ge 1-\max_{\mathcal{M}\in\mathbb{O}}\left\langle \mathrm{id}\otimes\mathcal{M}(\psi^\star),\mathrm{id}\otimes\mathcal{U}(\psi^\star)\right\rangle \\ &= 1-F_{\mathbb{O}}(\mathcal{U}), \end{aligned} \tag{36}$$

which means that $\frac{1}{1-F_{\mathbb{O}}(\mathcal{U})}(\mathbb{1}-\mathrm{id}\otimes\mathcal{U}(\psi^\star))$ is a valid feasible dual solution for $W_{\mathbb{O}}^{\psi}$. Using the reverse monotonicity of $W_{\mathbb{O}}(\Theta)$, we then get

$$\begin{aligned} W_{\mathbb{O}}(\mathcal{E}) &\le W_{\mathbb{O}}(\mathcal{N}) \\ &\le W_{\mathbb{O}}^{\psi^\star}(\mathcal{N}) \\ &\le \left\langle \mathrm{id}\otimes\mathcal{N}(\psi^\star), \frac{\mathbb{1}-\mathrm{id}\otimes\mathcal{U}(\psi^\star)}{1-F_{\mathbb{O}}(\mathcal{U})}\right\rangle, \end{aligned} \tag{37}$$

and using Eq. (32) we conclude that

$$W_{\mathbb{O}}(\mathcal{E}) \le \frac{1-(1-\varepsilon)}{1-F_{\mathbb{O}}(\mathcal{U})} = \frac{\varepsilon}{1-F_{\mathbb{O}}(\mathcal{U})} \tag{38}$$

which is precisely the statement of the Proposition. ■

2.2. State-based resources

Although the idea of distilling noisy resources into pure ones makes sense in many physical settings, some resource theories are instead concerned with extracting state-based resources. Here, we will assume that there is an underlying set of free states $\mathbb{F}$, and the operations $\mathbb{O}$ are free operations in this theory. In such cases, the target channel in distillation can be chosen as the replacement channel $\mathcal{R}_\phi$, which substitutes any input with a target state: $\mathcal{R}_\phi(\cdot)=\mathrm{Tr}(\cdot)\phi$. A special case of such channels are preparation channels $\mathcal{P}_\phi$, which have trivial input and simply prepare a single copy of a chosen resourceful pure state ϕ. To characterise the resourcefulness of the target channel, we consider the overlap

$$F_{\mathbb{F}}(\phi) = \max_{\sigma\in\mathbb{F}} \langle \phi,\sigma\rangle\,. \tag{39}$$

We then obtain an analogous bound for all transformations into replacement channels.

Theorem 1(b). *If there exists a free superchannel $\Theta \in \mathbb{S}$ such that $F(\Theta(\mathcal{E}), \mathcal{R}_\phi) \geq 1 - \varepsilon$ for some resourceful pure state ϕ, then*

$$\varepsilon \geq 1 - F_{\mathbb{F}}(\phi)\, R_{\mathbb{O}}(\mathcal{E}), \tag{40}$$
$$\varepsilon \geq [1 - F_{\mathbb{F}}(\phi)]\, W_{\mathbb{O}}(\mathcal{E}). \tag{41}$$

Once again, the weight bound (41) gives a no-go result: no resourceful pure state replacement channel can be distilled with $\varepsilon = 0$ from a channel $\mathcal{E}$ such that $W_{\mathbb{O}}(\mathcal{E}) > 0$, that is, such that $\mathrm{supp}(J_{\mathcal{E}})$ contains any free channels.

As a special case, the results apply also to the manipulation of states themselves, that is, transformations of resourceful quantum states under free transformations in the form of channels $\mathbb{O}$. Thus, we obtain:

Corollary 10. *If there exists a free channel $\mathcal{M} \in \mathbb{O}$ such that $F(\mathcal{M}(\rho), \phi) \geq 1 - \varepsilon$ for some resourceful pure state ϕ, then*

$$\varepsilon \geq 1 - F_{\mathbb{F}}(\phi)\, R_{\mathbb{F}}(\rho), \tag{42}$$
$$\varepsilon \geq [1 - F_{\mathbb{F}}(\phi)]\, W_{\mathbb{F}}(\rho). \tag{43}$$

Here we note that an analogous robustness bound for states (42) previously appeared in Ref. [59].

Another approach to no-go results in the distillation of resources from quantum states was studied in Ref. [75]. Our new weight-based bound (43) strictly improves on that result. Let us explicitly compare our result with the bound of Ref. [75], which applies only to full-rank input states ρ, and is given by $\varepsilon \geq [1 - F_{\mathbb{F}}(\phi)]\, \lambda_{\min}(\rho)$, where $\lambda_{\min}$ denotes the smallest eigenvalue of ρ. First, our bounds require no assumption about the rank of the input state ρ, thus extending the applicability of the fundamental restrictions on quantum resource distillation. More importantly, our approach replaces the dependence on the eigenvalues of the input state with a bound which explicitly takes into consideration the resources contained in ρ, which provides more accurate restrictions. Further, for a full-rank state one can notice that $\lambda_{\min}$ can be written as $\min_{\omega \in \mathbb{D}} \max \left\{ \lambda \,\middle|\, \rho \geq \lambda \omega \right\}$. From this it follows that $W_{\mathbb{F}}(\rho) \geq \lambda_{\min}(\rho)$ in any resource theory (with the inequality typically strict), and so the weight-based bound in Eq. (43) is tighter than the result of Ref. [75]. Indeed, in Supplementary Note 4 we will see this improvement to be significant.

As before, we split the proof for clarity.

Proposition 11. *Consider the replacement channel $\mathcal{R}_\phi : C \to D$. If there exists a free supermap Θ such that $\Theta(\mathcal{E}) = \mathcal{N}$ for some channel $\mathcal{N} : C \to D$ with $F(\mathcal{N}, \mathcal{R}_\phi) \geq 1 - \varepsilon$, then*

$$\varepsilon \geq (1 - F_{\mathbb{F}}(\phi))\, W_{\mathbb{O}}(\mathcal{E}). \tag{44}$$

When the input is a preparation channel $\mathcal{P}_\rho : \mathbb{C} \to B$ and the target is the preparation channel $\mathcal{P}_\phi : \mathbb{C} \to D$, the problem reduces to manipulating quantum states, and we have

$$\varepsilon \geq (1 - F_{\mathbb{F}}(\phi))\, W_{\mathbb{F}}(\rho). \tag{45}$$

Proof. Noticing that, for a fixed $\mathcal{E}$, the function $W^{\psi}_{\mathbb{O}}(\mathcal{E})$ that we considered in Prop. 9 is concave in ψ, we can relax the optimisation to write

$$W_{\mathbb{O}}(\mathcal{E}) = \min_{\rho_{RA}} W^{\rho}_{\mathbb{O}}(\mathcal{E}) \tag{46}$$

since the minimum will be achieved on a pure state ψ_{RA} anyway. Choosing $\rho^\star = \rho \otimes \tau$ for arbitrary $\rho \in \mathbb{D}(R)$ and $\tau \in \mathbb{F}$, we

use the reverse monotonicity of $W_{\mathbb{O}}$ to obtain

$$\begin{aligned} W_{\mathbb{O}}(\mathcal{E}) &\leq W_{\mathbb{O}}(\mathcal{N}) \\ &\leq W_{\mathbb{O}}^{\rho^\star}(\mathcal{N}) \\ &= \max\left\{\lambda \;\middle|\; \mathrm{id}\otimes\mathcal{N}(\rho^\star) \geq \mathrm{id}\otimes\mathcal{M}(\rho^\star),\ \mathcal{M}\in\mathbb{O}\right\} \\ &= \max\left\{\lambda \;\middle|\; \mathcal{N}(\tau) \geq \mathcal{M}(\tau),\ \mathcal{M}\in\mathbb{O}\right\} \\ &\leq \max\left\{\lambda \;\middle|\; \mathcal{N}(\tau) \geq \sigma,\ \sigma\in\mathbb{F}\right\} \\ &= W_{\mathbb{F}}(\mathcal{N}(\tau)) \end{aligned} \tag{47}$$

where we used that $\mathcal{M}(\tau)\in\mathbb{F}$. Notice now that $\mathbb{1}-\phi\geq 0$ and $\langle \mathbb{1}-\phi,\sigma\rangle \geq 1-F_{\mathbb{F}}(\phi)\ \forall\sigma\in\mathbb{F}$, which means that $\frac{\mathbb{1}-\phi}{1-F_{\mathbb{F}}(\phi)}$ is a valid feasible dual solution for $W_{\mathbb{F}}(\mathcal{N}(\tau))$. Using the fact that

$$\begin{aligned} 1-\varepsilon &\leq F(\mathcal{N},\mathcal{R}_\phi) \\ &\leq F(\mathrm{id}\otimes\mathcal{N}(\rho^\star),\mathrm{id}\otimes\mathcal{R}_\phi(\rho^\star)) \\ &= F(\mathcal{N}(\tau),\phi) \\ &= \langle \mathcal{N}(\tau),\phi\rangle, \end{aligned} \tag{48}$$

we then have

$$\begin{aligned} W_{\mathbb{F}}(\mathcal{N}(\tau)) &\leq \left\langle \mathcal{N}(\tau), \frac{\mathbb{1}-\phi}{1-F_{\mathbb{F}}(\phi)}\right\rangle \\ &\leq \frac{\varepsilon}{1-F_{\mathbb{F}}(\phi)} \end{aligned} \tag{49}$$

which concludes the proof.

The above reduces to the case of quantum states when the input and target are preparation channels, since this constrains any output of the transformation to also be a preparation channel. ■

Proposition 12. *If there exists a free supermap Θ such that $\Theta(\mathcal{E})=\mathcal{N}$ for some channel $\mathcal{N}: C\to D$ with $F(\mathcal{N},\mathcal{R}_\phi)\geq 1-\varepsilon$, then*

$$\varepsilon \geq 1 - F_{\mathbb{F}}(\phi)\, R_{\mathbb{O}}(\mathcal{E}). \tag{50}$$

If $\mathcal{E}=\mathcal{P}_\rho$ and the system C is trivial, we have that

$$\varepsilon \geq 1 - F_{\mathbb{F}}(\phi)\, R_{\mathbb{F}}(\rho). \tag{51}$$

Proof. Analogously as in Prop. 11, we choose $\rho^\star=\rho\otimes\tau$ for some $\tau\in\mathbb{F}$ to get

$$\begin{aligned} R_{\mathbb{O}}(\mathcal{E}) &\geq R_{\mathbb{O}}(\mathcal{N}) \\ &\geq R_{\mathbb{O}}^{\rho^\star}(\mathcal{N}) \\ &\geq R_{\mathbb{F}}(\mathcal{N}(\tau)) \\ &\geq \left\langle \mathcal{N}(\tau), \frac{\phi}{F_{\mathbb{F}}(\phi)}\right\rangle \\ &\geq \frac{1-\varepsilon}{F_{\mathbb{F}}(\phi)}, \end{aligned} \tag{52}$$

where in the first line we used the monotonicity of $R_{\mathbb{O}}$, in the second line we used that $R_{\mathbb{O}}^{\psi}$ is convex in ψ so we can optimise over mixed states, and in the fourth line we used that $\phi\geq 0$ and $\langle\phi,\sigma\rangle\leq F_{\mathbb{F}}(\phi)\ \forall\sigma\in\mathbb{F}$ which means that $\frac{\phi}{F_{\mathbb{F}}(\phi)}$ is a valid feasible dual solution for the robustness $R_{\mathbb{F}}(\omega)$. ■

Supplementary Note 3: Many-copy transformations and protocols beyond distillation

3.1. Bounds for many-copy manipulation

Recall that the most general physically realisable manipulation protocols involving multiple quantum channels are dubbed *quantum processes* [76–81]. We will be interested in processes which transform multiple uses of a quantum channel into one output map — we thus understand a quantum process Υ as any transformation such that $\Upsilon(\mathcal{N}_1, \ldots, \mathcal{N}_n) \in \mathrm{CPTP}$ for any $\mathcal{N}_1, \ldots, \mathcal{N}_n \in \mathrm{CPTP}$, and we take the set of free quantum processes as

$$\mathbb{S}_{(n)} := \left\{ \Upsilon \;\middle|\; \Upsilon(\mathcal{M}_1, \ldots, \mathcal{M}_n) \in \mathbb{O} \;\; \forall \mathcal{M}_1, \ldots, \mathcal{M}_n \in \mathbb{O} \right\}. \tag{53}$$

Depending on the given resource theory, different ways to manipulate multiple channels might be of interest. For instance, when the theory is closed under tensor product, i.e. $\mathcal{M}, \mathcal{M}' \in \mathbb{O} \Rightarrow \mathcal{M} \otimes \mathcal{M}' \in \mathbb{O}$, then any free protocol which manipulates n copies of a channel in parallel as $\Theta(\mathcal{E}^{\otimes n})$ is a free quantum process. Similarly, when the theory is closed under composition, i.e. $\mathcal{M}, \mathcal{M}' \in \mathbb{O} \Rightarrow \mathcal{M} \circ \mathcal{M}' \in \mathbb{O}$, then any sequential protocol of the form $\Theta_n(\mathcal{E}) \circ \cdots \circ \Theta_1(\mathcal{E})$ belongs to the set $\mathbb{S}_{(n)}$. However, a general channel theory need not be closed under tensor product or composition — for instance, the tensor product of operations which preserve separability in entanglement theory is not always separability preserving itself [82]. Therefore, to take into consideration the most general way of manipulating quantum channels allowed by the constraints of the given resource theory, we employ the formalism of free quantum processes $\mathbb{S}_{(n)}$. By considering such transformations, we can establish fundamental bounds on the performance of any adaptive, multi-copy protocol for manipulating channels or states.

Theorem 2. *Given any distillation protocol $\Upsilon \in \mathbb{S}_{(n)}$ — parallel, sequential, or adaptive, with or without a definite causal order — which transforms n uses of a channel $\mathcal{E}$ to some target unitary $\mathcal{U}$ up to accuracy $\varepsilon > 0$, it necessarily holds that*

$$n \geq \log_{1/W_{\mathbb{O}}(\mathcal{E})} \frac{1 - F_{\mathbb{O}}(\mathcal{U})}{\varepsilon}, \tag{54}$$

$$n \geq \log_{R_{\mathbb{O}}(\mathcal{E})} \frac{1 - \varepsilon}{F_{\mathbb{O}}(\mathcal{U})}. \tag{55}$$

Analogously, when the target channel is a replacement channel $\mathcal{R}_\phi$ which prepares a pure state ϕ, we have

$$n \geq \log_{1/W_{\mathbb{O}}(\mathcal{E})} \frac{1 - F_{\mathbb{F}}(\phi)}{\varepsilon}, \tag{56}$$

$$n \geq \log_{R_{\mathbb{O}}(\mathcal{E})} \frac{1 - \varepsilon}{F_{\mathbb{F}}(\phi)}. \tag{57}$$

The result applies also to the case of state manipulation, where we obtain that the number of copies of a state needed to perform the distillation $\mathcal{M}(\rho^{\otimes n}) \to \phi$ up to error ε must obey

$$n \geq \log_{1/W_{\mathbb{F}}(\rho)} \frac{1 - F_{\mathbb{F}}(\phi)}{\varepsilon}, \quad n \geq \log_{R_{\mathbb{F}}(\rho)} \frac{1 - \varepsilon}{F_{\mathbb{F}}(\phi)}. \tag{58}$$

This, again, improves on the bound obtained in Ref. [75] for resource theories of states.

The two bounds exhibit very different properties. Intuitively, we see that the weight-based bound (54) will perform better for small ε, establishing in particular that n must scale as $\log(1/\varepsilon)$ as $\varepsilon \to 0$ for distillation to be possible. On the other hand, the robustness-based bound (55) increases in performance with decreasing $F_{\mathbb{O}}(\mathcal{U})$, i.e. with increasing resourcefulness of $\mathcal{U}$. One can use both of these insights to one's advantage when aiming to obtain more accurate bounds. For instance, a straightforward way to decrease $F_{\mathbb{O}}(\mathcal{U})$ is, instead of considering $\mathcal{U}$ as a target channel, to consider several copies of it. In practice, such an approach can be employed in block distillation protocols, which could provide a more efficient way of purifying the given resource. As long as one can bound or compute the quantity $F_{\mathbb{O}}(\mathcal{U}^{\otimes m})$ — which we will shortly see to be possible in relevant cases — this leads to an immediate improvement in the robustness bound (cf. [60]).

The result of Thm. 2 is a consequence of a general sub- or supermultiplicativity result for the monotones $R_{\mathbb{O}}, W_{\mathbb{O}}$.

Theorem 13. *Consider a collection of n channels* $(\mathcal{E}_1, \ldots, \mathcal{E}_n)$. *For any free protocol* $\Upsilon \in \mathbb{S}_{(n)}$ *it holds that*

$$W_{\mathbb{O}}\left(\Upsilon(\mathcal{E}_1, \ldots, \mathcal{E}_n)\right) \geq \prod_i W_{\mathbb{O}}(\mathcal{E}_i). \tag{59}$$

and

$$R_{\mathbb{O}}\left(\Upsilon(\mathcal{E}_1, \ldots, \mathcal{E}_n)\right) \leq \prod_i R_{\mathbb{O}}(\mathcal{E}_i). \tag{60}$$

In particular, for any free protocol $\Upsilon \in \mathbb{S}_{(n)}$ *which takes n uses of a quantum channel* $\mathcal{E}$ *to another quantum channel* $\mathcal{E}'$, *i.e.* $\Upsilon(\mathcal{E}, \ldots, \mathcal{E}) = \mathcal{E}'$, *it necessarily holds that*

$$n \geq \frac{\log R_{\mathbb{O}}(\mathcal{E}')}{\log R_{\mathbb{O}}(\mathcal{E})}, \quad n \geq \frac{\log W_{\mathbb{O}}(\mathcal{E}')}{\log W_{\mathbb{O}}(\mathcal{E})}, \tag{61}$$

where in the second inequality we take $\log 0 = -\infty$ *and assume that* $W_{\mathbb{O}}(\mathcal{E}')$ *and* $W_{\mathbb{O}}(\mathcal{E})$ *are not both 0.*

Notice that this establishes bounds which go beyond distillation protocols, and imposes constraints on arbitrary manipulation of channels. In particular, the robustness-based bound gives a general restriction on the capabilities of channel dilution, i.e. transformations $\mathcal{U} \to \mathcal{E}'$ and $\mathcal{R}_\phi \to \mathcal{E}'$, which can be understood as simulating the action of a channel $\mathcal{E}'$ by employing the pure channel $\mathcal{U}$ or $\mathcal{R}_\phi$.

An interesting difference between the bounds of Thm. 13 emerges in the case when $\mathcal{E}'$ is a unitary or pure replacement channel with $W_{\mathbb{O}}(\mathcal{E}') = 0$. Here, the weight-based bound shows that increasing the number of uses of a channel cannot allow perfect distillation when $W_{\mathbb{O}}(\mathcal{E}) \in (0, 1]$, strengthening the no-go result of Thm. 1(a). However, the bound based on $W_{\mathbb{O}}$ does not provide information on the distillation of channels with $W_{\mathbb{O}}(\mathcal{E}) = 0$ — notably, unitary-to-unitary transformations — while the robustness-based bound can also be applied in such cases. This complements the no-go results provided by $W_{\mathbb{O}}$ and can reveal errors even in transformations where the weight bound becomes trivial.

Proof of Thm. 13. We consider $W_{\mathbb{O}}$ first. For each $\mathcal{E}_i$, let $\mu_i \in \mathbb{R}_+$ and $\mathcal{M}_i \in \mathbb{O}$ be such that $J_{\mathcal{E}_i} \geq \mu_i J_{\mathcal{M}_i}$. Using the n-linearity of the transformation Υ, we can expand

$$\begin{aligned}
\Upsilon(\mathcal{E}_1, \ldots, \mathcal{E}_n) &= \Upsilon(\mathcal{E}_1 - \mu_1\mathcal{M}_1, \mathcal{E}_2, \ldots, \mathcal{E}_n) + \Upsilon(\mu_1\mathcal{M}_1, \mathcal{E}_2, \ldots, \mathcal{E}_n)\\
&= \Upsilon(\mathcal{E}_1 - \mu_1\mathcal{M}_1, \mathcal{E}_2, \ldots, \mathcal{E}_n) + \Upsilon(\mu_1\mathcal{M}_1, \mathcal{E}_2 - \mu_2\mathcal{M}_2, \mathcal{E}_3, \ldots, \mathcal{E}_n) + \Upsilon(\mu_1\mathcal{M}_1, \mu_2\mathcal{M}_2, \mathcal{E}_3, \ldots, \mathcal{E}_n)\\
&\vdots\\
&= \Upsilon(\mathcal{E}_1 - \mu_1\mathcal{M}_1, \mathcal{E}_2, \ldots, \mathcal{E}_n) + \ldots + \Upsilon(\mu_1\mathcal{M}_1, \ldots, \mu_{n-1}\mathcal{M}_{n-1}, \mathcal{E}_n - \mu_n\mathcal{M}_n) + \Upsilon(\mu_1\mathcal{M}_1, \ldots, \mu_n\mathcal{M}_n).
\end{aligned} \tag{62}$$

By the positivity of Υ, each term on the right-hand side is positive semidefinite, and so

$$\begin{aligned}
0 &\leq \Upsilon(\mathcal{E}_1, \ldots, \mathcal{E}_n) - \Upsilon(\mu_1\mathcal{M}_1, \ldots, \mu_n\mathcal{M}_n)\\
&= \Upsilon(\mathcal{E}_1, \ldots, \mathcal{E}_n) - \left(\prod_i \mu_i\right)\Upsilon(\mathcal{M}_1, \ldots, \mathcal{M}_n)\\
&= \Upsilon(\mathcal{E}_1, \ldots, \mathcal{E}_n) - \left(\prod_i \mu_i\right)\mathcal{M}'
\end{aligned} \tag{63}$$

for some $\mathcal{M}' \in \mathbb{O}$ due to the fact that Υ is a free quantum process. Choosing $\mathcal{M}_i$ as optimal channels such that $\mu_i = W_{\mathbb{O}}(\mathcal{E}_i)$, we have that $\prod_i W_{\mathbb{O}}(\mathcal{E}_i)$ is a feasible optimal value for $W_{\mathbb{O}}\left(\Upsilon(\mathcal{E}_1, \ldots, \mathcal{E}_n)\right)$, which is precisely Eq. (59).

The case of the robustness $R_{\mathbb{O}}$ is shown analogously: recalling that $R_{\mathbb{O}}(\mathcal{E})$ is given by the least coefficient such that $J_{\mathcal{E}_i} \leq \mu_i J_{\mathcal{M}_i}$ for $\mathcal{M}_i \in \mathbb{O}$, we use the positivity and n-linearity of Υ to show Eq. (60) by the same argument. ∎

Proof of Thm. 2. Let $\Upsilon \in \mathbb{S}_{(n)}$ be any general distillation protocol such that $F\left(\Upsilon(\mathcal{E}^{\times n}), \mathcal{U}\right) \geq 1 - \varepsilon$, where we use $\mathcal{E}^{\times n}$ to denote the n-tuple $(\mathcal{E}, \mathcal{E}, \ldots, \mathcal{E})$ representing n uses of $\mathcal{E}$. Using Thm. 13 and Prop. 9 gives

$$\begin{aligned}
W_{\mathbb{O}}(\mathcal{E})^n &\leq W_{\mathbb{O}}(\Upsilon(\mathcal{E}^{\times n}))\\
&\leq \frac{\varepsilon}{1 - F_{\mathbb{O}}(\mathcal{U})}.
\end{aligned} \tag{64}$$

Taking logarithm of both sides of the equation and recalling that $W_{\mathbb{O}}(\mathcal{E}) \in [0,1]$, we get

$$n \geq \frac{\log \frac{\varepsilon}{1-F_{\mathbb{O}}(\mathcal{U})}}{\log W(\mathcal{E})} \tag{65}$$

as claimed.

The case of the robustness $R_{\mathbb{O}}$ follows analogously by using Thm. 13 and Prop. 8.

The state-based case follows in the same way, using Thm. 13 and Prop. 11 or Prop. 12. ■

A consequence of Thm. 13 is the sub- or supermultiplicativity of the measures under tensor product and composition.

Corollary 14. *If the resource theory is closed under tensor product, i.e.* $\mathcal{M}, \mathcal{M}' \in \mathbb{O} \Rightarrow \mathcal{M} \otimes \mathcal{M}' \in \mathbb{O}$, *then*

$$\begin{aligned} R_{\mathbb{O}}\left(\mathcal{E}_1 \otimes \mathcal{E}_2\right) &\leq R_{\mathbb{O}}(\mathcal{E}_1) R_{\mathbb{O}}(\mathcal{E}_2), \\ W_{\mathbb{O}}\left(\mathcal{E}_1 \otimes \mathcal{E}_2\right) &\geq W_{\mathbb{O}}(\mathcal{E}_1) W_{\mathbb{O}}(\mathcal{E}_2). \end{aligned} \tag{66}$$

If the resource theory is closed under composition, i.e. $\mathcal{M}, \mathcal{M}' \in \mathbb{O} \Rightarrow \mathcal{M} \circ \mathcal{M}' \in \mathbb{O}$, *then*

$$\begin{aligned} R_{\mathbb{O}}\left(\mathcal{E}_1 \circ \mathcal{E}_2\right) &\leq R_{\mathbb{O}}(\mathcal{E}_1) R_{\mathbb{O}}(\mathcal{E}_2), \\ W_{\mathbb{O}}\left(\mathcal{E}_1 \circ \mathcal{E}_2\right) &\geq W_{\mathbb{O}}(\mathcal{E}_1) W_{\mathbb{O}}(\mathcal{E}_2). \end{aligned} \tag{67}$$

3.2. Asymptotic rates of distillation

To understand the ultimate limitations on transforming a given state or channel, one can study the maximal rate at which the conversion can be performed with an asymptotic number of channel uses, allowing for conversion error that vanishes asymptotically. Specifically, given two channels $\mathcal{E}, \mathcal{F}$, we define the maximal achievable rate of transformation under any adaptive protocol as

$$r_{\text{adap}}(\mathcal{E} \to \mathcal{F}) := \sup \left\{ r \,\middle|\, \lim_{n\to\infty} \sup_{\Upsilon_n \in \mathbb{S}_{(n)}} F\left(\Upsilon_n(\mathcal{E}^{\times n}), \mathcal{F}^{\otimes \lfloor rn \rfloor}\right) = 1 \right\}, \tag{68}$$

Again, the transformations that we consider include both parallel and sequential protocols as relevant special cases, and thus provide an upper bound for both. Although (68) characterises the conversion rate with the perfect fidelity in asymptotic limit, it does not give insights into how robust the rate is against error. To characterise the maximum rate at which the asymptotic transformation is possible with some non-vanishing error, we define the strong converse rate as [83]

$$r^{\dagger}_{\text{adap}}(\mathcal{E} \to \mathcal{F}) := \sup \left\{ r \,\middle|\, \limsup_{n\to\infty} \sup_{\Upsilon_n \in \mathbb{S}_{(n)}} F\left(\Upsilon_n(\mathcal{E}^{\times n}), \mathcal{F}^{\otimes \lfloor rn \rfloor}\right) > 0 \right\}. \tag{69}$$

In other words, as soon as a rate exceeds the strong converse rate, the fidelity necessarily goes to 0. This places a threshold for the achievable distillation rates, even when non-zero error is allowed.

Applying our result in Thm. 2 allows us to obtain a general bound on the rate of distillation protocols.

Theorem 3(a). *If the target channel* $\mathcal{U}$ *satisfies* $F_{\mathbb{O}}(\mathcal{U}^{\otimes n}) = F_{\mathbb{O}}(\mathcal{U})^n$, *then we have a strong converse bound as*

$$r_{\text{adap}}(\mathcal{E} \to \mathcal{U}) \leq r^{\dagger}_{\text{adap}}(\mathcal{E} \to \mathcal{U}) \leq \frac{\log R_{\mathbb{O}}(\mathcal{E})}{\log F_{\mathbb{O}}(\mathcal{U})^{-1}}. \tag{70}$$

Alternatively, if the target is a replacement channel $\mathcal{R}_\phi$ *such that* $F_{\mathbb{F}}(\phi^{\otimes n}) = F_{\mathbb{F}}(\phi)^n$, *then*

$$r_{\text{adap}}(\mathcal{E} \to \mathcal{R}_\phi) \leq r^{\dagger}_{\text{adap}}(\mathcal{E} \to \mathcal{R}_\phi) \leq \frac{\log R_{\mathbb{O}}(\mathcal{E})}{\log F_{\mathbb{F}}(\phi)^{-1}}. \tag{71}$$

In the above, $F_{\mathbb{O}}(\mathcal{U})$ *(or* $F_{\mathbb{F}}(\phi)$*) can be replaced with any other multiplicative quantity* $G_{\mathbb{O}}(\mathcal{U})$ *s.t.* $F_{\mathbb{O}}(\mathcal{U}) \leq G_{\mathbb{O}}(\mathcal{U})$ *(or* $F_{\mathbb{F}}(\phi) \leq G_{\mathbb{F}}(\phi)$*), and the results hold analogously.*

Proof. Let r be any achievable rate at error threshold $\varepsilon \in [0,1)$, that is, assume that there exists a sequence $\{\Upsilon_n\}_n$ of free quantum processes such that $1 - F\left(\Upsilon_n(\mathcal{E}^{\times n}), \mathcal{U}^{\otimes \lfloor rn \rfloor}\right) =: \varepsilon_n$ with $\liminf_{n\to\infty} \varepsilon_n = \varepsilon < 1$. From Thm. 2, for each n we have that

$$\begin{aligned} n \log R_{\mathbb{O}}(\mathcal{E}) &\geq \log(1-\varepsilon_n) - \log F_{\mathbb{O}}\left(\mathcal{U}^{\otimes \lfloor rn \rfloor}\right) \\ &\geq \log(1-\varepsilon_n) + rn \log F_{\mathbb{O}}(\mathcal{U})^{-1} \end{aligned} \tag{72}$$

where we used the multiplicativity of $F_{\mathbb{O}}(\mathcal{U})$. Dividing by n and taking $\limsup_{n\to\infty}$ in both sides gives the claimed result.

When the target is a replacement channel, we can follow the proof of Lem. 7 to get

$$F_{\mathbb{O}}(\mathcal{R}_\phi^{\otimes n}) \leq F_{\mathbb{F}}(\phi^{\otimes n}) \tag{73}$$

and the rest of the proof proceeds analogously. ■

In some cases, it is reasonable to restrict our attention to parallel protocols (illustrated in Fig. 2(a) of the main text), which are indeed how many communication and channel manipulation schemes are often considered [71, 72, 84]. To separately characterise this scenario, we define the rate of transformation with parallel protocols as

$$r_{\text{par}}(\mathcal{E} \to \mathcal{F}) := \sup\left\{ r \,\middle|\, \lim_{n\to\infty} \sup_{\Theta_n \in \mathbb{S}} F\left(\Theta_n(\mathcal{E}^{\otimes n}), \mathcal{F}^{\otimes \lfloor rn \rfloor}\right) = 1 \right\}, \tag{74}$$

and its strong converse rate $r^\dagger_{\text{par}}(\mathcal{E} \to \mathcal{F})$ analogously. Then, we can get better strong converse bounds for parallel protocols by suitably 'smoothing' the definition of the robustness over all channels within a small distance of the original input $\mathcal{E}$ [4, 5, 7, 21]. We thus define the regularised log-robustness (max-relative entropy) $D^\infty_{\mathbb{O}}(\mathcal{E}) := \lim_{\delta\to 0} \limsup_{n\to\infty} \frac{1}{n} \log R^\delta_{\mathbb{O}}(\mathcal{E}^{\otimes n})$ where $R^\delta_{\mathbb{O}}(\mathcal{E}) := \min_{F(\tilde{\mathcal{E}},\mathcal{E}) \geq 1-\delta} R_{\mathbb{O}}(\tilde{\mathcal{E}})$, and use it as follows.

Theorem 3(b). *If the target channel $\mathcal{U}$ satisfies $F_{\mathbb{O}}(\mathcal{U}^{\otimes n}) = F_{\mathbb{O}}(\mathcal{U})^n$, then*

$$r_{\text{par}}(\mathcal{E} \to \mathcal{U}) \leq r^\dagger_{\text{par}}(\mathcal{E} \to \mathcal{U}) \leq \frac{D^\infty_{\mathbb{O}}(\mathcal{E})}{\log F_{\mathbb{O}}(\mathcal{U})^{-1}}. \tag{75}$$

Alternatively, if the target is a preparation channel $\mathcal{P}_\phi$ such that $F_{\mathbb{F}}(\phi^{\otimes n}) = F_{\mathbb{F}}(\phi)^n$, then we have a strong converse bound as

$$r_{\text{par}}(\mathcal{E} \to \mathcal{P}_\phi) \leq r^\dagger_{\text{par}}(\mathcal{E} \to \mathcal{P}_\phi) \leq \frac{D^\infty_{\mathbb{O}}(\mathcal{E})}{\log F_{\mathbb{F}}(\phi)^{-1}}. \tag{76}$$

In the above, $F_{\mathbb{O}}(\mathcal{U})$ (or $F_{\mathbb{F}}(\phi)$) can be replaced with any other multiplicative quantity $G_{\mathbb{O}}(\mathcal{U})$ s.t. $F_{\mathbb{O}}(\mathcal{U}) \leq G_{\mathbb{O}}(\mathcal{U})$ (or $F_{\mathbb{F}}(\phi) \leq G_{\mathbb{F}}(\phi)$), and the results hold analogously.

For both of the results of this section to be valid, we require the multiplicativity of the channel fidelity of the target unitary $\mathcal{U}$ or state ϕ. In practice, the bound can be relaxed by using the Choi fidelity $\widetilde{F_{\mathbb{O}}}(\mathcal{U}) \geq F_{\mathbb{O}}(\mathcal{U})$, which is often easier to show to be multiplicative. Although the multiplicativity condition might not hold in full generality, the majority of relevant resource theories do indeed satisfy it. This includes (cf. Table I in the main text):

- The theories of communication and quantum memories with the choice of the target channel $\mathcal{U} = \text{id}$, where we have $F_{\mathbb{O}}(\text{id}^{\otimes n}) = F_{\mathbb{O}}(\text{id})^n$ (see Supplementary Note 4.1).
- The theory of magic for multi-qubit quantum channels. Here, the fidelity $F_{\mathbb{O}}(\mathcal{R}_\phi)$ of any replacement channel $\mathcal{R}_\phi$ reduces to $F_{\mathbb{F}}(\phi)$ which is known to be multiplicative as long as ϕ is a state of up to three qubits [85]. We will furthermore show in Supplementary Note 4.2 the multiplicativity of the channel fidelity $F_{\mathbb{O}}(\mathcal{U})$ of any 1-, 2-, or 3-qubit diagonal unitary channel from the third level of the Clifford hierarchy, which are common choices of target channels in practical settings.
- The theory of magic for multi-qudit quantum channels, where a multiplicative bound for the fidelity of any target replacement channel $\mathcal{R}_\phi$ is given by the min-thauma of magic [27, 86]. The fidelity $F_{\mathbb{F}}$ itself can also be shown to be multiplicative in some relevant cases.
- Other state-based theories such as entanglement, coherence, athermality, and purity, where the quantities $F_{\mathbb{F}}(\phi)$ are known to be multiplicative for any target pure state ϕ.

Our results therefore give broadly applicable bounds for the asymptotic performance of general distillation protocols.

In order to prove Thm. 3(b), we first show the following lemma.

Lemma 15. *For any two channels $\mathcal{E}, \mathcal{N}$ and unitary $\mathcal{U}$, it holds that*

$$|F(\mathcal{E},\mathcal{U}) - F(\mathcal{N},\mathcal{U})| \leq \sqrt{1 - F(\mathcal{E},\mathcal{N})}. \tag{77}$$

Proof. We first show $|F(\mathcal{E},\mathcal{U}) - F(\mathcal{N},\mathcal{U})| \leq \frac{1}{2}\|\mathcal{E}-\mathcal{N}\|_\diamond$. Suppose $F(\mathcal{E},\mathcal{U}) \geq F(\mathcal{N},\mathcal{U})$ without loss of generality. Then,

$$\begin{aligned}
&|F(\mathcal{E},\mathcal{U}) - F(\mathcal{N},\mathcal{U})| \\
&= F(\mathcal{E},\mathcal{U}) - F(\mathcal{N},\mathcal{U}) \\
&= \min_\phi \langle\phi|(\mathrm{id}\otimes\mathcal{U}^\dagger)\mathrm{id}\otimes\mathcal{E}(\phi)(\mathrm{id}\otimes\mathcal{U})|\phi\rangle \\
&\quad - \min_\phi \langle\phi|(\mathrm{id}\otimes\mathcal{U}^\dagger)\mathrm{id}\otimes\mathcal{N}(\phi)(\mathrm{id}\otimes\mathcal{U})|\phi\rangle \\
&\leq \langle\tilde{\phi}_\mathcal{U}|\mathrm{id}\otimes\mathcal{E}(\tilde{\phi}) - \mathrm{id}\otimes\mathcal{N}(\tilde{\phi})|\tilde{\phi}_\mathcal{U}\rangle \\
&\leq \max_\phi \max_{0\leq M\leq \mathbb{1}} \langle M, \mathrm{id}\otimes\mathcal{E}(\phi) - \mathrm{id}\otimes\mathcal{N}(\phi)\rangle \\
&= \frac{1}{2}\|\mathcal{E}-\mathcal{N}\|_\diamond
\end{aligned} \tag{78}$$

where in the third line we defined $\tilde{\phi}$ to be the minimiser of the second term in the second line and also defined $\tilde{\phi}_\mathcal{U} = \mathrm{id}\otimes\mathcal{U}(\tilde{\phi})$, and in the fourth line we used that $0 \leq |\tilde{\phi}_\mathcal{U}\rangle\langle\tilde{\phi}_\mathcal{U}| \leq \mathbb{1}$.

The proof is concluded by noticing $\frac{1}{2}\|\mathcal{E}-\mathcal{N}\|_\diamond \leq \sqrt{1-F(\mathcal{E},\mathcal{N})}$ [43, 46].

■

Proof of Thm. 3(b). Let r be any achievable rate at error threshold $\varepsilon \in [0,1)$, that is, assume that there exists a sequence $\{\Theta_n\}_n$ of free superchannels such that $1 - F\left(\Theta_n(\mathcal{E}^{\otimes n}), \mathcal{U}^{\otimes\lfloor rn\rfloor}\right) =: \varepsilon_n$ with $\liminf_{n\to\infty}\varepsilon_n = \varepsilon < 1$. Let δ with $0 < \delta \leq 1$ be some constant and $\mathcal{N}_n$ be a channel such that $\log R^\delta_\mathbb{O}(\Theta_n(\mathcal{E}^{\otimes n})) = \log R_\mathbb{O}(\mathcal{N}_n)$. Then, using Lem. 15,

$$\begin{aligned}
F(\mathcal{N}_n, \mathcal{U}^{\otimes\lfloor rn\rfloor}) &\geq F(\Theta_n(\mathcal{E}^{\otimes n}), \mathcal{U}^{\otimes\lfloor rn\rfloor}) - \sqrt{1 - F(\mathcal{N}_n, \Theta_n(\mathcal{E}^{\otimes n}))} \\
&\geq 1 - \varepsilon_n - \sqrt{\delta}.
\end{aligned} \tag{79}$$

Since doing nothing is a valid free comb, $\mathcal{N}_n$ can be transformed to $\mathcal{U}^{\otimes\lfloor rn\rfloor}$ with fidelity $1-\varepsilon_n-\sqrt{\delta}$ for free. Applying Thm. 2, we get

$$\begin{aligned}
\varepsilon_n + \sqrt{\delta} &\geq 1 - R_\mathbb{O}(\mathcal{N}_n) F_\mathbb{O}\left(\mathcal{U}^{\otimes\lfloor rn\rfloor}\right) \\
&= 1 - R_\mathbb{O}(\mathcal{N}_n) F_\mathbb{O}(\mathcal{U})^{\lfloor rn\rfloor}
\end{aligned} \tag{80}$$

where we used the assumption of the multiplicativity $F_\mathbb{O}\left(\mathcal{U}^{\otimes m}\right) = F_\mathbb{O}(\mathcal{U})^m$. Let $\{m_n\}_n$ be a subsequence such that $\lim_{n\to\infty}\varepsilon_{m_n} = \varepsilon$. Then, since $0 \leq \varepsilon < 1$, there exist some integer N and positive real number δ' such that $0 < \varepsilon_{m_n} + \sqrt{\delta'} < 1$ for any $n > N$. Using (80), we get for $n > N$ and $\delta \leq \delta'$ that

$$\begin{aligned}
r &\leq \frac{\lfloor rm_n\rfloor + 1}{m_n} \\
&\leq \frac{1}{\log F_\mathbb{O}(\mathcal{U})^{-1}} \frac{1}{m_n} \log R_\mathbb{O}(\mathcal{N}_{m_n}) + \frac{1}{\log F_\mathbb{O}(\mathcal{U})^{-1}} \frac{1}{m_n} \log \frac{1}{1 - (\varepsilon_{m_n} + \sqrt{\delta})} + \frac{1}{m_n} \\
&= \frac{1}{\log F_\mathbb{O}(\mathcal{U})^{-1}} \frac{1}{m_n} \log R^\delta_\mathbb{O}(\Theta(\mathcal{E}^{\otimes m_n})) + \frac{1}{\log F_\mathbb{O}(\mathcal{U})^{-1}} \frac{1}{m_n} \log \frac{1}{1 - (\varepsilon_{m_n} + \sqrt{\delta})} + \frac{1}{m_n} \\
&\leq \frac{1}{\log F_\mathbb{O}(\mathcal{U})^{-1}} \frac{1}{m_n} \log R^\delta_\mathbb{O}(\mathcal{E}^{\otimes m_n}) + \frac{1}{\log F_\mathbb{O}(\mathcal{U})^{-1}} \frac{1}{m_n} \log \frac{1}{1 - (\varepsilon_{m_n} + \sqrt{\delta})} + \frac{1}{m_n}
\end{aligned} \tag{81}$$

where in the last inequality we used the monotonicity of max-relative entropy measure under free superchannels [8]. Noting that

$$\limsup_{n\to\infty} \frac{1}{m_n} \log R^\delta_\mathbb{O}(\mathcal{E}^{\otimes m_n}) \leq \limsup_{n\to\infty} \frac{1}{n} \log R^\delta_\mathbb{O}(\mathcal{E}^{\otimes n}), \tag{82}$$

we take $\lim_{\delta\to 0}\limsup_{n\to\infty}$ in both sides of (81) to get

$$r \leq \frac{D^{\infty}_{\mathbb{O}}(\mathcal{E})}{\log F_{\mathbb{O}}(\mathcal{U})^{-1}},\ \forall\varepsilon\in[0,1) \tag{83}$$

showing that the quantity in the right hand side is a strong converse bound. The state case can be shown analogously. ■

Supplementary Note 4: Applications

4.1. Quantum communication

A central goal of quantum communication is to enable reliable transmission of quantum states to another party through a noisy channel. A way of accomplishing this task is to apply encoding (respectively, decoding) operations to input (output) states so that one can offset the effect of noise, and there has been an enormous amount of work to investigate the ultimate limit on how much information can be reliably sent [15, 83, 87–99]. By understanding this problem as the purification of a noisy quantum channel to the identity channel by means of some allowed free channel transformation, we can directly apply the results of our work.

To encompass the most general communication setting, we consider assisted communication scenarios, where both parties may share some correlations (e.g. entanglement) or are able to perform some joint operations (e.g. communicate classically) in order to enhance their quantum communication capabilities [89, 100]. The given type of assistance can be specified by the set of free superchannels in our framework, and our general results can be applied by considering $\mathbb{O}$ as the set of free channels that are preserved by the free superchannels. We will thus investigate the maximum size of quantum systems that can be reliably sent using free superchannels $\mathbb{S}_{\mathcal{A}}$ where $\mathcal{A}$ denotes the type of assisting operations. Of particular interest in the theory of quantum communication is the asymptotic capacity where many uses of the given channel are considered. We define the $\mathcal{A}$-assisted adaptive quantum capacity $Q_{\mathcal{A},\mathrm{adap}}$ as the rate at which the given channel can be converted to the qubit identity channel id_2 using the given choice of superchannels $\mathbb{S}=\mathbb{S}_{\mathcal{A}}$, that is, $r_{\mathrm{adap}}(\mathcal{E}\to\mathrm{id}_2)$ in the notation of Supplementary Note 3.2. Another important quantity is the strong converse capacity, similarly defined as $Q^{\dagger}_{\mathcal{A},\mathrm{adap}}(\mathcal{E}) \coloneqq r^{\dagger}(\mathcal{E}\to\mathrm{id}_2)$. We note that our methods naturally apply also to the setting of generalised resource theories of communication [9], where transformations with an indefinite causal order are allowed.

We also consider a simpler scenario where multiple uses of the given channel are applied in parallel. We define the $\mathcal{A}$-assisted quantum capacity under a parallel communication protocol as $Q_{\mathcal{A}}(\mathcal{E}) \coloneqq r_{\mathrm{par}}(\mathcal{E}\to\mathrm{id}_2)$ and similarly the strong converse capacity as $Q^{\dagger}_{\mathcal{A}}(\mathcal{E}) \coloneqq r^{\dagger}_{\mathrm{par}}(\mathcal{E}\to\mathrm{id}_2)$.

Considering assisted capacities is insightful in that they serve as upper bounds for capacities with weaker assistance, including the unassisted quantum capacity $Q(\mathcal{E})$ whose single-letter formula is not known and thus hard to analyse in general [101]. Below, we apply our method to two assisted settings that are often considered in the literature.

4.1.1. *No-signalling assistance*

An important class of assisted communication codes are ones where the sending and receiving party are allowed to share no-signalling correlations [8, 93, 102–105]. It is the communication setting where the superchannel $\Theta : (A'\to B)\to(A\to B')$ is realised by a bipartite no-signalling channel $\Pi_{\mathrm{NS}} : AB\to A'B'$ satisfying

$$\begin{aligned}\mathrm{Tr}_{A'}\,\Pi_{\mathrm{NS}}(\rho_A^{(0)}\otimes\rho_B) &= \mathrm{Tr}_{A'}\,\Pi_{\mathrm{NS}}(\rho_A^{(1)}\otimes\rho_B)\\ \mathrm{Tr}_{B'}\,\Pi_{\mathrm{NS}}(\rho_A\otimes\rho_B^{(0)}) &= \mathrm{Tr}_{B'}\,\Pi_{\mathrm{NS}}(\rho_A\otimes\rho_B^{(1)})\end{aligned} \tag{84}$$

for any state ρ_A, ρ_B, and any pair of states $\{\rho_A^{(j)}\}_{j=0}^1$, $\{\rho_B^{(j)}\}_{j=0}^1$. We denote the set of superchannels realised by no-signalling channels as $\mathbb{S}_{\mathrm{NS}}$ and call them no-signalling superchannels. Intuitively, such transformations do not create a side channel that allows for free communication. In this setting, the free channels are the channels which are useless for transmitting any information: this set is formed by the replacement channels $\mathbb{O}_R \coloneqq \left\{\mathcal{R}_\sigma : A'\to B \,\middle|\, \mathcal{R}_\sigma(\cdot)=\mathrm{Tr}(\cdot)\sigma,\ \sigma\in\mathbb{D}(B)\right\}$, which simply replace the input with a fixed output state. Indeed, any superchannel $\mathbb{S}_{\mathrm{NS}}$ preserves the set of replacement channels. Motivated by this, Ref. [8] introduced a resource theory of communication with the set of free superchannels $\mathbb{S} \coloneqq \left\{\Theta \,\middle|\, \Theta(\mathcal{M})\in\mathbb{O}_R\ \forall\mathcal{M}\in\mathbb{O}_R\right\}$, and showed that $\mathbb{S}$ actually coincides with the set of no-signalling superchannels, i.e., $\mathbb{S}=\mathbb{S}_{\mathrm{NS}}$. Many-copy transformations with no-signalling assistance are then formally defined as the transformations by free combs as in (53), which map multiple constant channels to a constant channel. A class of free many-copy transformations are

the quantum feedback-assisted adaptive protocols where the receiving party is allowed to send a part of their quantum system back to the sender, followed by the application of a no-signalling bipartite channel between the channels uses. This includes the quantum feedback-assisted communication with entanglement assistance discussed in Refs. [84, 106].

To evaluate $F_{\mathbb{O}_R}(\mathrm{id}_d)$, we will use the following lemma to relate it with the Choi fidelity $\widetilde{F}_{\mathbb{O}_R}(\mathrm{id}_d) = \max_{\mathcal{M}\in\mathbb{O}_R} \left\langle \widetilde{J}_{\mathrm{id}_d}, \widetilde{J}_{\mathcal{M}} \right\rangle$, which is easier to compute. This is conceptually similar to an argument we made in the proof of Prop. 8.

Lemma 16. *For any unitary channel $\mathcal{U}$, it holds that $R_{\mathbb{O}}(\mathcal{U}) \geq F_{\mathbb{O}}(\mathcal{U})^{-1} \geq \widetilde{F}_{\mathbb{O}}(\mathcal{U})^{-1}$. In particular, if $R_{\mathbb{O}}(\mathcal{U}) = \widetilde{F}_{\mathbb{O}}(\mathcal{U})^{-1}$, then $F_{\mathbb{O}}(\mathcal{U}) = \widetilde{F}_{\mathbb{O}}(\mathcal{U})$.*

Proof. We have that

$$\begin{aligned} R_{\mathbb{O}}(\mathcal{U}) &= \max_{\psi} R_{\max}(\mathrm{id}\otimes\mathcal{U}(\psi)\|\mathrm{id}\otimes\mathcal{M}^\star(\psi)) \\ &\geq R_{\max}(\mathrm{id}\otimes\mathcal{U}(\psi^\star)\|\mathrm{id}\otimes\mathcal{M}^\star(\psi^\star)) \\ &\geq \left\langle \mathrm{id}\otimes\mathcal{U}(\psi^\star), \frac{\mathrm{id}\otimes\mathcal{U}(\psi^\star)}{F(\mathcal{U},\mathcal{M}^\star)} \right\rangle \\ &= F(\mathcal{U},\mathcal{M}^\star)^{-1} \\ &\geq F_{\mathbb{O}}(\mathcal{U})^{-1} \\ &\geq \widetilde{F}_{\mathbb{O}}(\mathcal{U})^{-1}. \end{aligned} \tag{85}$$

where in the first line we chose $\mathcal{M}^\star \in \mathbb{O}$ as an optimal channel for $R_{\mathbb{O}}$ in (9), in the second line we chose $\psi^\star$ as a state such that $F(\mathcal{U},\mathcal{M}^\star) = F(\mathrm{id}\otimes\mathcal{U}(\psi^\star), \mathrm{id}\otimes\mathcal{M}^\star(\psi^\star))$, in the third line we used that $\frac{\mathrm{id}\otimes\mathcal{U}(\psi^\star)}{F(\mathcal{U},\mathcal{M}^\star)}$ is a feasible solution for the dual form of $R_{\max}$ in (12), and the last line is by definition. ■

For the theory of no-signalling channels, a direct computation tells us that $\widetilde{F}_{\mathbb{O}_R}(\mathrm{id}_d) = \frac{1}{d^2}$. Coupled with the fact $R_{\mathbb{O}_R}(\mathrm{id}_d) = d^2$ [8], we obtain from Lem. 16 that $F_{\mathbb{O}_R}(\mathrm{id}_d) = \frac{1}{d^2}$. This can alternatively be seen by a direct calculation:

$$\begin{aligned} F_{\mathbb{O}_R}(\mathrm{id}_d) &= \min_{\psi} \max_{\mathcal{M}\in\mathbb{O}_R} \langle \psi, \mathrm{id}\otimes\mathcal{M}(\psi)\rangle \\ &= \min_{\{\lambda_i\},\{|i\rangle\}} \max_{\sigma} \sum_{i=0}^{d-1} \lambda_i^2 \langle i|\sigma|i\rangle \\ &= \min_{\{\lambda_i\},\{|i\rangle\}} \lambda_{\max}^2 \\ &= \frac{1}{d^2}, \end{aligned} \tag{86}$$

where the first line is due to Lem. 4, and in the second line we used that a bipartite pure state ψ allows for a Schmidt decomposition $|\psi\rangle = \sum_{i=0}^{d-1} \lambda_i |i\rangle |i\rangle$ where $\lambda_{\max} := \max_i \lambda_i$ in the third line. This implies in particular that $F_{\mathbb{O}_R}(\mathrm{id}_2^{\otimes n}) = \frac{1}{2^{2n}}$.

First, it is insightful to see what the bounds of Thm. 1(a) tell us about one-shot transformations $\mathcal{E} \to \mathrm{id}_d$. Here, the maximal fidelity achievable under no-signalling codes can be computed with an SDP [102]. We will also use the fact that $R_{\mathbb{O}_\mathrm{R}}$ has been computed analytically for some simple channels [7], and $W_{\mathbb{O}_\mathrm{R}}$ can also be computed in such cases.

For instance, for the depolarising channel $\mathcal{D}_p(\rho) = (1-p)\rho + p\frac{1}{d}$ we get $W_{\mathbb{O}_\mathrm{R}}(\mathcal{D}_p) = p$, and the robustness and weight-based bounds are actually equal: we have $\varepsilon \geq p(d^2-1)/d^2$. In fact, the bounds match the achievable fidelity, meaning that Thm. 1(a) quantifies the error in the one-shot transformation $\mathcal{D}_p \to \mathrm{id}_d$ under $\mathbb{S}_{\mathrm{NS}}$ *exactly*. On the other hand, channels such as the qubit dephasing channel $\mathcal{Z}_p(\rho) = (1-p)\rho + Z\rho Z$ or the amplitude damping channel $\mathcal{N}_\gamma$ have no constant channels in their support, and thus the weight bound becomes useless with $W_{\mathbb{O}_\mathrm{R}}(\mathcal{Z}_p) = W_{\mathbb{O}_\mathrm{R}}(\mathcal{N}_\gamma) = 0$. However, the robustness bound gives $\varepsilon \geq \frac{1}{2} - |p - \frac{1}{2}|$ for the dephasing channel and $\varepsilon \geq \frac{1}{4}(2+\gamma-2\sqrt{1-\gamma})$ for the amplitude damping channel, and these bounds, again, exactly equal the fidelity achievable under no-signalling assistance. Thus, we see that the error bounds of Thm. 1(a) can be tight in relevant cases. We refer to Fig. 4 of the main text for an explicit comparison.

We now apply our result to get insights into asymptotic capacity. From Thm. 3(a), we immediately obtain a strong converse bound for adaptive capacity as

$$Q_{\mathrm{NS,adap}}(\mathcal{E}) \leq Q^\dagger_{\mathrm{NS,adap}}(\mathcal{E}) \leq \frac{1}{2}\log R_{\mathbb{O}_R}(\mathcal{E}). \tag{87}$$

For the capacity with parallel protocols, we can use the asymptotic equipartition property of Ref. [7] which showed that $D^{\infty}_{\mathbb{O}_R}(\mathcal{E}) = I(\mathcal{E})$ [3], where $I(\mathcal{E})$ is the channel mutual information defined as $I(\mathcal{E}_{A'\to B}) \coloneqq \max_{\phi_{EA'}} I(\mathrm{id} \otimes \mathcal{E}_{A'\to B}(\phi_{EA'}))$ with $I(\mathrm{id} \otimes \mathcal{E}(\phi_{EA'}))$ being the mutual information between E and B. Combining this result with Thm. 3(b), we obtain

$$Q_{\mathrm{NS}}(\mathcal{E}) \leq Q^{\dagger}_{\mathrm{NS}}(\mathcal{E}) \leq \frac{1}{2} I(\mathcal{E}). \tag{88}$$

Together with the known achievable capacity $Q_{\mathrm{NS}}(\mathcal{E}) \geq \frac{1}{2} I(\mathcal{E})$ [89, 102], this ensures the strong converse property of no-signalling–assisted quantum capacity $Q_{\mathrm{NS}}(\mathcal{E}) = Q^{\dagger}_{\mathrm{NS}}(\mathcal{E}) = \frac{1}{2} I(\mathcal{E})$.

Importantly, no-signalling–assisted communication includes entanglement-assisted communication as a subclass, and thus the strong converse bound for the no-signalling–assisted capacity serves as that for the entanglement-assisted capacity. We note that the strong converse property for both settings was previously shown in several different ways [7, 8, 84, 92, 107, 108]; our general resource-theoretic approach provides an alternative perspective that employs the asymptotic equipartition property of Ref. [7] to show this important relation.

4.1.2. *PPT and separable assistance*

We now study another approach to quantum communication, where assistance by local operations and (two-way) classical communication is allowed. Since such operations are extremely difficult to characterise mathematically [109], various relaxations are frequently employed. A particularly useful set of operations, amenable to both an efficient numerical computations as well as a simplified analytical characterisation, is the set of PPT (positive partial transpose) codes [102, 110]. In the general case of bipartite channels $AB \to A'B'$, a map $\mathcal{M}$ is called PPT if the partial transpose of $J_{\mathcal{M}}$ across the $AA' : BB'$ bipartition is positive [110]. Analogously, a map is separable if $J_{\mathcal{M}}$ is a separable operator [111]. Quantum communication through a channel $\mathcal{E} : A \to B$ with PPT (or SEP) assistance is then defined as allowing Alice and Bob to perform joint PPT (or separable) operations between their successive channel uses (see e.g. [97]). The goal is then, again, to simulate a number of uses of the identity channel id_2. Using $\mathbb{O}_{\mathrm{PPT}}$ to denote the set of all PPT channels and analogously for $\mathbb{O}_{\mathrm{SEP}}$, for both classes of channels we have [110, 112]

$$\widetilde{F}_{\mathbb{O}_{\mathrm{PPT}}}(\mathrm{id}_d) = \widetilde{F}_{\mathbb{O}_{\mathrm{SEP}}}(\mathrm{id}_d) = \max_{\mathcal{M}\in\mathbb{O}_{\mathrm{SEP}}} \left\langle \widetilde{J}_{\mathrm{id}_d}, \widetilde{J}_{\mathcal{M}} \right\rangle = \frac{1}{d}. \tag{89}$$

Using now the fact that $R_{\mathbb{O}_{\mathrm{PPT}}}(\mathrm{id}_d) = R_{\mathbb{O}_{\mathrm{SEP}}}(\mathrm{id}_d) = d$ [11], from Lem. 16 we conclude that $F_{\mathbb{O}_{\mathrm{PPT}}}(\mathrm{id}_d) = F_{\mathbb{O}_{\mathrm{SEP}}}(\mathrm{id}_d) = \frac{1}{d}$, and in particular $F_{\mathbb{O}}(\mathrm{id}_2^{\otimes n}) = \frac{1}{2^n}$.

In order to describe these operations in our framework, one can notice that any PPT code, understood as a superchannel acting on a channel $\mathcal{E} : A \to B$, preserves the set of PPT channels [18, 19]. This motivates us to define the class of PPT-preserving superchannels: $\mathbb{S}_{\mathrm{PPT}} \coloneqq \left\{ \Theta \;\middle|\; \Theta(\mathcal{M}) \in \mathbb{O}_{\mathrm{PPT}}\ \forall \mathcal{M} \in \mathbb{O}_{\mathrm{PPT}} \right\}$. Any bound obtained for such superchannels will then upper bound the capabilities of PPT codes. Similarly, one defines $\mathbb{S}_{\mathrm{SEP}} \coloneqq \left\{ \Theta \;\middle|\; \Theta(\mathcal{M}) \in \mathbb{O}_{\mathrm{SEP}}\ \forall \mathcal{M} \in \mathbb{O}_{\mathrm{SEP}} \right\}$ as the separability-preserving superchannels. More general PPT- and separability-preserving quantum processes are defined analogously.

Using this approach, our results immediately provide several bounds on the capabilities of both PPT and separable codes in assisting quantum communication, both in the non-asymptotic and asymptotic settings. We discuss in particular the applications to upper bounding quantum capacity.

Denoting by $Q_{\mathrm{SEP,adap}}(\mathcal{E})$ the quantum capacity of a channel $\mathcal{E}$ assisted by general (adaptive, two-way) separable codes, Thm. 3(a) gives

$$Q_{\mathrm{SEP,adap}}(\mathcal{E}) \leq Q^{\dagger}_{\mathrm{SEP,adap}}(\mathcal{E}) \leq \log R_{\mathbb{O}_{\mathrm{SEP}}}(\mathcal{E}). \tag{90}$$

Here, $\log R_{\mathbb{O}_{\mathrm{SEP}}}$ is the so-called max-relative entropy of entanglement of a channel, and our result recovers exactly the strong converse bound of Ref. [16] (see below for a clarification regarding this quantity). Our approach then provides a remarkable simplification of the proof methods used to show this relation. Note that the quantity $R_{\mathbb{O}_{\mathrm{SEP}}}$ was recently also considered in Ref. [11] in the different context of quantifying the memory of a quantum channel, where it was evaluated for a number of cases, and in particular shown to be computable exactly for low-dimensional channels: as long as $d_A \leq 3$ and $d_B = 2$, we have $R_{\mathbb{O}_{\mathrm{SEP}}}(\mathcal{E}) = \max\{1, \|J_{\mathcal{E}}\|_\infty\}$. This provides an analytically computable strong converse bound for the capacities of all qubit channels.

---

[3] Although the asymptotic equipartition property was shown for the diamond norm smoothing, it can be straightforwardly extended to the fidelity smoothing using the relation between the diamond norm and fidelity [43, 46].

Similarly, for PPT codes we get

$$Q_{\mathrm{PPT,adap}}(\mathcal{E}) \leq Q^{\dagger}_{\mathrm{PPT,adap}}(\mathcal{E}) \leq \log R_{\mathbb{O}_{\mathrm{PPT}}}(\mathcal{E}). \tag{91}$$

This gives a general, SDP-computable bound on the quantum capacity and is similar to the results of Refs. [93, 96], although the robustness (max-relative entropy)-based quantities in those works optimise over a larger set of maps than our $R_{\mathbb{O}_{\mathrm{PPT}}}$. Specifically, following the observations in Refs. [19, 99], the precise result of Refs. [93, 96] can be recovered exactly in the framework of this work if one considers the class of *completely* PPT-preserving superchannels [18] instead of PPT-preserving superchannels $\mathbb{S}_{\mathrm{PPT}}$ as we have done here. We also note that $R_{\mathbb{O}_{\mathrm{PPT}}}(\mathcal{E}) = \max\{1, \|J_{\mathcal{E}}\|_\infty\}$ for any channel with $d_B = 2$ [11].

Of note is the fact that previous approaches to establishing such bounds typically relied on so-called amortised monotones [16, 96, 97, 113] defined at the level of quantum states, while our method is based on resource measures directly at the level of channels. Our results not only provide a streamlined approach to recovering the specialised bounds of Refs. [16, 96], but also reveal these approaches to be a part of a broad resource-theoretic framework. A notable technical difference between our approach and the previous methods is that we do not need to make any assumptions about the structure of the communication protocol, and in particular we do not need to impose that it is constructed by sequentially composing free channel transformations, as previous works did.

Let us also note that the generality of our methods allows them to be immediately applicable to related settings such as secret key agreement [114] (giving bounds on the private capacities and recovering a result of Ref. [16]) and bidirectional quantum communication [115] (giving bounds on quantum and private capacities, recovering results similar to Ref. [116]).

Before proceeding further, let us clarify a difference between our measures $R_{\mathbb{O}_{\mathrm{SEP}}}$, $R_{\mathbb{O}_{\mathrm{PPT}}}$ and related quantities found in literature. Max-relative entropy quantities for channels are often defined in the literature with respect to a set of quantum states, rather than trace-preserving maps, which might seem different from our definitions. For instance, the max-relative entropy of entanglement of a channel was originally defined as [16]

$$\widetilde{D}_{\mathrm{SEP}}(\mathcal{E}) := \max_{\psi} \min \left\{ \log \lambda \;\middle|\; \mathrm{id} \otimes \mathcal{E}(\psi) \leq \lambda \sigma,\ \sigma \in \mathbb{F}_{\mathrm{SEP}} \right\} \tag{92}$$

where $\sigma \in \mathbb{F}_{\mathrm{SEP}}$ optimises over all separable states — not necessarily valid Choi matrices — which can be understood as an optimisation over a cone of completely positive maps. However, it is not difficult to show that this definition is equal to ours.

Lemma 17. *Let $\mathbb{O}$ denote either $\mathbb{O}_{\mathrm{SEP}}$ or $\mathbb{O}_{\mathrm{PPT}}$, and define $\widetilde{\mathbb{O}}$ as the cone of separable operators on $A:B$ or PPT operators on $A:B$, respectively. Then, we can equivalently optimise over completely positive and trace non-increasing maps in the definition of the robustness $R_{\mathbb{O}}$. That is, for $\mathcal{E}: A \to B$ we have*

$$\begin{aligned} &R_{\mathbb{O}}(\mathcal{E}) \\ &= \min \left\{ \lambda \;\middle|\; J_{\mathcal{E}} \leq \lambda J_{\mathcal{M}},\ \mathcal{M} \in \widetilde{\mathbb{O}},\ \mathrm{Tr}_B J_{\mathcal{M}} = \mathbb{1} \right\} \\ &= \min \left\{ \lambda \;\middle|\; J_{\mathcal{E}} \leq \lambda J_{\widetilde{\mathcal{M}}},\ \widetilde{\mathcal{M}} \in \widetilde{\mathbb{O}},\ \mathrm{Tr}_B J_{\widetilde{\mathcal{M}}} \leq \mathbb{1} \right\} \\ &= \min \left\{ \left\|\mathrm{Tr}_B J_{\widetilde{\mathcal{M}}}\right\|_\infty \;\middle|\; J_{\mathcal{E}} \leq J_{\widetilde{\mathcal{M}}},\ \widetilde{\mathcal{M}} \in \widetilde{\mathbb{O}} \right\} \\ &= \max_{\psi} \min \left\{ \lambda \;\middle|\; \mathrm{id} \otimes \mathcal{E}(\psi) \leq \lambda J_{\widetilde{\mathcal{M}}},\ \widetilde{\mathcal{M}} \in \widetilde{\mathbb{O}},\ \mathrm{Tr}(J_{\widetilde{\mathcal{M}}}) = 1 \right\}. \end{aligned} \tag{93}$$

Proof. Let $R'_{\mathbb{O}}$ denote the quantity in the second line of (93). Clearly, $R_{\mathbb{O}}(\mathcal{E}) \geq R'_{\mathbb{O}}(\mathcal{E})$. To see the converse inequality, let $\widetilde{\mathcal{M}} \in \widetilde{\mathbb{O}}$ be an optimal map in the optimisation for $R'_{\mathbb{O}}$. By definition, we have that $C := \mathbb{1}_A - \mathrm{Tr}_B J_{\widetilde{\mathcal{M}}} \geq 0$. Define the completely positive and trace-preserving map $\mathcal{M}$ through $J_{\mathcal{M}} = J_{\widetilde{\mathcal{M}}} + C \otimes \frac{\mathbb{1}_B}{d_B}$. As $C \otimes \frac{\mathbb{1}_B}{d_B} \in \widetilde{\mathbb{O}}$, convexity of $\widetilde{\mathbb{O}}$ gives that $\mathcal{M} \in \widetilde{\mathbb{O}} \cap \mathrm{CPTP}$. This means that $J_{\mathcal{E}} \leq R'_{\mathbb{O}}(\mathcal{E}) J_{\widetilde{\mathcal{M}}} \leq R'_{\mathbb{O}}(\mathcal{E}) J_{\mathcal{M}}$, hence $R_{\mathbb{O}}(\mathcal{E}) \leq R'_{\mathbb{O}}(\mathcal{E})$ and so the two quantities must be equal. The third line is a simple restatement of the second, and the fourth equality has been previously shown in Ref. [96, Lemma 7]. ■

This result shows a bound of Ref. [16, Thm. 5.2] (also [117, Prop. 1]) to be tight.

In the formalism of 'resource-generating power' found in Refs. [3, 4, 20], the result can be understood as the fact that the entanglement-generating power of a channel $\mathcal{E}: A \to B$ in terms of the generalised robustness of entanglement $R_{\mathbb{F}_{\mathrm{SEP}}}$ is equal to the robustness $R_{\mathbb{O}_{\mathrm{SEP}}}(\mathcal{E})$.

4.2. Quantum gate synthesis and magic states

To realise reliable computation under the presence of noise, it is essential to encode quantum states into higher-dimensional spaces by a quantum error correcting code and carry out computation within the logical subspace in a fault-tolerant manner [118]. Among the whole class of quantum gates, Clifford gates are known to be easier to implement on many codes of interest [119–123] whereas logical implementation of non-Clifford gates results in a large overhead cost [124, 125]. The cost is often quantified in terms of the T gates, with T being the single-qubit unitary $T = \text{diag}(1, e^{i\pi/4})$ [125] which enables universal quantum computation together with Clifford gates and can be considered as the most difficult to implement. In particular, any circuit composed of only Clifford gates can be efficiently simulated on a classical computer [121], but a circuit built with the Clifford+T gate set has a simulation time exponential in the number of T gates [126]. Much effort has therefore been devoted to studying the optimal ways to realise circuits with minimal T-gate cost [127–129].

However, choices of relevant operations other than the commonly used set of Clifford+T gates can be made. Indeed, the Clifford unitaries are not the only types of channels that admit efficient classical simulation schemes: this holds true also for stabiliser operations [130] and an even larger class of completely stabiliser-preserving channels [26]. Such extensions have shown potential to significantly improve the cost of implementing quantum circuits in terms of the T-gate count [131–133] as well as the simulation cost of noisy circuits [60, 85, 130, 134–136]. It is therefore of interest to understand the limitations on manipulating quantum circuits through the most general means.

The task of synthesising gates and circuits is often realised through magic state distillation [125], which aims to prepare clean magic states — states that cannot be obtained with stabiliser operations alone — and use them to implement costly quantum gates through the scheme of state injection [124]. Magic state distillation can provide feasible ways to synthesise gates and circuits [129], and investigating the precise relations between distillation and gate synthesis is highly important in paving the way to fault-tolerant quantum computation [129, 137, 138].

The resource theory of magic was thus introduced to understand the limitations of manipulating and distilling states using different free operations [26, 130, 136]. We can apply our general results to this formalism to obtain fundamental limitations on both magic state distillation and more general gate manipulation protocols which act the level of quantum channels directly. In this resource theory, stabiliser operations $\mathbb{O}_{\text{STAB}}$ are built through Clifford gates, Pauli measurements, and preparations of ancillary states in the computational basis [130]. Then, the set of stabiliser states $\mathbb{F}_{\text{STAB}}$ is defined to be the states that can be created by such operations, which coincides with the convex hull of eigenstates of Pauli operators. The larger set of completely stabiliser-preserving operations [26] is defined as all channels whose Choi state satisfies $\widetilde{J}_{\mathcal{E}} \in \mathbb{F}_{\text{STAB}}$, which allows the computation of quantifiers such as $R_{\mathbb{O}}$ and $W_{\mathbb{O}}$ as semidefinite programs (although their size scales superexponentially with the number of qubits). By choosing this set as our free operations $\mathbb{O}$, we ensure that all of our bounds will apply also to smaller sets of transformations, which includes all channels typically considered in the study of magic. The channel manipulation protocols $\mathbb{S}$ that we consider include, in particular, any pre- and post-processing of the channel with other completely stabiliser-preserving channels.

Of particular importance are the channels which can be realised through state injection, such as unitary gates from the third level of the Clifford hierarchy C_3 [124], that is, unitaries which map Pauli operators to Clifford gates. This includes many gates of practical relevance such as the T gate, the controlled-phase gate CS, the controlled-controlled-Z gate CCZ, or the Toffoli gate. All channels which can be implemented in this way obey the following relation, which we will prove and generalise in the next section.

Lemma 18. *Let $\mathcal{N} : A \to B$ be any channel which can be implemented by state injection, that is, for which there exists a free state $\tau \in \mathbb{F}_{\text{STAB}}(R \otimes A)$ and free operation $\mathcal{G} \in \mathbb{O}$ such that the state $\omega = \text{id}_R \otimes \mathcal{N}(\tau)$ allows for the implementation of the channel $\mathcal{N}$ as $\mathcal{N}(\rho) = \mathcal{G}(\rho \otimes \omega)\ \forall \rho$. Then*

$$\begin{aligned} R_{\mathbb{O}}(\mathcal{N}) &= R_{\mathbb{F}_{\text{STAB}}}(\omega) \\ W_{\mathbb{O}}(\mathcal{N}) &= W_{\mathbb{F}_{\text{STAB}}}(\omega) \\ F_{\mathbb{O}}(\mathcal{N}) &= F_{\mathbb{F}_{\text{STAB}}}(\omega). \end{aligned} \tag{94}$$

That is, the channel resource measures all reduce to the corresponding state-based resource measures of the associated state $\omega = \text{id} \otimes \mathcal{N}(\tau)$.

The Lemma can be considered as an application of the idea of resource simulability [27, 97], which generalises the notions of teleportation-based simulation from entanglement theory [15, 74, 100]. Importantly, this result is valid for all unitary channels $\mathcal{U}(\cdot) = U \cdot U^\dagger$ where U is a k-qubit unitary $U \in C_3$ and τ is the $2k$-qubit maximally entangled state [124]. When U is additionally a diagonal gate, the state injection can be performed more easily with the state $\omega = U\, |+\rangle\langle +|^{\otimes k}\, U^\dagger$. Notably, when U is a 1-, 2-, or 3-qubit diagonal unitary in C_3, then the quantities $R_{\mathbb{O}}(\mathcal{U})$ and $F_{\mathbb{O}}(\mathcal{U})$ become multiplicative, owing to the multiplicativity of the associated state-based monotones [60, 85]. We will hereafter restrict our discussion to diagonal unitaries $U \in C_3$ of up to 3 qubits for simplicity, as this is enough to encompass most gates of practical interest (or gates Clifford-equivalent thereto). We then use $|U\rangle := U\, |+\rangle^{\otimes k}$ for the corresponding states. An interesting property of Lem. 18 for

such gates is that the result actually does not depend on the choice of free operations, meaning that the robustness, weight, and fidelity measures all have the same values for any set of channels $\mathbb{O}$ which can implement state injection gadgets — this ranges from the practically relevant stabiliser operations to all stabiliser-preserving channels [26].

Let us now look at how our main results can be understood in this setting. In general, the bound of Thm. 13 provides insight into the best achievable performance of any free channel transformation protocol; namely, for any transformation $U \to U'$ which takes n uses of a diagonal gate $U \in C_3$ to m copies of a target diagonal gate $U' \in C_3$, it holds that

$$\frac{n}{m} \geq \frac{\log R_{\mathbb{F}_{\rm STAB}}(|U'\rangle\langle U'|)}{\log R_{\mathbb{F}_{\rm STAB}}(|U\rangle\langle U|)}. \tag{95}$$

For instance, using the known values of $R_{\mathbb{F}_{\rm STAB}}(|T\rangle\langle T|)$ and $R_{\mathbb{F}_{\rm STAB}}(|CCZ\rangle\langle CCZ|)$ [85] we conclude that $n \geq 3.6335$ and so 4 T gates are necessary to perfectly synthesise a CCZ gate — this is a slight strengthening of previous results which showed this optimality in other settings [136, 139], as we establish that even the most general, adaptive channel manipulation protocols cannot do better.

In practice, the input gate might be affected by noise, and similarly the output of the protocol might only be required to be a good approximation of the target gate up to small errors. We can then apply Thm. 2 to show that, for any channel $\mathcal{E}$ and any deterministic gate synthesis protocol Υ which transforms n uses of a noisy channel $\mathcal{E}$ to m copies of the target unitary channel $\mathcal{U}(\cdot) = U \cdot U^\dagger$ up to accuracy $F(\Upsilon(\mathcal{E},\ldots,\mathcal{E}), \mathcal{U}^{\otimes m}) \geq 1-\varepsilon$, it holds that

$$\begin{aligned} n &\geq \log_{1/W_{\mathbb{O}}(\mathcal{E})} \frac{1-F_{\mathbb{F}_{\rm STAB}}(U)^m}{\varepsilon}, \\ n &\geq \log_{R_{\mathbb{O}}(\mathcal{E})} \frac{1-\varepsilon}{F_{\mathbb{F}_{\rm STAB}}(U)^m}. \end{aligned} \tag{96}$$

We stress that the coefficient $F_{\mathbb{F}_{\rm STAB}}(U) = \max_{\sigma\in\mathbb{F}_{\rm STAB}} \langle U|\sigma|U\rangle$ is the stabiliser fidelity [85] of the associated state $|U\rangle$, which is known exactly for most gates of interest [85, 139]: for instance, $F_{\mathbb{F}_{\rm STAB}}(T) = \frac{1}{4}(2+\sqrt{2})$, $F_{\mathbb{F}_{\rm STAB}}(CCZ) = \frac{9}{16}$. The bounds thus establish universal, efficiently computable restrictions on gate synthesis protocols, providing in particular a fundamental lower bound on the associated resource cost that any physical protocol must satisfy. Considering the previous example of the transformation $T \to CCZ$, we obtain $n > 3$ for error values up to $\varepsilon \approx 0.095$, showing that a large error is necessary if one employs fewer than 4 T gates in the transformation.

As a special case, Thm. 2 gives also fundamental restrictions on the resource cost of magic state distillation protocols. In particular, for any protocol $\mathcal{M} \in \mathbb{O}$ which satisfies $F(\mathcal{M}(\rho^{\otimes n}), |U\rangle\langle U|^{\otimes m}) \geq 1-\varepsilon$, we necessarily have

$$\begin{aligned} n &\geq \log_{1/W_{\mathbb{F}}(\rho)} \frac{1-F_{\mathbb{F}_{\rm STAB}}(U)^m}{\varepsilon}, \\ n &\geq \log_{R_{\mathbb{O}}(\rho)} \frac{1-\varepsilon}{F_{\mathbb{F}_{\rm STAB}}(U)^m}. \end{aligned} \tag{97}$$

Our weight-based bound improves on the previous bound of Ref. [75] and extends its applicability beyond full-rank input states. An explicit comparison in Fig. 3 of the main text reveals that this improvement can be substantial. We note that the robustness bound previously appeared in Ref. [60] in this context.

Moreover, from Thm. 3(a) we obtain a strong converse bound on all transformations of channels as

$$r^\dagger_{\rm adap}(\mathcal{E} \to \mathcal{U}) \leq \frac{\log R_{\mathbb{O}}(\mathcal{E})}{\log F_{\mathbb{O}}(U)^{-1}} \tag{98}$$

for any diagonal $U \in C_3$ of up to 3 qubits. This establishes limitations on the capabilities of general adaptive protocols in gate synthesis in the asymptotic limit.

We now consider some explicit examples to compare the different bounds. In the examples below, we compute the robustness $R_{\mathbb{O}}$ and weight $W_{\mathbb{O}}$ with the choice of $\mathbb{O}$ as the completely stabiliser-preserving operations. We refer to Fig. 3 of the main text.

First, we compare the bounds on the error incurred in one-shot transformations, as stated in Thm. 1(a) and Cor. 10. In Fig. 3**a**, our aim is to transform the noisy T gate $\mathcal{N}_p = \mathcal{D}_p \circ \mathcal{T}$ to the CCZ gate, where $\mathcal{D}_p(\rho) = (1-p)\rho + p\frac{\mathbb{1}}{2}$ is the depolarising channel. Notably, we see that the robustness bound indicates a significant error also in the noiseless case ($p = 0$), whereas the weight bound becomes trivial for noiseless inputs. In Fig. 3**b**, we study the error incurred in distilling the $|CCZ\rangle$ state from three copies of the noisy T state $\rho_p = \mathcal{D}_p(|T\rangle\langle T|)$. The result explicitly shows the substantial improvement of our bounds over the bounds of Ref. [75], which become nearly 0 in this case. We also see the previously remarked fact that a significant (≈ 0.1) error is unavoidable in the conversion $|T\rangle^{\otimes 3} \to |CCZ\rangle$.

When considering the distillation of noisy resources, noise applied at the level of channels can affect the results in different ways than noise applied at the level of states. For example, in subfigures **c–d** we present a comparison between the bounds for magic state distillation from the noisy T state ρ_p and for gate synthesis from the noisy T gate $\mathcal{N}_p$. The result shows a difference between the gate synthesis properties of the two: we can see that the bounds impose much higher requirements on the number

of noisy states required to succeed. The comparatively weaker bound on the required copies of the noisy T gate indicates a possibility that, as long as a certain type of noise is fixed (e.g. depolarising noise here), manipulating the noisy gate at the level of channels might offer an improvement over methods which rely on distilling $|T\rangle$ states from the noisy state ρ_p.

The difference between the state and channel cases can be understood as follows. Letting $\mathcal{G}$ be the standard state injection gadget such that $\mathcal{G}(\cdot \otimes |T\rangle\langle T|) = \mathcal{T}(\cdot)$ [124], one can easily verify that $\mathcal{G}(\cdot \otimes \frac{\mathbb{1}}{2})$ is the completely *dephasing* channel $\mathcal{Z}_{1/2}(\rho) = \frac{1}{2}\rho + \frac{1}{2}Z\rho Z$. This means that the state ρ_p is equivalent to the channel $\mathcal{N}_p^{\mathcal{Z}} = (1-p)\mathcal{T} + p\mathcal{Z}_{1/2}$, and thus depolarising noise at the level of states corresponds to dephasing, rather than depolarising, at the level of channels. In particular, Lem. 18 gives $R_{\mathbb{O}}(\mathcal{N}_p^{\mathcal{Z}}) = R_{\mathbb{F}}(\rho_p)$ and analogously for the other quantities.

4.2.1. Remarks on the case of qudits

Our results apply in an analogous way to the resource theory of magic for qudit systems of prime dimension [130]. Here, our robustness-based bounds can be used to recover results related to Refs. [27, 86], although these works considered a slightly different approach based on the discrete Wigner function and associated norms. The technical differences in the framework of Ref. [86], and in particular the use of sub-normalised operators rather than normalised quantum states, mean that these results do not exactly fit within our framework. Nevertheless, we can use them to get insights about important states in this theory.

In particular, in Table I of the main text we reported the value of $F_{\mathbb{F}}$ (and analogously $F_{\mathbb{O}}$) for a selection of quantum states, and in particular the H_+ state and the Norrell state. These results are obtained by leveraging the findings of Ref. [86]: for the aforementioned states, Ref. [86] showed that $\log R_{\mathbb{F}}(\psi)$ (max-relative entropy of magic) equals a quantity called the 'min-thauma' $\theta_{\min}(\psi)$. But in general it holds that

$$R_{\mathbb{F}}(\psi) \geq F_{\mathbb{F}}(\psi)^{-1} \geq 2^{\theta_{\min}(\psi)}, \tag{99}$$

where the first inequality holds for any pure state [66, 140] (cf. Lem. 16), and the second is a consequence of the definition of $\theta_{\min}$ [86]. Therefore, whenever $\theta_{\min}(\psi) = \log R_{\mathbb{F}}(\psi)$, we conclude that also $\log F_{\mathbb{F}}(\psi)$ has the same value.

For channels and states whose value of $F_{\mathbb{O}}$ or $F_{\mathbb{F}}$ has not been established or is not known to be multiplicative (such as the qutrit T state), one can instead substitute $F_{\mathbb{F}}$ with $2^{-\theta_{\min}}$ in all of our bounds. In particular, since $\theta_{\min}$ is additive on tensor products [86], the many-copy results of Thms. 3(a) and 3(b) can always be applied by choosing $G_{\mathbb{F}}(\phi) = 2^{-\theta_{\min}(\phi)}$.

4.3. Proof of Lem. 18 and extension to other resources

The basic idea behind Lem. 18 is to exploit the fact that some channels can be reversibly interconverted with state resources through free operations, which means that the two types of resources can be considered as equivalent. This idea was first applied in the theory of entanglement [100, Sec. 5], later extended to general entanglement manipulation protocols [15] and to other types of resource theories [17, 97]. Within the theory of magic states, the idea can be understood as a generalisation of gate teleportation [124].

A general formulation of this property is as follows (see also [17, Sec. VI]).

Lemma 19. *Let $\mathbb{O}$ be any class of free operations which can prepare all free states $\mathbb{F}$, that is, $\mathcal{R}_\sigma \in \mathbb{O}\ \forall \sigma \in \mathbb{F}$. Let $\mathcal{N} : A \to B$ be any channel such that:*

(i) there exists a free superchannel $\Gamma \in \mathbb{S}$ and a state ω such that $\Gamma(\mathcal{R}_\omega) = \mathcal{N}$,

(ii) there exists a free superchannel $\Theta \in \mathbb{S}$ such that $\Theta(\mathcal{N}) = \mathcal{R}_\omega$.

Then

$$\begin{aligned} R_{\mathbb{O}}(\mathcal{N}) &= R_{\mathbb{F}}(\omega) \\ W_{\mathbb{O}}(\mathcal{N}) &= W_{\mathbb{F}}(\omega) \\ F_{\mathbb{O}}(\mathcal{N}) &= F_{\mathbb{F}}(\omega). \end{aligned} \tag{100}$$

Proof. Follows directly from the monotonicity of the measures $R_{\mathbb{O}}$, $W_{\mathbb{O}}$, $F_{\mathbb{O}}$ under free transformations $\mathbb{S}$, coupled with Lem. 7. ■

In practice, the superchannel Γ is often realised as a state injection protocol which provides ω as an ancillary system and processes the joint state with a free operation in $\mathbb{O}$. This is the way in which this property is usually applied both in entanglement theory [15, 74, 100] and in more general settings [17, 97]. For completeness, we provide a statement of the property in this form (see also [27]).

Lemma 20. *Consider any resource theory such that $\sigma, \sigma' \in \mathbb{F} \Rightarrow \sigma \otimes \sigma' \in \mathbb{F}$ and let $\mathbb{O}$ be a chosen class of free operations. Let $\mathcal{N} : A \to B$ be any channel such that:*

(i) it can be implemented through state injection, that is, there exists a free operation $\mathcal{G} \in \mathbb{O}(AC \to B)$ and a state $\omega \in \mathbb{D}(C)$ such that $\mathcal{G}(\cdot \otimes \omega) = \mathcal{N}(\cdot)$,

(ii) there exists a free transformation $\Theta \in \mathbb{S}$ such that $\Theta(\mathcal{N}) = \mathcal{R}_\omega$; for example, $\mathcal{N}(\tau) = \omega$ for some $\tau \in \mathbb{F}$, or $\mathrm{id} \otimes \mathcal{N}(\tau) = \omega$ when $\mathcal{M} \in \mathbb{O} \Rightarrow \mathrm{id} \otimes \mathcal{M} \in \mathbb{O}$.

Then

$$\begin{aligned} R_{\mathbb{O}}(\mathcal{N}) &= R_{\mathbb{F}}(\omega) \\ W_{\mathbb{O}}(\mathcal{N}) &= W_{\mathbb{F}}(\omega) \\ F_{\mathbb{O}}(\mathcal{N}) &= F_{\mathbb{F}}(\omega). \end{aligned} \tag{101}$$

Remark. In particular, the result holds in the resource theory of magic for any k-qubit unitary channel $\mathcal{U}(\cdot) = U \cdot U^\dagger$ from the third level of the Clifford hierarchy [124]. In this case: $\mathbb{O}$ can be any subset of completely stabiliser-preserving operations which allows for the implementation of state injection gadgets; ω is given by the Choi state $(\mathbb{1} \otimes U) |\psi^+\rangle\langle\psi^+| (\mathbb{1} \otimes U^\dagger)$ where $|\psi^+\rangle$ is the $2k$-qubit maximally entangled state; and the free superchannel Θ consist of simply preparing the free state $|\psi^+\rangle\langle\psi^+|$ before the application of $\mathcal{U}$. In the case of diagonal gates in the third level of the Clifford hierarchy, we can use $\omega = U |+\rangle\langle+|^{\otimes k} U^\dagger$ and the result is valid for the larger class of stabiliser-preserving operations. A similar state injection protocol can also be employed in qudit systems with odd prime dimensions as well [141, 142].

Proof. The first part of the proof can be shown following [27, Prop. 22]:

$$\begin{aligned} R_{\mathbb{O}}(\mathcal{N}) &= \min_{\mathcal{M} \in \mathbb{O}} R_{\max}\left(\mathcal{N} \| \mathcal{M}\right) \\ &= \min_{\mathcal{M} \in \mathbb{O}} R_{\max}\left(\mathcal{G}(\cdot \otimes \omega) \,\middle\|\, \mathcal{M}\right) \\ &\leq \min_{\sigma \in \mathbb{F}} R_{\max}\left(\mathcal{G}(\cdot \otimes \omega) \,\middle\|\, \mathcal{G}(\cdot \otimes \sigma)\right) \\ &\leq \min_{\sigma \in \mathbb{F}} R_{\max}\left([\cdot \otimes \omega] \,\middle\|\, [\cdot \otimes \sigma]\right) \\ &= \min_{\sigma \in \mathbb{F}} \max_{\psi_{RA}} R_{\max}\left(\psi \otimes \omega \,\middle\|\, \psi \otimes \sigma\right) \\ &= \min_{\sigma \in \mathbb{F}} R_{\max}\left(\omega \,\middle\|\, \sigma\right) \\ &= R_{\mathbb{F}}(\omega), \end{aligned} \tag{102}$$

where the first inequality is since $\mathcal{G}(\cdot \otimes \sigma) \in \mathbb{O}$ for any $\sigma \in \mathbb{F}$, the second inequality is by the data processing inequality of $R_{\max}(\mathcal{E} \| \mathcal{F})$ [67], and the equality in the second-to-last line is by the data processing inequality of $R_{\max}(\rho \| \sigma)$ [68]. On the other hand, using the monotonicity of $R_{\mathbb{O}}$ under free superchannels, we have

$$R_{\mathbb{O}}(\mathcal{N}) \geq R_{\mathbb{O}}(\Theta(\mathcal{N})) = R_{\mathbb{O}}(\mathcal{R}_\omega) \geq R_{\mathbb{F}}(\omega) \tag{103}$$

where the last inequality is by Lem. 7. The cases of $W_{\mathbb{O}}$ and $F_{\mathbb{O}}$ proceed in the same way. ■

An interesting consequence of such an operational equivalence of channels $\mathcal{N}$ and states ω is that it allows us to simplify general, adaptive channel manipulation protocols into simply acting on n copies of the state ω through the use of so-called teleportation stretching [15] (see also [97]).

Supplementary Note 5: Extension to probabilistic protocols

We now consider probabilistic sub-superchannels that need not preserve quantum channels, but can instead map them to their probabilistic implementations — completely positive and trace–non-increasing maps. Recall that the set of free subchannels $\tilde{\mathbb{O}}$ with respect to the given set of free channels $\mathbb{O}$ is given by

$$\tilde{\mathbb{O}} := \left\{ \tilde{\mathcal{M}} \,\middle|\, \forall \rho \in \mathbb{D},\ \exists \mathcal{M} \in \mathbb{O}, t \in [0,1] \ \text{ s.t. } \mathrm{id} \otimes \tilde{\mathcal{M}}(\rho) = t \cdot \mathrm{id} \otimes \mathcal{M}(\rho) \right\}, \tag{104}$$

and the corresponding set of sub-superchannels is $\tilde{\mathbb{S}} := \left\{\tilde{\Theta} \,\middle|\, \forall \mathcal{M} \in \mathbb{O},\ \tilde{\Theta}(\mathcal{M}) \in \tilde{\mathbb{O}}\right\}$. Our figure of merit in probabilistic channel transformations is then the conditioned fidelity

$$F_{\rm cond}(\mathcal{U}, \tilde{\Theta}(\mathcal{E})) := \min_{\psi} F\left(\mathrm{id} \otimes \mathcal{U}(\psi), \frac{\mathrm{id} \otimes \tilde{\Theta}(\mathcal{E})(\psi)}{p(\psi)}\right) \tag{105}$$

where $p(\psi) = \mathrm{Tr}[\mathrm{id} \otimes \tilde{\Theta}(\mathcal{E})(\psi)]$.

It is instructive to see how the above notions translate into that for state theories. By taking preparation channels as free channels and relabeling $\mathbb{O} \to \mathbb{F}$ and $\mathbb{S} \to \mathbb{O}$, Eq. (104) reduces to $\tilde{\mathbb{F}} := \left\{\tilde{\sigma} \,\middle|\, \tilde{\sigma} \in \mathrm{cone}(\mathbb{F}), \mathrm{Tr}[\tilde{\sigma}] \leq 1\right\}$ and correspondingly we get $\tilde{\mathbb{O}} := \left\{\tilde{\mathcal{M}} \,\middle|\, \forall \sigma \in \mathbb{F},\ \tilde{\mathcal{M}}(\sigma) \in \tilde{\mathbb{F}}\right\}$. As for the conditional fidelity, since our free superchannels transform preparation channels to preparation channels, we replace the target unitary with target preparation channel that prepares target state ϕ. Writing $\tilde{\mathcal{M}}(\rho) = p\eta,\ \eta \in \mathbb{D}$, the conditional fidelity reduces to much clearer form $F_{\rm cond}(\phi, \tilde{\mathcal{M}}(\rho)) = F(\phi, \eta)$.

With this, we are now ready to present an extension of Thm. 1(a) as follows.

Theorem 21. *Suppose a free sub-superchannel $\tilde{\Theta} \in \tilde{\mathbb{S}}$ achieves $F_{\rm cond}(\tilde{\Theta}(\mathcal{E}), \mathcal{U}) \geq 1 - \varepsilon$ for some resourceful unitary channel $\mathcal{U}$. Then, if $\tilde{\Theta}$ occurs with input channel $\mathcal{E}$ and input state ψ at probability $p = \mathrm{Tr}[\mathrm{id} \otimes \tilde{\Theta}(\mathcal{E})(\psi)]$, it holds that*

$$\varepsilon \geq 1 - \frac{R_{\mathbb{O}}(\mathcal{E})\, F_{\mathbb{O}}^{\psi}(\mathcal{U})}{p} \tag{106}$$

and

$$\varepsilon \geq 1 - \frac{1 - (1 - F_{\mathbb{O}}^{\psi}(\mathcal{U})) W_{\mathbb{O}}(\mathcal{E})}{p} \tag{107}$$

where $F_{\mathbb{O}}^{\psi}(\mathcal{U}) := \max_{\mathcal{M} \in \mathbb{O}} F(\mathrm{id} \otimes \mathcal{U}(\psi), \mathrm{id} \otimes \mathcal{M}(\psi))$.
Alternatively, taking $\mathcal{M} \in \mathbb{O}$ to be a free channel such that $J_{\mathcal{E}} \geq W_{\mathbb{O}}(\mathcal{E}) J_{\mathcal{M}}$, it holds that

$$\varepsilon \geq (1 - F_{\mathbb{O}}^{\psi}(\mathcal{U}))\, \frac{W_{\mathbb{O}}(\mathcal{E})\, \mathrm{Tr}[(\mathrm{id} \otimes \tilde{\Theta}(\mathcal{M})(\psi)]}{p} \tag{108}$$

We also obtain corresponding bounds for state-based theories.

Proposition 22. *If there exists a free subchannel $\tilde{\mathcal{M}} \in \tilde{\mathbb{O}}$ such that $\tilde{\mathcal{M}}(\rho) = p\eta$ with $F(\eta, \phi) \geq 1 - \varepsilon$ for some resourceful pure state ϕ. Then, it holds that*

$$\varepsilon \geq 1 - \frac{R_{\mathbb{F}}(\rho)\, F_{\mathbb{F}}(\phi)}{p} \tag{109}$$

and

$$\varepsilon \geq (1 - F_{\mathbb{F}}(\phi)) \left(1 - \frac{1 - W_{\mathbb{F}}(\rho)}{p}\right). \tag{110}$$

Alternatively, taking $\sigma \in \mathbb{F}$ to be a free state such that $\rho \geq W_{\mathbb{F}}(\rho)\sigma$, it holds that

$$\varepsilon \geq (1 - F_{\mathbb{F}}(\phi))\, \frac{W_{\mathbb{F}}(\rho)\, \mathrm{Tr}[\tilde{\mathcal{M}}(\sigma)]}{p}. \tag{111}$$

It can be easily seen that when $\tilde{\Theta}$ is a superchannel, $p = 1$ holds for any ψ and the bounds of Thm. 21 reproduce Thm. 1(a). An interesting question is whether the no-go statement implied by Thm. 1(a), which says that perfect purification with $\varepsilon = 0$ is impossible for any channel with $W_{\mathbb{O}}(\mathcal{E}) > 0$, remains valid in probabilistic cases. For the choice of $\mathcal{M}$ as an optimal channel satisfying $J_{\mathcal{E}} \geq W_{\mathbb{O}}(\mathcal{E}) J_{\mathcal{M}}$, Eq. (108) (and (111) for state theories) implies that *if* $\mathrm{Tr}[\mathrm{id} \otimes \tilde{\Theta}(\mathcal{M})(\psi)] > 0$, the no-go theorem still holds. On the other hand, if $\mathrm{Tr}[\mathrm{id} \otimes \tilde{\Theta}(\mathcal{M})(\psi)] = 0$, meaning that the free part of $\mathcal{E}$ is completely cut off by the selective operation $\tilde{\Theta}$, then this does not give us any insight into ε. This is actually a natural consequence because such a perfect purification *is* indeed possible, implying that one cannot expect a bound in which $\varepsilon > 0$ for $W_{\mathbb{O}}(\mathcal{E}) > 0$ (see also [143]).

As an illustrative example, let us consider a state theory, in particular, the theory of coherence where $\mathbb{F}$ is the set $\mathrm{conv}\{|0\rangle\langle 0|, |1\rangle\langle 1|, |2\rangle\langle 2|\}$ defined on a three-dimensional system. Take $\rho = (1/2)\,|0\rangle\langle 0| + (1/2)\,|+_{12}\rangle\langle +_{12}|$ where $|+_{12}\rangle = (1/\sqrt{2})(|1\rangle + |2\rangle)$. Consider a free subchannel $\tilde{\mathcal{M}}(\cdot) = P_{12} \cdot P_{12}$ where $P_{12} := |1\rangle\langle 1| + |2\rangle\langle 2|$ is the projector onto the subspace

spanned by $\{|1\rangle, |2\rangle\}$. Then we get $\tilde{\mathcal{M}}(\rho) = (1/2)\,|+_{12}\rangle\langle +_{12}|$, indicating that we can obtain a pure state $|+_{12}\rangle$ with probability 1/2, although $W_{\mathbb{F}}(\rho) = 1/2 > 0$. This shows a clear contrast to the deterministic case of Thm. 1(a) and Cor. 10 as well as the probabilistic bounds shown in Ref. [75], which showed that perfect purification is impossible even probabilistically for full-rank states.

Another interesting observation from Thm. 21 is that unlike Eqs. (106) and (107) where the lower bounds for error decrease as success probability decreases, Eq. (108) appears to have an opposite behaviour with respect to the success probability. In fact, there is an intricate trade-off between the probability of detecting the free component $\mathrm{Tr}[\mathrm{id}\otimes\tilde{\Theta}(\mathcal{M})(\psi)]$ and success probability p, and this provides a practical instruction that to accomplish a purification protocol with high accuracy, it is crucial to reduce the probability of detecting the free part of the given channel, as characterised by the resource weight $W_{\mathbb{O}}$.

Proof of Thm. 21. Define $|U_\psi\rangle := \mathbb{1}\otimes U\,|\psi\rangle$ and let $\mathcal{M}\in\mathbb{O}$ be a channel that satisfies $J_{\mathcal{E}} \leq R_{\mathbb{O}}(\mathcal{E}) J_{\mathcal{M}}$. Then, by (105) we get

$$
\begin{aligned}
p(1-\varepsilon) &\leq \langle U_\psi|\mathrm{id}\otimes\tilde{\Theta}(\mathcal{E})(\psi)|U_\psi\rangle\\
&\leq R_{\mathbb{O}}(\mathcal{E})\,\langle U_\psi|\mathrm{id}\otimes\tilde{\Theta}(\mathcal{M})(\psi)|U_\psi\rangle\\
&\leq R_{\mathbb{O}}(\mathcal{E}) F_{\mathbb{O}}^{\psi}(\mathcal{U}).
\end{aligned}
\tag{112}
$$

The last line is because

$$
\begin{aligned}
\langle U_\psi|\mathrm{id}\otimes\tilde{\Theta}(\mathcal{M})(\psi)|U_\psi\rangle &= t\,\langle U_\psi|\mathrm{id}\otimes\mathcal{M}'(\psi)|U_\psi\rangle\\
&\leq t F_{\mathbb{O}}^{\psi}(\mathcal{U}) \leq F_{\mathbb{O}}^{\psi}(\mathcal{U})
\end{aligned}
\tag{113}
$$

where we used that $\tilde{\Theta}(\mathcal{M})\in\tilde{\mathbb{O}}$, ensuring by (104) that there exist $\mathcal{M}'\in\mathbb{O}$ and $t\in[0,1]$ such that

$$
\mathrm{id}\otimes\tilde{\Theta}(\mathcal{M})(\psi) = t\cdot\mathrm{id}\otimes\mathcal{M}'(\psi).
\tag{114}
$$

To show the second bound, let us redefine $\mathcal{M},\mathcal{N}$ as the channels satisfying $\mathcal{E} = W_{\mathbb{O}}(\mathcal{E})\mathcal{M} + (1-W_{\mathbb{O}}(\mathcal{E}))\mathcal{N},\ \mathcal{M}\in\mathbb{O}$. Then,

$$
\begin{aligned}
p(1-\varepsilon) &\leq \langle U_\psi|\mathrm{id}\otimes\tilde{\Theta}(\mathcal{E})(\psi)|U_\psi\rangle\\
&= W_{\mathbb{O}}(\mathcal{E})\,\langle U_\psi|\mathrm{id}\otimes\tilde{\Theta}(\mathcal{M})(\psi)|U_\psi\rangle\\
&\quad + (1-W_{\mathbb{O}}(\mathcal{E}))\,\langle U_\psi|\mathrm{id}\otimes\tilde{\Theta}(\mathcal{N})(\psi)|U_\psi\rangle\\
&\leq W_{\mathbb{O}}(\mathcal{E})F_{\mathbb{O}}^{\psi}(\mathcal{U})\,\mathrm{Tr}[\mathrm{id}\otimes\tilde{\Theta}(\mathcal{M})(\psi)]\\
&\quad + (1-W_{\mathbb{O}}(\mathcal{E}))\,\mathrm{Tr}[\mathrm{id}\otimes\tilde{\Theta}(\mathcal{N})(\psi)]\\
&\leq 1-(1-F_{\mathbb{O}}^{\psi}(\mathcal{U}))W_{\mathbb{O}}(\mathcal{E}).
\end{aligned}
\tag{115}
$$

To get the third line, we bound the first term in the second line by using (113) and identifying $t = \mathrm{Tr}[\mathrm{id}\otimes\tilde{\Theta}(\mathcal{M})(\psi)]$, which can be seen by taking trace in both sides of (114). We used a similar reasoning to bound the second term; for any channel $\mathcal{N}$, sub-superchannel $\tilde{\Theta}$, and state ψ, there exists another channel $\mathcal{N}'$ such that $\mathrm{id}\otimes\tilde{\Theta}(\mathcal{N})(\psi) = t\cdot\mathrm{id}\otimes\mathcal{N}'(\psi)$ with $t = \mathrm{Tr}[\mathrm{id}\otimes\tilde{\Theta}(\mathcal{N})(\psi)]$. Thus, we get $\langle U_\psi|\mathrm{id}\otimes\tilde{\Theta}(\mathcal{N})(\psi)|U_\psi\rangle = \mathrm{Tr}[\mathrm{id}\otimes\tilde{\Theta}(\mathcal{N})(\psi)]\,\langle U_\psi|\mathrm{id}\otimes\mathcal{N}'(\psi)|U_\psi\rangle \leq \mathrm{Tr}[\mathrm{id}\otimes\tilde{\Theta}(\mathcal{N})(\psi)]$. Finally, to get the final line we used $\mathrm{Tr}[\mathrm{id}\otimes\tilde{\Theta}(\mathcal{M})(\psi)]\leq 1$ and $\mathrm{Tr}[\mathrm{id}\otimes\tilde{\Theta}(\mathcal{N})(\psi)]\leq 1$.

To get the third bound, noting

$$
\mathrm{Tr}\left[\mathrm{id}\otimes\tilde{\Theta}(\mathcal{E})(\psi)\right] = W_{\mathbb{O}}(\mathcal{E})\,\mathrm{Tr}\left[\mathrm{id}\otimes\tilde{\Theta}(\mathcal{M})(\psi)\right] + (1-W_{\mathbb{O}}(\mathcal{E}))\,\mathrm{Tr}\left[\mathrm{id}\otimes\tilde{\Theta}(\mathcal{N})(\psi)\right],
\tag{116}
$$

we equate the right-hand side of the third line of (115) to $\mathrm{Tr}[\mathrm{id}\otimes\tilde{\Theta}(\mathcal{E})(\psi)] - (1-F_{\mathbb{O}}^{\psi}(\mathcal{U}))W_{\mathbb{O}}(\mathcal{E})\,\mathrm{Tr}[\mathrm{id}\otimes\tilde{\Theta}(\mathcal{M})(\psi)]$. Plugging in $\mathrm{Tr}[\mathrm{id}\otimes\tilde{\Theta}(\mathcal{E})(\psi)] = p$ leads to the inequality in the statement.

■

Proof of Prop. 22. Proofs for (109) and (111) are analogous to Thm. 21. To show (110), we use the following strong monotonicity property of $W_{\mathbb{F}}$.

Lemma 23 ([66]). *Let $\{\tilde{\mathcal{M}}_i\}$ be a free instrument, i.e., $\tilde{\mathcal{M}}_i(\sigma)\in\mathrm{cone}(\mathbb{F}),\forall\sigma\in\mathbb{F},\forall i$. Writing $p_i = \mathrm{Tr}[\tilde{\mathcal{M}}_i(\rho)]$, we get*

$$
W_{\mathbb{F}}(\rho) \leq \sum_i p_i W_{\mathbb{F}}\left(\frac{\tilde{\mathcal{M}}_i(\rho)}{p_i}\right)
\tag{117}
$$

and

$$R_{\mathbb{F}}(\rho) \geq \sum_i p_i R_{\mathbb{F}}\left(\frac{\tilde{\mathcal{M}}_i(\rho)}{p_i}\right) \tag{118}$$

Proof. Write $\rho \geq W_{\mathbb{F}}(\rho)\sigma$ with a free state $\sigma \in \mathbb{F}$ and some state τ. Then,

$$\frac{\tilde{\mathcal{M}}_i(\rho)}{p_i} \geq \frac{W_{\mathbb{F}}(\rho)}{p_i}\mathrm{Tr}[\tilde{\mathcal{M}}_i(\sigma)]\frac{\tilde{\mathcal{M}}_i(\sigma)}{\mathrm{Tr}[\tilde{\mathcal{M}}_i(\sigma)]}. \tag{119}$$

Since $\tilde{\mathcal{M}}_i(\sigma)/\mathrm{Tr}[\tilde{\mathcal{M}}_i(\sigma)] \in \mathbb{F}$, the above expression serves as a valid free decomposition of $\tilde{\mathcal{M}}_i(\rho)/p_i$. Thus, we get $W_{\mathbb{F}}(\tilde{\mathcal{M}}_i(\rho)/p_i) \geq \mathrm{Tr}[\tilde{\mathcal{M}}_i(\sigma)]W_{\mathbb{F}}(\rho)/p_i$. The statement is reached by multiplying by p_i and summing over i in both sides, as well as using $\sum_i \mathrm{Tr}[\mathcal{M}_i(\rho)] = 1$. The bound for $R_{\mathbb{F}}$ can be shown analogously. ■

Using Lem. 23 we get that

$$\begin{aligned} 1 - W_{\mathbb{F}}(\rho) &\geq 1 - \sum_i p_i W_{\mathbb{F}}\left(\frac{\tilde{\mathcal{M}}_i(\rho)}{p_i}\right) \\ &= \sum_i p_i \left[1 - W_{\mathbb{F}}\left(\frac{\tilde{\mathcal{M}}_i(\rho)}{p_i}\right)\right], \end{aligned} \tag{120}$$

namely, $1 - W_{\mathbb{F}}(\rho)$ also shows the strong monotonicity (with the opposite direction of inequality). Then, since one can always construct a free instrument by complementing $\tilde{\mathcal{M}}$ with a replacement subchannel $\tilde{\mathcal{R}}_\sigma$ defined by the Choi matrix $J_{\tilde{\mathcal{R}}_\sigma} = (\mathbb{1} - \mathrm{Tr}_B J_{\tilde{\mathcal{M}}}) \otimes \sigma$, $\sigma \in \mathbb{F}$, (120) implies that

$$1 - W_{\mathbb{F}}(\rho) \geq p\left[1 - W_{\mathbb{F}}\left(\frac{\tilde{\mathcal{M}}(\rho)}{p}\right)\right] \geq p\left[1 - \frac{\varepsilon}{1 - F_{\mathbb{F}}(\phi)}\right], \tag{121}$$

where in the last inequality we used Cor. 10. A simple reordering of the terms leads to the bound in the statement. ■

Supplementary References

[1] G. Chiribella, G. M. D'Ariano, and P. Perinotti, *Transforming quantum operations: Quantum supermaps,* EPL Europhys. Lett. **83**, 30004 (2008).
[2] X. Yuan, *Hypothesis testing and entropies of quantum channels,* Phys. Rev. A **99**, 032317 (2019).
[3] Y. Liu and X. Yuan, *Operational resource theory of quantum channels,* Phys. Rev. Research **2**, 012035 (2020).
[4] Z.-W. Liu and A. Winter, *Resource theories of quantum channels and the universal role of resource erasure,* arXiv:1904.04201 (2019).
[5] G. Gour and A. Winter, *How to Quantify a Dynamical Quantum Resource,* Phys. Rev. Lett. **123**, 150401 (2019).
[6] I. Devetak, A. W. Harrow, and A. J. Winter, *A Resource Framework for Quantum Shannon Theory,* IEEE Trans. Inf. Theory **54**, 4587 (2008).
[7] K. Fang, X. Wang, M. Tomamichel, and M. Berta, *Quantum channel simulation and the channel's smooth max-information,* IEEE Trans. Inf. Theory **66**, 2129 (2020).
[8] R. Takagi, K. Wang, and M. Hayashi, *Application of the Resource Theory of Channels to Communication Scenarios,* Phys. Rev. Lett. **124**, 120502 (2020).
[9] H. Kristjánsson, G. Chiribella, S. Salek, D. Ebler, and M. Wilson, *Resource theories of communication,* New J. Phys. **22**, 073014 (2020).
[10] D. Rosset, F. Buscemi, and Y.-C. Liang, *Resource Theory of Quantum Memories and Their Faithful Verification with Minimal Assumptions,* Phys. Rev. X **8**, 021033 (2018).
[11] X. Yuan, Y. Liu, Q. Zhao, B. Regula, J. Thompson, and M. Gu, *Universal and Operational Benchmarking of Quantum Memories,* arXiv:1907.02521 (2020).
[12] R. Takagi, *Optimal resource cost for error mitigation,* arXiv:2006.12509 [quant-ph] (2020).
[13] S. Endo, Z. Cai, S. C. Benjamin, and X. Yuan, *Hybrid quantum-classical algorithms and quantum error mitigation,* J. Phys. Soc. Jpn. **90**, 032001 (2021).
[14] M. Berta, F. G. S. L. Brandão, M. Christandl, and S. Wehner, *Entanglement Cost of Quantum Channels,* IEEE Trans. Inf. Theory **59**, 6779 (2013).
[15] S. Pirandola, R. Laurenza, C. Ottaviani, and L. Banchi, *Fundamental limits of repeaterless quantum communications,* Nat. Commun. **8**, 15043 (2017).

[16] M. Christandl and A. Müller-Hermes, *Relative Entropy Bounds on Quantum, Private and Repeater Capacities,* Commun. Math. Phys. **353**, 821 (2017).
[17] M. M. Wilde, *Entanglement cost and quantum channel simulation,* Phys. Rev. A **98**, 042338 (2018).
[18] G. Gour and C. M. Scandolo, *Entanglement of a bipartite channel,* Phys. Rev. A **103**, 062422 (2021).
[19] S. Bäuml, S. Das, X. Wang, and M. M. Wilde, *Resource theory of entanglement for bipartite quantum channels,* arXiv:1907.04181 (2019).
[20] K. Ben Dana, M. García Díaz, M. Mejatty, and A. Winter, *Resource theory of coherence: Beyond states,* Phys. Rev. A **95**, 062327 (2017).
[21] M. G. Díaz, K. Fang, X. Wang, M. Rosati, M. Skotiniotis, J. Calsamiglia, and A. Winter, *Using and reusing coherence to realize quantum processes,* Quantum **2**, 100 (2018).
[22] T. Theurer, D. Egloff, L. Zhang, and M. B. Plenio, *Quantifying Operations with an Application to Coherence,* Phys. Rev. Lett. **122**, 190405 (2019).
[23] T. Theurer, S. Satyajit, and M. B. Plenio, *Quantifying dynamical coherence with dynamical entanglement,* Phys. Rev. Lett. **125**, 130401 (2020).
[24] G. Saxena, E. Chitambar, and G. Gour, *Dynamical resource theory of quantum coherence,* Phys. Rev. Research **2**, 023298 (2020).
[25] P. Faist and R. Renner, *Fundamental Work Cost of Quantum Processes,* Phys. Rev. X **8**, 021011 (2018).
[26] J. R. Seddon and E. T. Campbell, *Quantifying magic for multi-qubit operations,* Proc. R. Soc. A **475**, 20190251 (2019).
[27] X. Wang, M. M. Wilde, and Y. Su, *Quantifying the magic of quantum channels,* New J. Phys. **21**, 103002 (2019).
[28] J. S. Bell, *On the problem of hidden variables in quantum mechanics,* Rev. Mod. Phys. **38**, 447 (1966).
[29] N. Brunner, D. Cavalcanti, S. Pironio, V. Scarani, and S. Wehner, *Bell nonlocality,* Rev. Mod. Phys. **86**, 419 (2014).
[30] J. I. de Vicente, *On nonlocality as a resource theory and nonlocality measures,* J. Phys. A: Math. Theor. **47**, 424017 (2014).
[31] E. Wolfe, D. Schmid, A. B. Sainz, R. Kunjwal, and R. W. Spekkens, *Quantifying Bell: the Resource Theory of Nonclassicality of Common-Cause Boxes,* Quantum **4**, 280 (2020).
[32] D. Schmid, D. Rosset, and F. Buscemi, *The type-independent resource theory of local operations and shared randomness,* Quantum **4**, 262 (2020).
[33] D. Rosset, D. Schmid, and F. Buscemi, *Type-independent characterization of spacelike separated resources,* Phys. Rev. Lett. **125**, 210402 (2020).
[34] B. S. Cirel'son, *Quantum generalizations of bell's inequality,* Lett. Math. Phys. **4**, 93 (1980).
[35] S. Popescu and D. Rohrlich, *Quantum nonlocality as an axiom,* Found. Phys. **24**, 379 (1994).
[36] J. Geller and M. Piani, *Quantifying non-classical and beyond-quantum correlations in the unified operator formalism,* J. Phys. A: Math. Theor. **47**, 424030 (2014).
[37] D. Schmid, H. Du, M. Mudassar, G. Coulter-de Wit, D. Rosset, and M. J. Hoban, *Postquantum common-cause channels: the resource theory of local operations and shared entanglement,* Quantum **5**, 419 (2021).
[38] S. Kochen and E. P. Specker, *The problem of hidden variables in quantum mechanics,* J. Math. Mech. **17**, 59 (1967).
[39] A. Cabello, S. Severini, and A. Winter, *Graph-theoretic approach to quantum correlations,* Phys. Rev. Lett. **112**, 040401 (2014).
[40] B. Amaral, A. Cabello, M. T. Cunha, and L. Aolita, *Noncontextual wirings,* Phys. Rev. Lett. **120**, 130403 (2018).
[41] C. Duarte and B. Amaral, *Resource theory of contextuality for arbitrary prepare-and-measure experiments,* J. Math. Phys. **59**, 062202 (2018).
[42] B. Regula and R. Takagi, *One-shot manipulation of dynamical quantum resources,* arXiv:2012.02215 (2020), Phys. Rev. Lett. (in press).
[43] V. P. Belavkin, G. M. D'Ariano, and M. Raginsky, *Operational distance and fidelity for quantum channels,* J. Math. Phys. **46**, 062106 (2005).
[44] A. Gilchrist, N. K. Langford, and M. A. Nielsen, *Distance measures to compare real and ideal quantum processes,* Phys. Rev. A **71**, 062310 (2005).
[45] A. Y. Kitaev, *Quantum computations: Algorithms and error correction,* Russ. Math. Surv. **52**, 1191 (1997).
[46] C. Fuchs and J. van de Graaf, *Cryptographic distinguishability measures for quantum-mechanical states,* IEEE Trans. Inf. Theory **45**, 1216 (1999).
[47] P. J. Bushell, *Hilbert's metric and positive contraction mappings in a Banach space,* Arch. Rat. Mech. Anal. **52**, 330 (1973).
[48] D. Reeb, M. J. Kastoryano, and M. M. Wolf, *Hilbert's projective metric in quantum information theory,* J. Math. Phys. **52**, 082201 (2011).
[49] P. Funk, *Über Geometrien, bei denen die Geraden die Kürzesten sind,* Math. Ann. **101**, 226 (1929).
[50] G. Vidal and R. Tarrach, *Robustness of entanglement,* Phys. Rev. A **59**, 141 (1999).
[51] F. G. S. L. Brandão and G. Gour, *Reversible framework for quantum resource theories,* Phys. Rev. Lett. **115**, 070503 (2015).
[52] A. W. Harrow and M. A. Nielsen, *Robustness of quantum gates in the presence of noise,* Phys. Rev. A **68**, 012308 (2003).
[53] F. G. S. L. Brandão and N. Datta, *One-shot rates for entanglement manipulation under non-entangling maps,* IEEE Trans. Inf. Theory **57**, 1754 (2011).
[54] C. Napoli, T. R. Bromley, M. Cianciaruso, M. Piani, N. Johnston, and G. Adesso, *Robustness of coherence: An operational and observable measure of quantum coherence,* Phys. Rev. Lett. **116**, 150502 (2016).
[55] A. Anshu, M.-H. Hsieh, and R. Jain, *Quantifying Resources in General Resource Theory with Catalysts,* Phys. Rev. Lett. **121**, 190504 (2018).
[56] R. Takagi, B. Regula, K. Bu, Z.-W. Liu, and G. Adesso, *Operational Advantage of Quantum Resources in Subchannel Discrimination,* Phys. Rev. Lett. **122**, 140402 (2019).
[57] R. Takagi and B. Regula, *General Resource Theories in Quantum Mechanics and Beyond: Operational Characterization via Discrimination Tasks,* Phys. Rev. X **9**, 031053 (2019).
[58] Z.-W. Liu, K. Bu, and R. Takagi, *One-Shot Operational Quantum Resource Theory,* Phys. Rev. Lett. **123**, 020401 (2019).

[59] B. Regula, K. Bu, R. Takagi, and Z.-W. Liu, *Benchmarking one-shot distillation in general quantum resource theories,* Phys. Rev. A **101**, 062315 (2020).
[60] J. R. Seddon, B. Regula, H. Pashayan, Y. Ouyang, and E. T. Campbell, *Quantifying Quantum Speedups: Improved Classical Simulation From Tighter Magic Monotones,* PRX Quantum **2**, 010345 (2021).
[61] B. Regula, L. Lami, G. Ferrari, and R. Takagi, *Operational Quantification of Continuous-Variable Quantum Resources,* Phys. Rev. Lett. **126**, 110403 (2021).
[62] M. Lewenstein and A. Sanpera, *Separability and Entanglement of Composite Quantum Systems,* Phys. Rev. Lett. **80**, 2261 (1998).
[63] R. Uola, T. Bullock, T. Kraft, J.-P. Pellonpää, and N. Brunner, *All Quantum Resources Provide an Advantage in Exclusion Tasks,* Phys. Rev. Lett. **125**, 110402 (2020).
[64] A. F. Ducuara and P. Skrzypczyk, *Operational Interpretation of Weight-Based Resource Quantifiers in Convex Quantum Resource Theories,* Phys. Rev. Lett. **125**, 110401 (2020).
[65] A. F. Ducuara, P. Lipka-Bartosik, and P. Skrzypczyk, *Multiobject operational tasks for convex quantum resource theories of state-measurement pairs,* Phys. Rev. Research **2**, 033374 (2020).
[66] B. Regula, *Convex geometry of quantum resource quantification,* J. Phys. A: Math. Theor. **51**, 045303 (2018).
[67] M. M. Wilde, M. Berta, C. Hirche, and E. Kaur, *Amortized channel divergence for asymptotic quantum channel discrimination,* Lett. Math. Phys. **110**, 2277 (2020).
[68] N. Datta, *Min- and Max-Relative Entropies and a New Entanglement Monotone,* IEEE Trans. Inf. Theory **55**, 2816 (2009).
[69] M. Sion, *On general minimax theorems,* Pac. J. Math. **8**, 171 (1958).
[70] S. Boyd and L. Vandenberghe, *Convex Optimization* (Cambridge University Press, New York, 2004).
[71] H. Barnum, E. Knill, and M. Nielsen, *On quantum fidelities and channel capacities,* IEEE Trans. Inf. Theory **46**, 1317 (2000).
[72] D. Kretschmann and R. F. Werner, *Tema con variazioni: Quantum channel capacity,* New J. Phys. **6**, 26 (2004).
[73] F. Buscemi and N. Datta, *The Quantum Capacity of Channels With Arbitrarily Correlated Noise,* IEEE Trans. Inf. Theory **56**, 1447 (2010).
[74] M. Horodecki, P. Horodecki, and R. Horodecki, *General teleportation channel, singlet fraction, and quasidistillation,* Phys. Rev. A **60**, 1888 (1999).
[75] K. Fang and Z.-W. Liu, *No-Go Theorems for Quantum Resource Purification,* Phys. Rev. Lett. **125**, 060405 (2020).
[76] G. Chiribella, G. M. D'Ariano, and P. Perinotti, *Quantum Circuit Architecture,* Phys. Rev. Lett. **101**, 060401 (2008).
[77] G. Chiribella, G. M. D'Ariano, and P. Perinotti, *Theoretical framework for quantum networks,* Phys. Rev. A **80**, 022339 (2009).
[78] G. Gutoski and J. Watrous, *Toward a general theory of quantum games,* in *Proceedings of the Thirty-Ninth Annual ACM Symposium on Theory of Computing*, STOC '07 (Association for Computing Machinery, New York, NY, USA, 2007) pp. 565–574.
[79] G. Chiribella, G. M. D'Ariano, P. Perinotti, and B. Valiron, *Quantum computations without definite causal structure,* Phys. Rev. A **88**, 022318 (2013).
[80] O. Oreshkov, F. Costa, and v. Brukner, *Quantum correlations with no causal order,* Nat. Commun. **3**, 1092 (2012).
[81] M. Araújo, A. Feix, M. Navascués, and C. Brukner, *A purification postulate for quantum mechanics with indefinite causal order,* Quantum **1**, 10 (2017).
[82] M. Horodecki, *Entanglement Measures,* Quant. Inf. Comput. **1**, 3 (2001).
[83] M. Hayashi, *Quantum Information Theory: Mathematical Foundation* (Springer, 2016).
[84] T. Cooney, M. Mosonyi, and M. M. Wilde, *Strong converse exponents for a quantum channel discrimination problem and quantum-feedback-assisted communication,* Commun. Math. Phys. **344**, 797 (2016).
[85] S. Bravyi, D. Browne, P. Calpin, E. Campbell, D. Gosset, and M. Howard, *Simulation of quantum circuits by low-rank stabilizer decompositions,* Quantum **3**, 181 (2019).
[86] X. Wang, M. M. Wilde, and Y. Su, *Efficiently Computable Bounds for Magic State Distillation,* Phys. Rev. Lett. **124**, 090505 (2020).
[87] S. Lloyd, *Capacity of the noisy quantum channel,* Phys. Rev. A **55**, 1613 (1997).
[88] H. Barnum, M. A. Nielsen, and B. Schumacher, *Information transmission through a noisy quantum channel,* Phys. Rev. A **57**, 4153 (1998).
[89] C. H. Bennett, P. W. Shor, J. A. Smolin, and A. V. Thapliyal, *Entanglement-assisted capacity of a quantum channel and the reverse Shannon theorem,* IEEE Trans. Inf. Theory **48**, 2637 (2002).
[90] I. Devetak, *The private classical capacity and quantum capacity of a quantum channel,* IEEE Trans. Inf. Theory **51**, 44 (2005).
[91] A. S. Holevo and R. F. Werner, *Evaluating capacities of bosonic Gaussian channels,* Phys. Rev. A **63**, 032312 (2001).
[92] C. H. Bennett, I. Devetak, A. W. Harrow, P. W. Shor, and A. Winter, *The quantum reverse shannon theorem and resource tradeoffs for simulating quantum channels,* IEEE Trans. Inf. Theory **60**, 2926 (2014).
[93] X. Wang, K. Fang, and R. Duan, *Semidefinite Programming Converse Bounds for Quantum Communication,* IEEE Trans. Inf. Theory **65**, 2583 (2019).
[94] M. Tomamichel, M. Berta, and J. M. Renes, *Quantum coding with finite resources,* Nat. Commun. **7**, 11419 (2016).
[95] M. Tomamichel, M. M. Wilde, and A. Winter, *Strong Converse Rates for Quantum Communication,* IEEE Trans. Inf. Theory **63**, 715 (2017).
[96] M. Berta and M. M. Wilde, *Amortization does not enhance the max-Rains information of a quantum channel,* New J. Phys. **20**, 053044 (2018).
[97] E. Kaur and M. M. Wilde, *Amortized entanglement of a quantum channel and approximately teleportation-simulable channels,* J. Phys. A: Math. Theor. **51**, 035303 (2017).
[98] S. Pirandola, *End-to-end capacities of a quantum communication network,* Commun. Phys. **2**, 51 (2019).
[99] K. Fang and H. Fawzi, *Geometric Rényi Divergence and its Applications in Quantum Channel Capacities,* Commun. Math. Phys. **384**, 1615 (2021).
[100] C. H. Bennett, D. P. DiVincenzo, J. A. Smolin, and W. K. Wootters, *Mixed-state entanglement and quantum error correction,* Phys. Rev. A **54**, 3824 (1996).

[101] T. Cubitt, D. Elkouss, W. Matthews, M. Ozols, D. Pérez-García, and S. Strelchuk, *Unbounded number of channel uses may be required to detect quantum capacity,* Nat. Commun. **6**, 6739 (2015).
[102] D. Leung and W. Matthews, *On the power of PPT-preserving and non-signalling codes,* IEEE Trans. Inf. Theory **61**, 4486 (2015).
[103] R. Duan and A. Winter, *No-signalling-assisted zero-error capacity of quantum channels and an information theoretic interpretation of the lovász number,* IEEE Trans. Inf. Theory **62**, 891 (2016).
[104] X. Wang, W. Xie, and R. Duan, *Semidefinite Programming Strong Converse Bounds for Classical Capacity,* IEEE Trans. Inf. Theory **64**, 640 (2018).
[105] X. Wang, K. Fang, and M. Tomamichel, *On Converse Bounds for Classical Communication Over Quantum Channels,* IEEE Trans. Inf. Theory **65**, 4609 (2019).
[106] G. Bowen, *Quantum feedback channels,* IEEE Trans. Inf. Theory **50**, 2429 (2004).
[107] M. Berta, M. Christandl, and R. Renner, *The quantum reverse shannon theorem based on one-shot information theory,* Commun. Math. Phys. **306**, 579 (2011).
[108] M. K. Gupta and M. M. Wilde, *Multiplicativity of completely bounded p-norms implies a strong converse for entanglement-assisted capacity,* Commun. Math. Phys. **334**, 867 (2015).
[109] E. Chitambar, D. Leung, L. Mančinska, M. Ozols, and A. Winter, *Everything You Always Wanted to Know About LOCC (But Were Afraid to Ask),* Commun. Math. Phys. **328**, 303 (2014).
[110] E. M. Rains, *A semidefinite program for distillable entanglement,* IEEE Trans. Inf. Theory **47**, 2921 (2001).
[111] E. M. Rains, *Entanglement purification via separable superoperators,* arXiv:quant-ph/9707002 (1997).
[112] A. Shimony, *Degree of Entanglement,* Ann. NY Ac. **755**, 675 (1995).
[113] M. Takeoka, S. Guha, and M. M. Wilde, *The Squashed Entanglement of a Quantum Channel,* IEEE Trans. Inf. Theory **60**, 4987 (2014).
[114] I. Devetak and A. Winter, *Distillation of secret key and entanglement from quantum states,* Proc. R. Soc. Lond. Math. Phys. Eng. Sci. **461**, 207 (2005).
[115] C. H. Bennett, A. W. Harrow, D. W. Leung, and J. A. Smolin, *On the capacities of bipartite Hamiltonians and unitary gates,* IEEE Trans. Inf. Theory **49**, 1895 (2003).
[116] S. Bäuml, S. Das, and M. M. Wilde, *Fundamental Limits on the Capacities of Bipartite Quantum Interactions,* Phys. Rev. Lett. **121**, 250504 (2018).
[117] L. Rigovacca, G. Kato, S. Bäuml, M. S. Kim, W. J. Munro, and K. Azuma, *Versatile relative entropy bounds for quantum networks,* New J. Phys. **20**, 013033 (2018).
[118] J. Preskill, *Reliable quantum computers,* Proc. Math. Phys. Eng. Sci. **454**, 385 (1998).
[119] A. M. Steane, *Error correcting codes in quantum theory,* Phys. Rev. Lett. **77**, 793 (1996).
[120] P. W. Shor, *Scheme for reducing decoherence in quantum computer memory,* Phys. Rev. A **52**, R2493 (1995).
[121] D. Gottesman, *Theory of fault-tolerant quantum computation,* Phys. Rev. A **57**, 127 (1998).
[122] A. G. Fowler, M. Mariantoni, J. M. Martinis, and A. N. Cleland, *Surface codes: Towards practical large-scale quantum computation,* Phys. Rev. A **86**, 032324 (2012).
[123] H. Bombin and M. A. Martin-Delgado, *Topological quantum distillation,* Phys. Rev. Lett. **97**, 180501 (2006).
[124] D. Gottesman and I. L. Chuang, *Demonstrating the viability of universal quantum computation using teleportation and single-qubit operations,* Nature **402**, 390 (1999).
[125] S. Bravyi and A. Kitaev, *Universal quantum computation with ideal clifford gates and noisy ancillas,* Phys. Rev. A **71**, 022316 (2005).
[126] S. Aaronson and D. Gottesman, *Improved simulation of stabilizer circuits,* Phys. Rev. A **70**, 052328 (2004).
[127] M. Amy, D. Maslov, and M. Mosca, *Polynomial-Time T-Depth Optimization of Clifford+T Circuits Via Matroid Partitioning,* IEEE Trans. Comput.-Aided Des. Integr. Circuits Syst. **33**, 1476 (2014).
[128] D. Gosset, V. Kliuchnikov, M. Mosca, and V. Russo, *An algorithm for the T-count,* Quantum Info. Comput. **14**, 1261 (2014).
[129] E. T. Campbell, B. M. Terhal, and C. Vuillot, *Roads towards fault-tolerant universal quantum computation,* Nature **549**, 172 (2017).
[130] V. Veitch, S. A. H. Mousavian, D. Gottesman, and J. Emerson, *The resource theory of stabilizer quantum computation,* New J. Phys. **16**, 013009 (2014).
[131] P. Selinger, *Quantum circuits of T-depth one,* Phys. Rev. A **87**, 042302 (2013).
[132] C. Jones, *Low-overhead constructions for the fault-tolerant Toffoli gate,* Phys. Rev. A **87**, 022328 (2013).
[133] C. Gidney, *Halving the cost of quantum addition,* Quantum **2**, 74 (2018).
[134] H. Pashayan, J. J. Wallman, and S. D. Bartlett, *Estimating Outcome Probabilities of Quantum Circuits Using Quasiprobabilities,* Phys. Rev. Lett. **115**, 070501 (2015).
[135] S. Bravyi, G. Smith, and J. A. Smolin, *Trading Classical and Quantum Computational Resources,* Phys. Rev. X **6**, 021043 (2016).
[136] M. Howard and E. Campbell, *Application of a Resource Theory for Magic States to Fault-Tolerant Quantum Computing,* Phys. Rev. Lett. **118**, 090501 (2017).
[137] G. Duclos-Cianci and K. M. Svore, *Distillation of nonstabilizer states for universal quantum computation,* Phys. Rev. A **88**, 042325 (2013).
[138] E. T. Campbell and M. Howard, *Unifying Gate Synthesis and Magic State Distillation,* Phys. Rev. Lett. **118**, 060501 (2017).
[139] M. Beverland, E. Campbell, M. Howard, and V. Kliuchnikov, *Lower bounds on the non-Clifford resources for quantum computations,* arXiv:1904.01124 (2019).
[140] D. Cavalcanti, F. G. S. L. Brandão, and M. O. Terra Cunha, *Are all maximally entangled states pure?* Phys. Rev. A **72**, 040303 (2005).
[141] M. Howard and J. Vala, *Qudit versions of the qubit $\pi/8$ gate,* Phys. Rev. A **86**, 022316 (2012).
[142] E. T. Campbell, H. Anwar, and D. E. Browne, *Magic-state distillation in all prime dimensions using quantum reed-muller codes,* Phys. Rev. X **2**, 041021 (2012).
[143] K. Fang and Z.-W. Liu, *No-go theorems for quantum resource purification: New approach and channel theory,* arXiv:2010.11822 (2020).
